# Depression and anxiety prevalence in people with cystic fibrosis and their caregivers: a systematic review and meta-analysis

**DOI:** 10.1007/s00127-022-02307-w

**Published:** 2022-06-04

**Authors:** Louise Lord, David McKernon, Luke Grzeskowiak, Sue Kirsa, Jenni Ilomaki

**Affiliations:** 1 Centre for Medicine Use and Safety, Faculty of Pharmacy and Pharmaceutical Sciences, Monash University, 381 Royal Parade, Melbourne, VIC 3052 Australia; 2 Pharmacy Department, Monash Health, Melbourne, VIC Australia; 3 Consultation Liaison Psychiatry, Monash Health, Melbourne, Australia; 4 College of Medicine and Public Health, Flinders University, Adelaide, Australia; 5 School of Public Health and Preventive Medicine, Monash University, Melbourne, Australia

**Keywords:** Depression, Anxiety, Cystic fibrosis, Prevalence, Psychological tests, Epidemiology

## Abstract

**Background:**

Prevalence of depression and anxiety in people with cystic fibrosis (PwCF) and their caregivers is high, however, results have been inconsistent. This systematic review and meta-analysis aimed to estimate the prevalence of depression and anxiety in PwCF and their caregivers and explore sources of heterogeneity.

**Method:**

MEDLINE, EMBASE, CINAHL plus and PsychINFO databases were searched from inception to January 2021. Studies were included if a specific psychometric tool (PT) to assess depression or anxiety (rather than quality of life) was used and did not involve a transitory patient state. Random-effects models were applied due to high anticipated heterogeneity and *I*^2 ^estimates were calculated. Sources of heterogeneity were explored through subgroup comparisons. The presence of small-study effects was investigated visually using funnel plots and statistically using the Egger test.

**Results:**

A total of 94 articles (48 full-text publications, 46 abstracts) were included. Depression prevalence in adolescents aged 12–18 years (*n* = 2386), adults (*n* = 9206) and caregivers (*n* = 6617) were 18.7% (95% CI 12.8–25.3%, *I*^2^ = 89.2%), 27.2% (95% CI 23.6–31%, *I*^2^ = 90.4%), and 32.8% (95% CI 27.9–37.9%, *I*^2^ = 90.3%), respectively. Anxiety prevalence in adolescents aged 12–18 years (*n* = 2142) was 26% (95% CI 19.6–33%, *I*^2^ = 86.4%), 28.4% (95% CI 25–31.9%, *I*^2^ = 85%) for adults (*n* = 8175), and 38.4% (95% CI 30.8–46.2%, *I*^2^ = 94.6%) for caregivers (*n* = 5931). Prevalence differed by the PT used and study location.

**Discussion:**

This comprehensive analysis found the prevalence of depression and anxiety in PwCF and their caregivers to be high, supporting recommendations for regular screening. Choice of PT significantly influenced prevalence, indicating a need for future studies to identify the optimal PT for each CF population to identify those most at risk.

**Supplementary Information:**

The online version contains supplementary material available at 10.1007/s00127-022-02307-w.

## Introduction

Cystic fibrosis (CF) management has been revolutionised in recent years, increasing life expectancy for those born in 2018 to over 50 years—a metamorphic difference from dying shortly after birth when CF was first defined in 1938 [1, 2]. CF management has subsequently shifted from that of a life-limiting condition to that of a chronic disease, with priorities changing to comorbidity screening and prevention [2]. As people with CF (PwCF) live a longer life, it is imperative that the quality of this life is optimised [2].

Chronic physical illness has long been associated with an increased risk of mental health issues such as depression [3]. Psychological burden in people with CF (PwCF) and their caregivers can result in poorer outcomes such as decreased lung function, lower body mass index and more hospitalisations, as well as higher health care costs and worse adherence to prescribed therapy [4–6]. International consensus statements for the screening and treatment of depression and anxiety in PwCF and their caregivers were published in 2016, and numerous studies investigating mental health in CF have since followed [7]. As with other rare conditions however, studies in CF are generally limited to small sample sizes, therefore pooled analyses may provide a method to highlight areas in need of attention which may only be identifiable with larger sample sizes. To our knowledge, there has been no meta-analysis which provides an overall picture of the prevalence of both depression and anxiety in PwCF of all ages, and caregivers in the CF community, combined. A comprehensive and improved understanding in this area will help direct services to those most at risk and take steps forward to optimising care.

Consequently, the primary objective of this systematic review and meta-analysis, was to estimate the prevalence of anxiety and depression among children, adolescents and adults with CF and their caregivers. Secondary objectives were to explore potential sources of heterogeneity in prevalence estimates by conducting pre-specified subgroup comparisons.

## Methods

This review and meta-analysis was conducted and reported in accordance with the Preferred Reporting Items for Systematic Reviews and Meta-Analyses (PRISMA) statements [8]. The study protocol was registered in the Prospero International Prospective Register of Systematic Reviews (PROSPERO Number CRD42020181826).

### Search strategy and selection criteria

Studies reporting the prevalence of depression or anxiety in PwCF or their caregivers were identified through an extensive literature search using MEDLINE, EMBASE, CINAHL plus and PsychINFO, from inception to 1st January 2021. For the purposes of this review, the term child or children, refers to PwCF 0–11 years, adolescent 12–18 years, and adult is PwCF over 18 years at the time of assessment. Caregiver describes a parent, guardian or anyone identifying themselves as a caregiver for a PwCF. Medical subject headings (MeSH), Emtree terms, keywords and truncated search terms related to depression or anxiety (depression, anxiety, anxiety disorder, mood, psych, mental health) and CF were combined. Searches were limited to English language and reference lists of identified studies were cross-checked for any additional studies. The full search strategy is shown in the supplementary material (Table S1–S3).

Studies of all designs were considered, including those whose primary publication was that of a conference or meeting abstract only provided they met inclusion criteria. Studies were included if (1) the participants had been diagnosed with CF or are the caregivers of a person who has been diagnosed with CF, (2) the article provided primary data of depression or anxiety prevalence measured using a validated psychometric tool (PT) specifically designed to assess depression or anxiety in the participant age group (as opposed to a more universal quality of life tool), and (3) the article was published or had subsequently been translated into, English. There was no limitation to age, sex, illness severity or location. Studies involving depression or anxiety relating to transitory states (e.g., medical procedures or investigations), during genetic screening, prenatal care or immediate time around CF diagnosis were excluded. Studies that included both participants who had, and had not undergone a solid organ transplant, were excluded if data could not be specifically identified for each group. Where multiple articles were identified arising from the same study sample, or if data were unclear, clarification was sought from authors and the article containing the most comprehensive information pertaining to the aims of this study was included. No specific exclusion criteria were set for participant numbers during the search, however, a minimum number of three or seven participants (for studies involving participants < 18 or ≥ 18 years of age, respectively) was set prior to analysis. Numbers were determined using a minimum sample size calculation based on expected prevalence values, conservatively taken from the findings of the largest study to date in this subject matter [9, 10].

One reviewer (LL) performed the full search strategy and removed duplicates. Titles and abstracts were independently screened by two reviewers (LL, DM). Full-text copies of articles were obtained if studies appeared to meet criteria or if it was unclear if they met inclusion criteria. Full-text articles were independently reviewed (LL, DM) for inclusion. Discrepancies were discussed with a third investigator (JI) until a consensus was reached.

### Data extraction and analysis

Data were independently extracted into a piloted and standardised data extraction tool by two reviewers (LL, DM), and cross-checked for accuracy. Data extracted included study details (author’s name, study design, sample size relevant to CF, country where study was undertaken, year of data collection and publication year), population details [mean age of PwCF (caregiver age not included, only the PwCF under their care), sex, history of solid organ transplant] and depression or anxiety PT details (name of PT used and scoring criteria applied). Prevalence was determined as per the authors of the original article, where the “case” of depression or anxiety was reported as per their criteria. Where studies included participants from two or more study groups (children, adolescents, adults or caregivers), data were extracted separately for each group. When data were not clearly reported in the article, but calculations were possible using the data provided, calculations were undertaken by two reviewers independently (LL, DM). Where data were reported in groups not aligned to the aims of this study, authors of the article were contacted for clarification and information provided via personal communication was used. If no response was obtained, the study was excluded.

Two reviewers (LL, DM) independently assessed the methodological quality of included full-text articles, using Joanna Briggs Institute (JBI) critical appraisal tools. JBI tools allow reviewers to select a specific checklist from the same toolkit based on study design and also provide a tool specific for appraising studies with prevalence objectives [11–13]. Any disagreements were resolved by a third investigator (JI). No studies were excluded from the meta-analysis based on quality, but subgroup analyses were performed on publication type (full-text article or abstract only) and a meta-regression was undertaken to explore risk of bias scores against reported prevalence.

The primary measures were the total pooled prevalence estimates of depression and anxiety for children, adolescents, adults and caregivers separately, using basic descriptors (percentage with 95% confidence interval, CI), and pooled using meta-analysis. Studies involving PwCF under 18 years of age were stratified according to the age of participants (0–11 and 12–18 years of age). Where the age range of participants was not stated or the range breached the pre-specified groups, the age category was recorded as “Unclear” and reported separately where possible. Pre-specified analyses were performed for different PT used, publication type (full-text publication or abstract only), study location (based on 2019–2020 World Bank income definitions [14]), transplant status and caregiver sex. Where studies used more than one PT to determine prevalence on the same population (i.e. each participant received two individual PT assessments, once from each PT), if an overall prevalence value was described, it was used for the overall prevalence, and the PT specific value was used for the PT specific analysis. If an overall prevalence was not provided, the higher prevalence was utilised in the overall analysis. A secondary sensitivity analysis was then performed using the lower reported prevalence to evaluate the impact on the overall pooled prevalence estimate. If more than one prevalence was provided from the same participant at different time points, either the most recent, or pre-intervention (for intervention studies) value was used for analysis. Binary outcome data were transformed via the Freeman-Tukey double arcsine transformation (and then back transformed where possible to show estimates and confidence intervals as percentages), ensuring studies reporting percentages of 0 or 100% were included in the meta-analysis [15]. Transformations also ensured that reported pooled prevalence did not fall outside of the valid range (0 to 100%). Summary measures were pooled using the ‘metaprop_one’ package in STATA 14 (StataCorp LP, College Station, TX) with 95% CIs estimated using the Wilson method [16]. Univariate meta-regressions of the effects of risk of bias as a continuous measure on study outcomes were performed using the ‘metafor’ and ‘metan’ packages in *R* (version 3.2.1, The *R* Foundation, Vienna, Austria)[17]. All summary estimates were specified using a random-effects model using the method of DerSimonian and Laird, with the estimate of heterogeneity being taken from the inverse-variance fixed-effect model. Between-study heterogeneity was quantified by the *I*^2^ statistic, with sources of heterogeneity explored through subgroup comparisons. The presence of small-study effects was investigated visually using funnel plots and statistically using the Egger test [18].

## Results

### Study characteristics

**Fig. 1:**
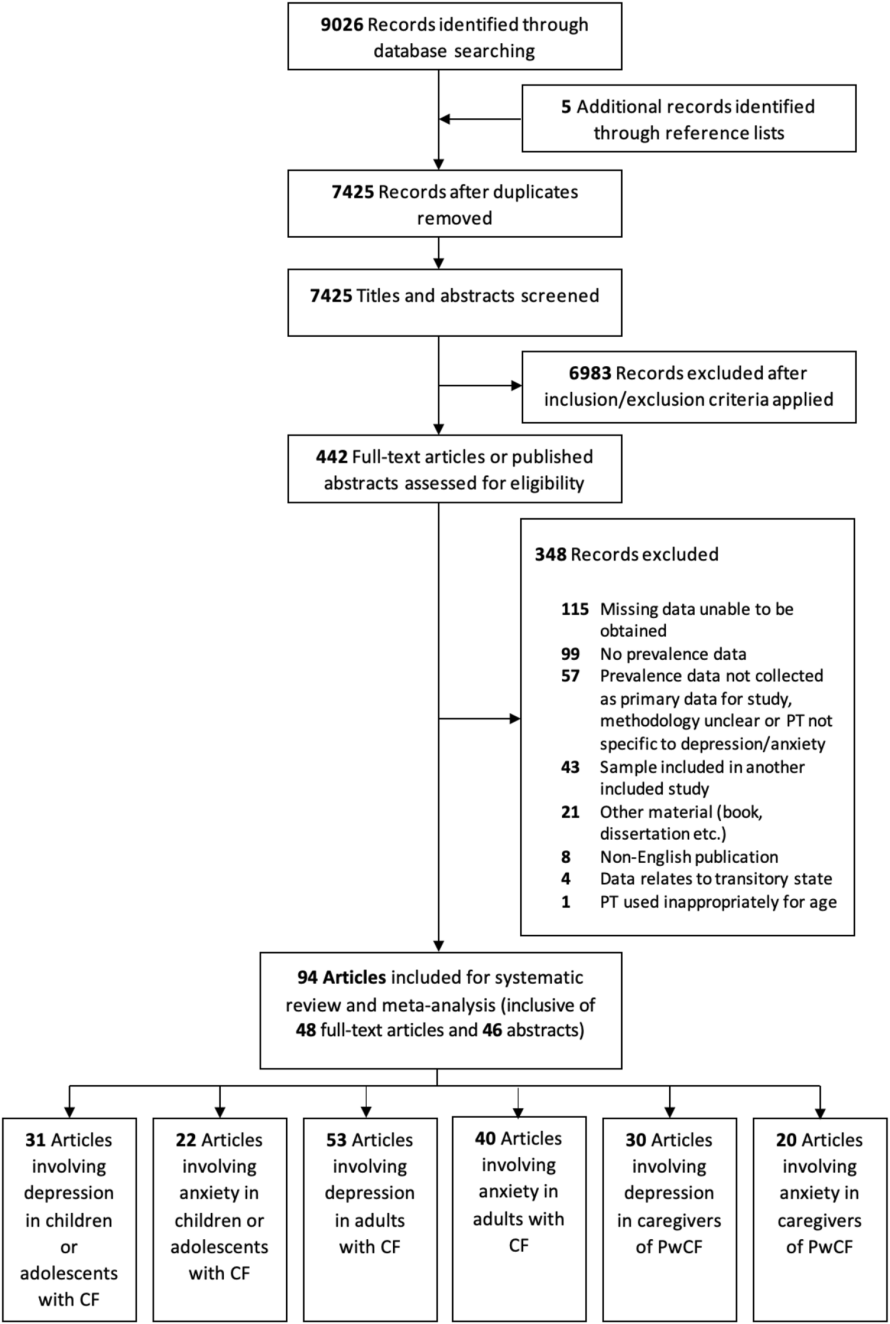
PRISMA flow diagram. *CF* cystic fibrosis, *PT* psychometric tool, *PwCF* people with cystic fibrosis

The search identified 7425 nonduplicate articles, of which 94 (48 full-text articles and 46 abstracts) were eligible for inclusion in the systematic review and meta-analysis (Fig. 1 and Table S4). Most studies focussed on one population group, however, 10 studies included two groups (either child or adolescent and adult PwCF, or child or adolescent PwCF and their caregiver) [19–28], and seven studies included all groups [10, 29–34].

Publication dates ranged from 1989 to 2020, with 91/94 articles published from 2004. Seventeen countries were represented, most frequently from North America (*n* = 51) and Europe (*n* = 31), and all but eight included studies being undertaken in a high-income country (HIC). The majority of included studies were observational and the age of participants ranged from 5 to 73 years in the 65/94 articles where an age range was provided.

### Quality assessment and risk of bias

No full-text article was deemed to be at high risk of bias and no studies were excluded from meta-analysis (Table S5–9). The use of Joanna Briggs quality appraisal tools provided comprehensive checklists for all study designs (including prevalence-based studies) and allowed for comparison during analysis. Meta-regression showed risk of bias was not significantly associated with reported prevalence in full publication articles, with p values consistently above 0.09 (Figs. S1–S6). Studies allocated a higher risk of bias (indicated by a lower risk of bias score) had similarities across unclear participant selection, baseline description or study setting. Funnel plots were produced to apply the Egger test for funnel asymmetry (Figs. S7–12). When examining the pooled prevalence of depression in children and adults with CF, the Egger test for funnel plot asymmetry indicated the presence of small-study effects (*p* = 0.023 and *p* = 0.001 respectively), with smaller studies reporting a higher prevalence of depression than larger studies (Figs. S7, S9). Funnel plot asymmetry was not identified using the Egger test for other outcomes (Figs. S8, S10–S12).

### Prevalence of depression

Depression was reported in 92/94 individual articles with children or adolescents, adults and caregivers being represented in 31, 53 and 30 articles respectively.

Three articles studying children aged 5–11 years (*n* = 97), reported prevalence ranging from 17.8 to 21.9%, compared with adolescents aged 12–18 having a pooled estimate of 18.7% (95% CI 12.8–25.3%, *I*^2^ = 89.2%) from 26 articles and 2386 participants (Fig. S13). Five articles involved participants that were not exclusive to either age category or not specified clearly and reported depression prevalence ranging from 4.5 to 28.9% (*n* = 239) (Fig. S13).

Adult pooled prevalence of depression was calculated at 27.2% (95% CI 23.6–31%, *I*^2^ = 90.4%) from 9206 participants (Fig. S14). Results from a sensitivity analysis, which used the lower of two prevalence estimates in studies with multiple PTs assessing depression, revealed a similar pooled estimate of 25.5% (95% CI 22–29.2%, *I*^2^ = 90.4%).

A total of 6617 caregivers were included to estimate a depression prevalence of 32.8% (95% CI 27.9–37.9%, *I*^2^ = 90.3%) (Fig. S15). Heterogeneity was high in all groups (*I*^2^ = 89.2–90.4%, Figs. S13–S15).

### Prevalence of anxiety

Anxiety was reported in 63/94 individual articles from 2342 children or adolescents (32 between 5 and 11 years of age), 8175 adults and 5931 caregivers of PwCF.

Overall pooled prevalence in adolescents between 12 and 18 years old was 26% (95% CI 19.6–33%, *I*^2^ = 86.4%). In children aged 5–11 years, one article reported a prevalence of 46% in 32 participants. Four articles (*n* = 168) with unclear participant age ranges reported anxiety prevalence from 6.6 to 52.6% (Fig. S16).

Adult anxiety prevalence was calculated at 28.4% (95% CI 25–31.9%, *I*^2^ = 85%) and caregivers at 38.4% (95% CI 30.8–46.2%, *I*^2^ = 94.6%), respectively (Figs. S17–18). All analyses had a high level of heterogeneity (*I*^2^ = 85–94.6%, S16–S18).

### Investigation of heterogeneity

#### Psychometric tool

**Fig. 2:**
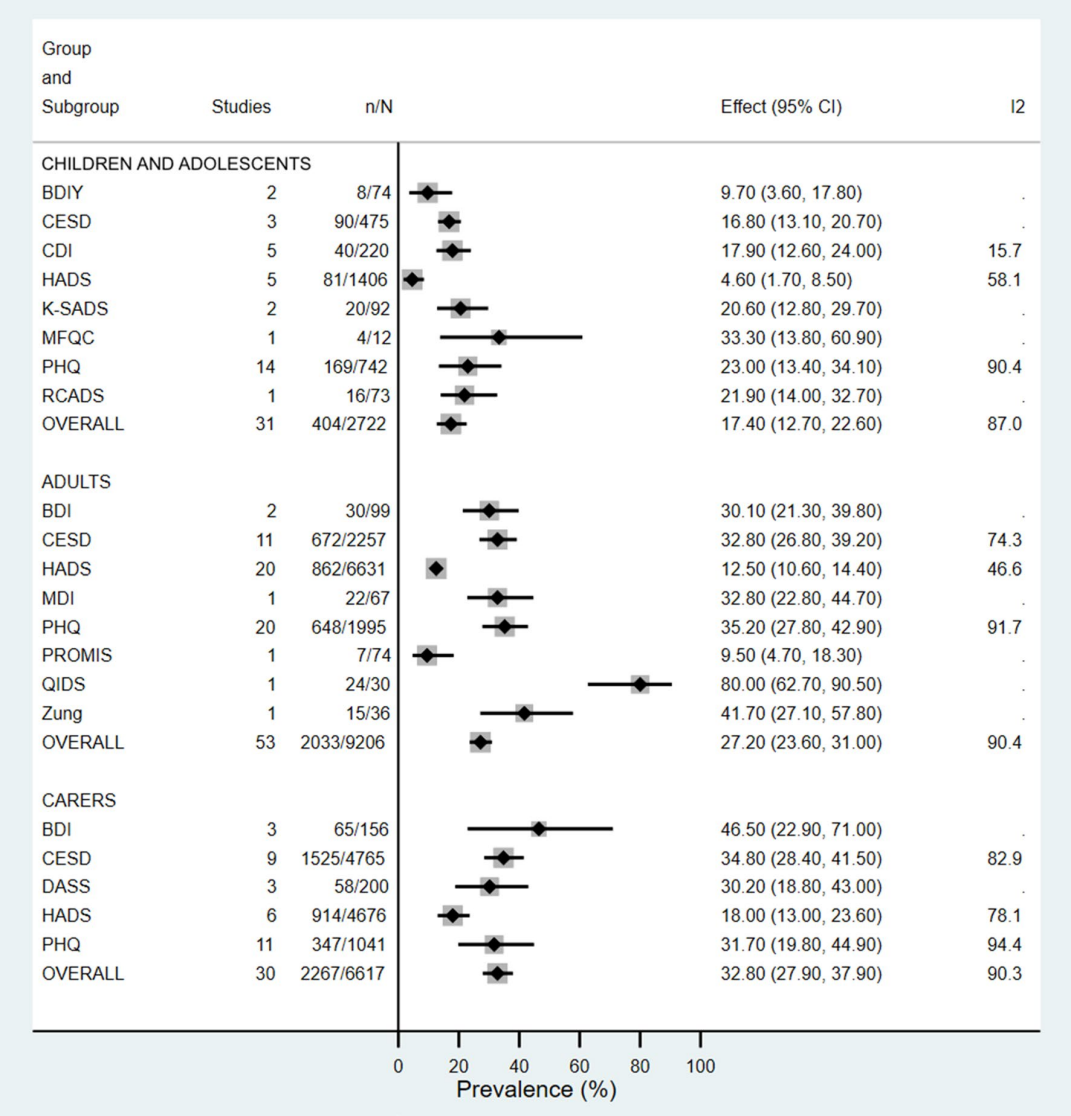
Prevalence of depression in children, adolescents and adults with cystic fibrosis and their caregivers, according to psychometric tool. *BDI* Beck Depression Inventory, *BDIY* Beck Depression Inventory for Youth, *CESD* Center for Epidemiologic Studies Depression Scale, *CDI* Children’s Depression Inventory, *CI* Confidence Interval, *DASS* Depression Anxiety Stress Scale, *ES* Effect Size, *HADS* Hospital Anxiety and Depression Scale, *K- SADS* Kiddie Schedule for Affective Disorders and Schizophrenia, *MDI* Major Depression Inventory, *MFQC* Mood and Feelings Questionnaire for Children, *PROMIS* PROMIS Depression Short Form, *PHQ* Patient Health Questionnaire, *QIDS* Quick Inventory of Depressive Symptomatology, *RCADS* Revised Children's Anxiety and Depression Survey, *Zung* Zung Self-Rating Depression Scale

**Fig. 3:**
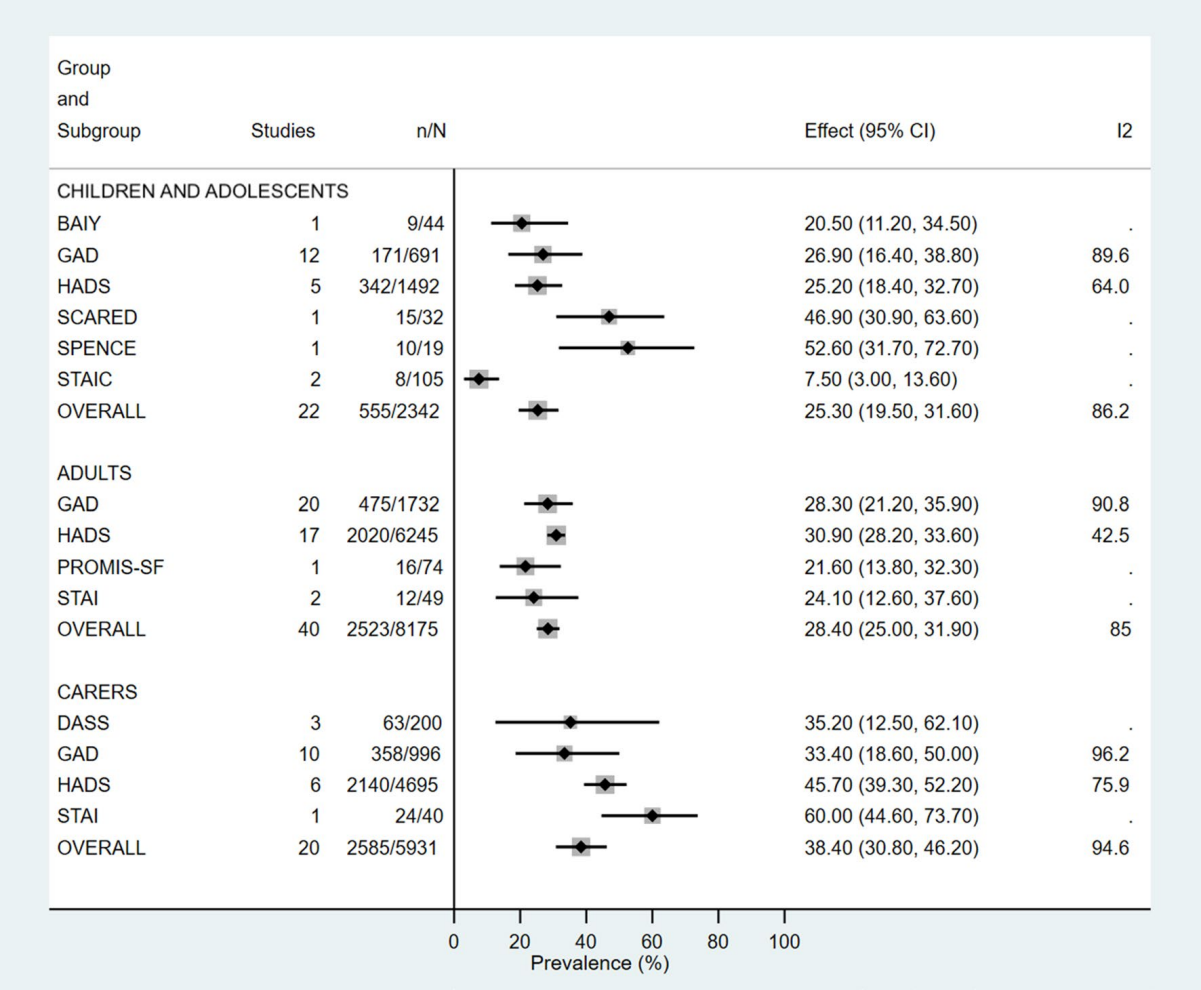
Prevalence of anxiety in children, adolescents and adults with cystic fibrosis and their caregivers, according to psychometric tool. *BAIY* Beck Anxiety Inventory for Youth, *CI* Confidence Interval, *DASS* Depression Anxiety Stress Scale, *ES* Effect Size, *GAD* Generalized Anxiety Disorder 7-item measure, *HADS* Hospital Anxiety and Depression Scale, *PROMIS-SF* PROMIS Anxiety Short Form, *SCARED* Screen for Child Anxiety Related Disorders, *SPENCE* Spence Children’s Anxiety Scale, *STAI* State-Trait Anxiety Inventory, *STAIC* State-Trait Anxiety Inventory for Children

A total of 14 and 8 different PTs were used to determine depression and anxiety prevalence across all groups respectively (Figs. 2, 3, S19–S24). Across both depression and anxiety studies, the PT cut-score for determining prevalence was not specified in 20/94 (21%) articles, all but one being abstracts (Table S4). The three most frequently used PT across all groups to assess depression were the Patient Health Questionnaire (PHQ) (*n* = 34/92), Hospital Anxiety and Depression Scale (HADS) (*n* = 24/92), and Center for Epidemiologic Studies Depression Scale (CESD) (*n* = 19/92). Significant differences (*p* < 0.05) were observed between PT in all groups when assessing depression and where PT groups had a minimum of five articles included *I*^2^ estimates ranged from 15.7 to 94.4%. The estimated depression prevalence was higher when the PHQ was used compared to HADS or CESD in both adolescents and adults, whereas caregiver prevalence from PHQ was higher than that from HADS but slightly lower than that from CESD (Figs. S19, S21 and S23).

The Generalized Anxiety Disorder measure (GAD) was the most common PT used when assessing anxiety in all participant groups (*n* = 32/63), whilst HADS was used across the largest number of participants (*n* = 21/65; 12,498 participants). Anxiety estimates between GAD and HADS in adolescent and adult groups were comparable [adolescents: GAD 26.9% (95% CI 16.4–38.8%, *I*^2^ = 89.6%); HADS 25.2% (95% CI 18.4–32.7%, *I*^2^ = 64%)] and [adult GAD 28.3% (95% CI 21.2–35.9%, *I*^2^ = 90.8%); HADS 30.9% (95% CI 28.2–33.6%, *I*^2^ = 42.5%)], however, in caregivers the estimate with GAD was 33.4% (95% CI 18.6–50%, *I*^2^ = 96.2%) compared to 45.7% (95% CI 39.3–52.2%, *I*^2^ = 75.9%) with HADS (Figs. S20, S22 and S24).

#### Study location

**Fig 4.**
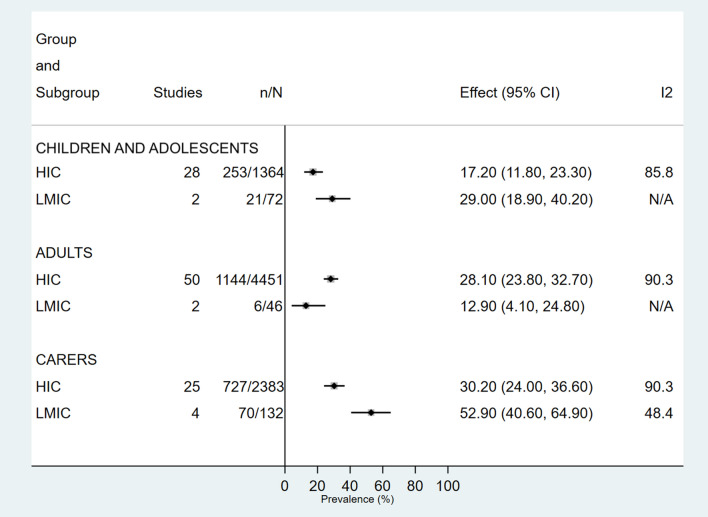
Prevalence of depression in children, adolescents and adults with cystic fibrosis and their caregivers, according to World Bank country income categories. *CI* Confidence Interval, *ES* Effect Size, *HIC* High income country, *LMIC* Low to middle-income country

**Fig 5.**
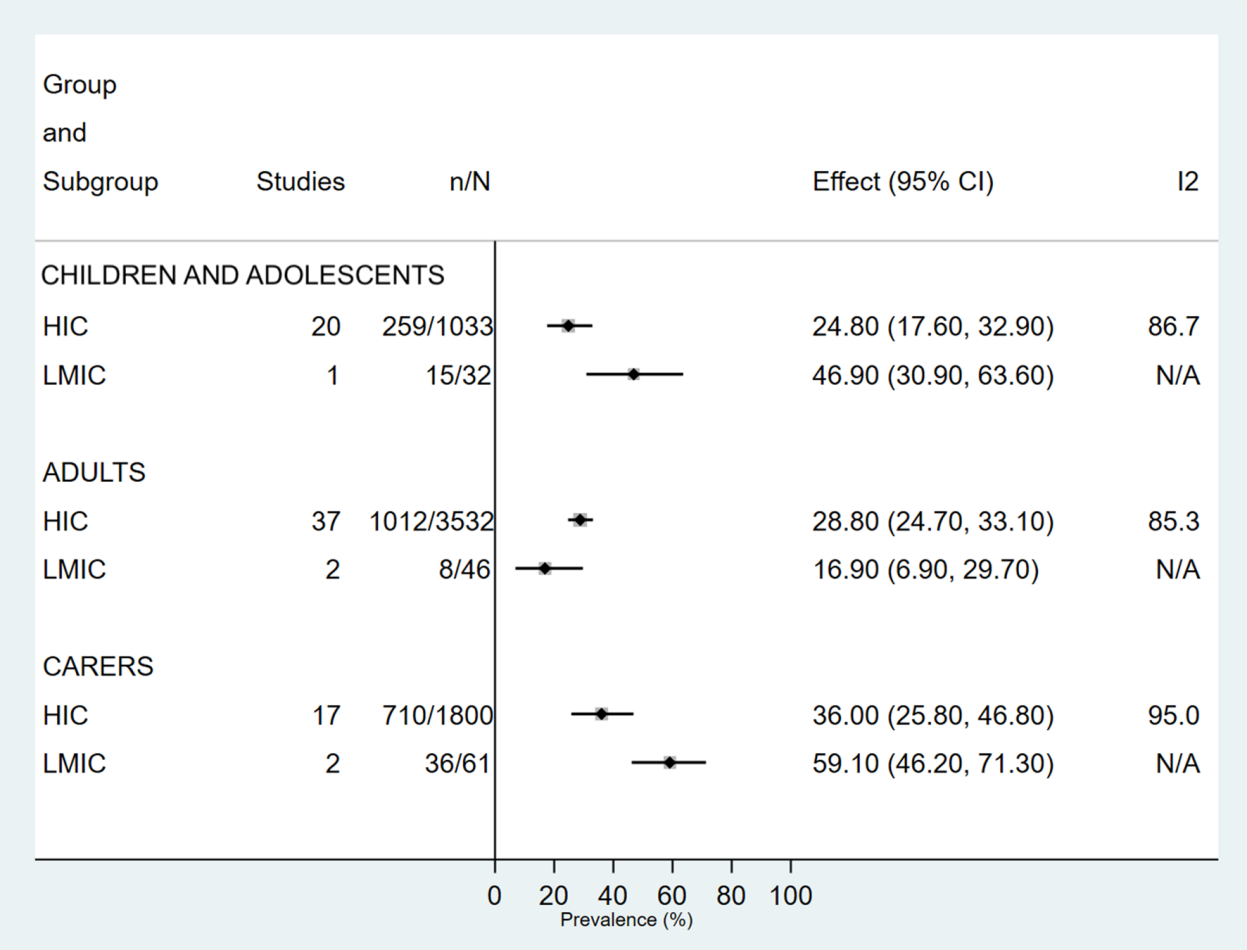
Prevalence of anxiety in children, adolescents and adults with cystic fibrosis and their caregivers, according to World Bank country income categories. *CI* Confidence Interval, *ES* Effect Size, *HIC* High income country, *LMIC* Low to middle-income country

After exclusion of one multinational study across different income categories, 13/17 (76.5%) countries were HIC. Prevalence estimates for both depression and anxiety in children, adolescents and caregivers were higher in low and middle-income countries (LMIC) compared with HIC. Adults in HIC had a higher prevalence of both conditions than those in LMIC. Heterogeneity in HIC across all groups and both conditions was high (*I*^2^ > 85.3%) (Figs. 4, 5, S25–S30).

#### Caregiver sex

Among six studies, depression prevalence was higher among female [44.8% (95% CI 33.4–56.5%, *I*^2^ = 93%)] compared with male [37.7% (95% CI 27.1–48.9%, *I*^2^ = 85.6%)] caregivers, but not statistically significant (*P* = 0.380) (Fig. S31). In the same six studies, anxiety prevalence appeared consistently higher among females [58.8% (95% CI 50.3–67.1%, *I*^2^ = 86.2%)] compared with males [48.9% (95% CI 36.5–61.3%, *I*^2^ = 88.3%)] but was also not statistically significant (*P* = 0.198) (Fig. S32).

#### Publication type

No statistically significant differences were shown between prevalence results in abstracts only compared with full-text articles across all groups and disorders, however, heterogeneity (indicated by the *I*^2^ statistic), remained above 82.8% in all groups (S33–S38).

## Discussion

This meta-analysis examined the prevalence of depression and anxiety in PwCF and their caregivers and explored the reasons for heterogeneity in results. The pooled prevalence estimates of depression and anxiety in adolescents 12–18 years of age (18.7%) and adults (27.2%) with CF were at least twice that of the general population [35] and higher than the largest individual CF study to date (10% and 19%) [10, 35]. The three articles that included children younger than 12 years, reported a range of depression prevalence (17.8–21.9%), also at least double of the general population [35]. The pooled anxiety prevalence in these groups was reported as at least four times that of the general population [35], but similar to that of the largest CF study [10]. Caregivers of PwCF also showed high prevalence across both disorders, with all groups displaying high heterogeneity across studies. Given the association of symptoms of depression and anxiety in PwCF with more hospitalisations, worse pulmonary function, poor therapy adherence and overall worse health outcomes, the need to understand what factors may influence these high and varied results, is paramount to identify areas for intervention [4, 6, 21, 36, 37].

Significant differences in reported prevalence were found according to the PT used. Depression in all groups was consistently lower when HADS was used as compared to all other PT. In 2015, Abbott et al. described how 48 different PT were used across CF centres in Europe, with HADS being the most common despite it being unvalidated in CF [38]. A later study across 727 PwCF aged 12–25 years, suggested using HADS in the CF population could lead to underestimation of depression, but overestimation of anxiety [39]. This is consistent with adult and caregiver groups in our analysis, which showed adult and caregiver anxiety prevalence of 30.9% and 45.7%, respectively, with HADS, compared to 28.3% and 33.4% with GAD—the most commonly used anxiety PT in our analysis. This highlights the clear differences in PT structures, strengths and weaknesses, and emphasises the need for clinicians to be aware of the influence and validity each PT has in the context of how it is applied and what population it is applied to. For instance, the use of HADS in a sample of over 700 PwCF aged between 12 and 25 years, was shown to be more indicative of overall emotional distress rather than specifically depression or anxiety [39]. Authors suggested this was due to the two-factor format of the tool being unable to appropriately discriminate between symptoms of depression and anxiety when all items were combined [39]. For this reason, clinicians may wish to consider an alternative tool if their aim is to screen specifically for depression or anxiety in PwCF.

The International Committee on Mental Health in Cystic Fibrosis consensus statements published in 2016 recommend the use of PHQ and GAD for depression and anxiety screening in adolescents above 12 years of age, adults and caregivers, based on established tool validity in these groups [7]. Our study had to exclude five child cohorts based on inappropriate use of either HADS, PHQ or GAD in those under 12 years of age (or the inability to identify if all participants fell within this range). Just three studies used age-appropriate PT for assessing depression in children 12 years and under. The two uses of the Children’s Depression Inventory (CDI) resulted in similar prevalence of 17.8% and 21.9%, however, the one use of the Kiddie Schedule for Affective Disorders (K-SADS) was much higher at 35%, emphasising that when comparing prevalence, PT is just one aspect to consider. In adolescents above 12 years where HADS or GAD was used age appropriately, anxiety rates in children with CF were comparable (HADS 25.2% and GAD 26.9%). The one article that involved those exclusively under 12 years of age, used the Screen for Child Anxiety Related Disorders (SCARED) PT and revealed a prevalence of 46.9%, while those with a cohort that was across the 5–18 year age range used PT such as the Spence Children’s Anxiety Scale (SCAS), Beck Anxiety Inventory for Youth or State-Trait Anxiety Inventory for Children. A recent systematic review of anxiety prevalence in children with CF, suggested anxiety in children is underestimated with a standardised measure such as GAD when compared to the gold standard diagnostic interviews [40]. In the study by Gundogdu et al. which used SCARED along with interviews, results were closely matched [41]. This could suggest SCARED as an appropriate PT for use in children under 12 years with CF, however, a firm recommendation cannot be made based on one study. Differences in depression and anxiety prevalence have been found in other analyses based on PT used [10, 40, 42, 43], and while clinician autonomy for PT choice should not be revoked, it is imperative there is a thorough understanding of the limitations each PT possess and the influence it may have on results. Given the small CF population and the need to often pool results for meaningful outcomes, our meta-analysis suggests that a consistent approach is needed.

Analysis for the effect of study location was hampered by the lack of studies in countries other than HIC (86/94), reflecting a global lack of information in mental health data in these countries [35]. Lack of appropriate mental health resources and services, health services that are not easily accessible and concern regarding discrimination and stigmatisation varies between countries and can affect the prevalence reported [35, 44]. CF specific disparities also exist between countries, and paucity in services such as CF psychology services may impact identification and intervention in this area [2]. Our study has highlighted the distinct gap of CF mental health prevalence in areas other than HIC, which warrants future investigation.

Worldwide data shows depression is more common in females (5.1%) than males (3.6%) as well as anxiety (4.6% and 2.6%) [35]. Our study also showed this albeit at much higher prevalence, but smaller difference between sex. Female caregivers had a higher prevalence of depression (44.8%) compared to males (37.7%). Anxiety prevalence also mirrored this (females 58.8% and males 48.9%). It must be noted however, that studies in CF have considerably more data surrounding female caregivers than males, with 3652 females included in the depression and anxiety analyses compared with 1213 males. HADS was also used in half of the studies analysed, which may impact results given HADS is intended for participants experiencing confounding health conditions and there was no mention of this in the caregiver group themselves. The lack of studies specifically isolating caregiver sex cannot result in robust conclusions, however, does identify the need for further investigation using appropriate PT for caregivers and focus on obtaining further data from male caregivers.

A unique aspect of this study was the inclusion of abstract-only data. A Cochrane review demonstrated that approximately only half of conference abstracts are published in full and that ‘positive’ results were more frequently published than ‘negative’ results [45]. Another study showed however, that the inclusion of abstracts had no impact on pooled estimates across different medical specialties [46]. In this analysis, 46 of 94 (48.9%) of included articles were abstract only. Across all populations in both depression and anxiety, analyses showed no statistically significant differences in pooled prevalence between abstract-only and full-text articles. This indicates that inclusion of abstract-only data in pooled analyses in CF, may provide a method of increasing sample sizes and generalisability of results concerning overall prevalence across a wide range of settings. Caution must be noted however, as detailed study descriptions are important for identifying key sources of study heterogeneity which abstracts may not be able to always provide.

### Strengths and limitations

This is the first meta-analysis of the prevalence of depression and anxiety inclusive of PwCF and their caregivers. It provides a comprehensive overview of pooled prevalence from studies to date, confirming the influence of PT on reported prevalence and urging a consistent and considered approach to PT selection. Smaller studies have compared PT, however, this study was able to include large participant numbers to fully explore their effect. This consolidated the need for consistent approaches in selection of PT, and specifically highlights the void of evidence for a clear recommendation for a specific PT for use in children under 12 years. The novel inclusion of abstract-only data makes this analysis unique and highlights how future studies should consider abstract-only data as a method to increase generalisability.

The impact of the global COVID-19 pandemic on the availability of authors to respond to information requests was evident and is believed to have contributed to the exclusion of some articles. Gaps in article data, such as not identifying the PT cut-off score or data collection periods, meant that more specific analyses were unable to be performed. Many studies did not provide detailed information about participant age and sex, further limiting analysis and limiting further investigation into the resolution of the high heterogeneity. Self-imposed restrictions on the methodology of our study must be acknowledged. Only articles available in English were included, excluding eight articles. The PT used had to be specific for depression or anxiety, however, CF centres may prefer to use a broader CF-related quality of life tool to provide a high-level screen for concerning symptoms. Procedural anxiety was also excluded from this analysis and therefore requires consideration outside the scope of this study.

## Conclusion

Mental health conditions are multifactorial and the high risk of depression and anxiety in PwCF and their caregivers found in this analysis, supports recommendations for regular screening. High levels of heterogeneity were observed, echoing the multifaceted nature of the conditions but also revealing how the choice of PT can significantly influence the reported prevalence. Clinicians must be aware of the intricacies and influences of each PT so to make an appropriate choice for CF and age. Future studies of rare conditions such as CF would benefit from consistent and thorough methodologies, to allow pooled data to help identify those at most risk as well as focussing on identifying the most appropriate tools for children under 12 years of age.

## Supplementary Information

Below is the link to the electronic supplementary material.

Supplementary file1 (PDF 6437 KB)

## Data Availability

Supplementary material provided. Corresponding author may be contacted for further information.
