# Supplementary material for: Depression and anxiety prevalence in people with cystic fibrosis and their caregivers: a systematic review and meta-analysis
Source: Soc Psychiatry Psychiatr Epidemiol. 2022 Jun 4;58(2):287–98. doi: 10.1007/s00127-022-02307-w (PMC9166202; doi:10.1007/s00127-022-02307-w)
Supplement: Supplementary file 1 — Supplementary file1 (PDF 6437 KB) [file 127_2022_2307_MOESM1_ESM.pdf]

Depression and anxiety prevalence in people with cystic fibrosis and their caregivers: a systematic review and meta-analysis.

Louise Lord, David McKernon, Luke Grzeskowiak, Sue Kirsa, Jenni Ilomaki
Corresponding author: Louise Lord, Centre for Medicine Use and Safety, Faculty of Pharmacy and Pharmaceutical Sciences, Monash University, Melbourne, Australia. louiseclaudialord@gmail.com

Social Psychiatry and Psychiatric Epidemiology

Supplementary Data

Table S1. Embase and Medline database search strategy

1. exp depression/ and exp depressive disorder/
2. exp anxiety/
3. exp anxiety disorder/
4. (mood or psychology or psychiatry or emotion or mental health or psychological distress or nervous or hypervigilance).mp. [mp=title, abstract, heading word, drug trade name, original title, device manufacturer, drug manufacturer, device trade name, keyword, floating subheading word, candidate term word]
5. exp psychotropic agent/
6. 1 or 2 or 3 or 4 or 5
7. cystic fibrosis.mp. or exp cystic fibrosis/
8. 6 and 7

Table S2. CINAHL plus database search strategy

1. MH “Depression+”
2. MW depressive disorder
3. MH “Anxiety+” OR MH “Anxiety Disorders+”
4. Mood OR psychology OR psychiatry OR emotion OR mental health OR psychological distress
5. S1 OR S2 OR S3 OR S4
6. MH “Cystic Fibrosis”
7. Cystic fibrosis
8. S6 OR S7
9. S5 AND S8

Table S3. PsychINFO database search strategy

1. exp "Depression (Emotion)"/ or exp Major Depression/
2. Anxiety Disorders/ or exp Anxiety/
3. (mood or psychology or psychiat* or emotion or mental health or psychological distress or nervous or hypervigilance).mp. [mp=title, abstract, heading word, table of contents, key concepts, original title, tests & measures, mesh]
4. limit 3 to human
5. 1 or 2 or 4
6. cystic fibrosis.mp. or exp Cystic Fibrosis/
7. 5 and 6

Table S4. Study characteristics of included studies presented by participant group

Source	Publication Type	Location, income status	Total CF Participants (% male)	Mean age years (SD or range)	Age category used for analysis (years)	Depression tool, cut score	Anxiety tool, cut score
**Children and Adolescents 0-18 years (36 studies)**							
Allgood, 2017 [1]	Abstract	USA, HIC	7 (NR)	NR	12-18	CESD, 16	-
Blair, 2018 [2]	Abstract	USA, HIC	23 (60)	19.7 (0.6)	12-18	PHQ9, 5	GAD7, 5
Burke, 1989 [3]	Full publication	USA, HIC	52 (52)	12.19 (3.22)	Unclear	K-SADS, NR	-
Canter, 2017 [4]	Abstract	USA, HIC	45 (NR)	NR (all ≥12)	12-18	PHQ9, NR	GAD7, NR
Cronly, Duff, 2019 [5]	Full publication	Ireland, HIC	33 (NR)	NR (14-18)	12-18	HADS, 8	HADS, 8‡
Duff, 2018 [6]	Abstract	UK, HIC	125 (NR)	NR (≤16)	12-18	PHQ8, NR	GAD7, NR
Gravelle, 2018 [7]	Abstract	Canada, HIC	33 (70)	NR	12-18	PHQ9, NR	GAD7, NR
Graziano, 2017 [8]	Abstract	Italy, HIC	47 (36)	5.6 (1-11) children (N=67)<br>14.2 (12-17) adolescent (N=47)	12-18	PHQ9, 5	GAD7, 5
Graziano, 2020 [9]	Full publication	Italy, HIC	54 (37)	12-18	12-18	PHQ9, 5	GAD7, 5
Gundogdu, 2019 [10]	Full publication	Turkey, UMIC	32 (63)	8-16	5-11	CDI, 13	SCARED, 25
Harris, 2017 [11]	Abstract	USA, HIC	60 (depression); 28 (anxiety) (NR)	NR	12-18	PHQ9, NR	GAD7, NR
Hilliard, 2015 [12]	Full publication	USA, HIC	10 (NR)	16-18	12-18	CESD‡, 16	-
Hong, 2014 [13]	Abstract	USA, HIC	73 (44)	15.6 (NR)	12-18	RCADS, NR	-
Hosseinzadeh, 2019 [14]	Full publication	Iran, UMIC	40 (60)	5-18	5-11<br>12-18	K-SADS-PL, NR	††
Iturralde, 2017 [15]	Full publication	USA, HIC	27 (60)	12-22	12-18	PHQ9, 10	-
Laine, 2018 [16]	Abstract	Sweden, HIC	44 (50)	15 (10-18)	Unclear	BDI-Youth, NR	BAI-Youth, NR
Lee, 2017 [17]	Abstract	USA, HIC	81 (NR)	NR	12-18	PHQ9, NR	GAD7, NR
Liu, 2020 [18]	Full publication	USA, HIC	110 (NR)	12-17	12-18	PHQ9, 5	GAD7, 5
McLean, 2014 [19]	Abstract	USA, HIC	103 (41)	15.8 (2.9)	12-18	HADS, NR	HADS, NR
Modi, 2011 [20]	Full publication	USA, HIC	42 (NR)	12-18	12-18	HADS, 8‡	HADS, 8‡
Oliver, 2014 [21]	Full publication	USA, HIC	28 (NR)	NR (14-18)	12-18	HADS, 8‡	HADS, 8‡
Paida, 2020 [22]	Abstract	Australia, HIC	19 (NR)	8.2 (5)	Unclear	-	SCAS, NR
Pearson, 1991 [23]	Full publication	USA, HIC	61 (56)	10.96 (8-15)	Unclear	CDI, 13	STAI-Children, 1 Standard

							deviation above the mean
Pumariega, 1993 [24]	Full publication	USA, HIC	44 (60)	11 (7-15)	Unclear	CDI, 13	STAI-Children, 1 Standard deviation above the mean
Quittner, 2014 [25]	Full publication	Multiple	1286 (47)	14.84 (1.69)	12-18	HADS, 8; CESD§, 16	HADS, 8
Quittner, 2016 [26]	Abstract	USA, HIC	11 (NR)	14.9 (12-17)	12-18	PHQ9, 5	GAD7, 5
Smith, 2014 [27]	Full publication	USA, HIC	38 (47)	NR (7-17)	Unclear	CDI, 13	-
Smith, 2015 [28]	Abstract	USA, HIC	24 (NR)	NR	12-18	PHQ9, 5	-
Vandeleur, 2018 [29]	Full publication	Australia, HIC	75 (NR)	NR (7-18)	5-11<br>12-18	CDI, 55; BDI-Youth, 55	-
Verkleij, 2018 [30]	Full publication	Netherlands, HIC	17 (46)	NR (12-18)	12-18	PHQ9, 5	GAD7, 5
Walsh, 2017 [31]	Abstract	USA, HIC	83 (NR)	NR (12-18)	12-18	PHQ9, 5	GAD7, 5
Wray, 2004 [32]	Full publication	UK, HIC	Pre-transplant:12 (NR)†	NR (8-17)	12-18	MFQ-Children, 27; MFQ-Parent, 21	-
			Post-transplant: 11 (NR)	12-18	NA		
**Adults (above 18 years) (54 studies)**							
Afshar 2014 [33]	Abstract	USA, HIC	63 (65)	33.2 (NR)		PHQ9,5	-
Allgood, 2017 [1]	Abstract	USA, HIC	68 (NR)	NR		CESD, 16	-
Askew, 2017 [34]	Full publication	UK, HIC	45 (60)	20.7 (17-24)		HADS, 8	HADS, 8
Beenen, 2016 [35]	Abstract	USA, HIC	215 (NR)	NR		PHQ9, 5	GAD7, 5
Bowen, 2019 [36]	Abstract	USA, HIC	14 (NR)	36.2 (16-54)		PHQ9, 5	GAD7, 5
Bruschwein, 2018 [37]	Abstract	USA, HIC	19 (NR) (post admission results)	NR		-	GAD7, 5
Burge, 2015 [38]	Full publication	Australia, HIC	80 (100)	30 (8)		HADS, 8‡	HADS, 8‡
Catastini, 2018 [39]	Abstract	Italy, HIC	113 (57)	34.8 (9.7)		PHQ9, 5	GAD7, 5
Christon, 2017 [40]	Abstract	USA, HIC	95 (NR)	33 (18-73)		PHQ9, 5	GAD7, 5
Cronly, Duff, 2019 [5]	Full publication	Ireland, HIC	141 (NR)	32.2 (7.3) and 28.2 (10.6)		HADS, 8	HADS, 8‡
Delelis, 2008 [41]	Full publication	France, HIC	13 (NR)	28 (4.56)		CESD-French version, Male:17 Female 23	STAI-French version, Male:37 Female: 40
			3 with transplant (NR)				
Dvorak, 2013 [42]	Abstract	USA, HIC	100 (NR)	NR		CESD, 16	-

Fukushima, 2013 [43]	Abstract	USA, HIC	23 (70)	28.9 (19-50)		BDI, 17	-
Graziano, 2017 [8]	Abstract	Italy, HIC	90 (46)	27.4 (18-52)		PHQ9, 5	GAD7, 5
Graziano, 2020 [9]	Full publication	Italy, HIC	113 (44)	27 (8.2)		PHQ9, 5	GAD7, 5
Havermans, 2008 [44]	Full publication	Belgium, HIC	57 (51)	26.7 (8.1)		HADS, 8	HADS, 8
Havermans, 2011 [45]	Abstract	Belgium, HIC	35 (54)	NR		HADS, 8	HADS, 8
Hayee, 2019 [46]	Full publication	UK, HIC	107 (44)	28.8 (10.1) and 29.1 (8.8)		PHQ9, 10	GAD7, 10
Henderson, 2020 [47]	Full publication	Australia, HIC	39 (NR)	30 (10)		PHQ9, 5‡	GAD7, 5
Hilliard, 2015 [12]	Full publication	USA, HIC	128 (NR)	29.2 (16-63)		CESD‡, 16	-
Hjelm, 2017 [48]	Abstract	USA, HIC	189 (NR)	NR		PHQ9, NR	GAD7, NR
Iliza, 2014 [49]	Abstract	Canada, HIC	205 (49)	31.9 (9.2)		HADS, 8	HADS, 8
Knudsen, 2016 [50]	Full publication	Denmark, HIC	67 (41)	24.1 (18-30)		MDI, 20	-
Kopp, 2012 [51]	Abstract	USA, HIC	20 (NR)	26.9		CESD, 16	-
Kopp, 2013 [52]	Full publication	USA, HIC	30 (63)	26.1 (7)		CESD, 16	-
Kopp, 2016 [53]	Full publication	USA, HIC	30 (57)	27.7 (8.5)		QIDS-Self Report, 6; QIDS-Clinician, 6	-
Lalic, 2018 [54]	Abstract	Croatia, HIC	22 (NR)<br>6 with transplant (NR)	NR		PHQ9, 5	GAD7, 5
Lambrecht, 2011 [55]	Abstract	Belgium, HIC	324 (NR)	NR		HADS, 8	HADS, 8
Latchford, 2013 [56]	Full publication	UK, HIC	232 (57)	NR		HADS, 8; PHQ9, 5	-
Lee, 2017 [17]	Abstract	USA, HIC	105 (NR)	NR		PHQ9, NR	GAD7, NR
Modi, 2011 [20]	Full publication	USA, HIC	17 (NR)	NR		HADS, 8‡	HADS, 8‡
Oliver, 2014 [21]	Full publication	USA, HIC	44 (NR)	19.1 (3.3)		HADS, 8‡	HADS, 8‡
Olveira, Giron 2010 [57]	Abstract	Spain, HIC	79 (47)	25.9 (7.7)		HADS, 8<br>CESD, 16	HADS, 8
Olveira, Olveira 2010 [58]	Abstract	Spain, HIC	43 (NR)	NR		HADS, 8<br>CESD, 16	HADS, 8
Orava, 2018 [59]	Full publication	Canada, HIC	22 (46)	33 (18-67)		HADS, 8‡	-
Pakhale, 2015 [60]	Full publication	Canada, HIC	45 (58)	30.7 (10.8)		CESD, 16	GAD7, 10
Pearson, 1991 [23]	Full publication	USA, HIC	36 (56)	24.81 (16-40)		Zung, 50	STAI, 1 Standard deviation above the mean
Quittner, 2014 [25]	Full publication	Multiple	4739 (52)	28.87 (9.5)		HADS, 8; CESD§, 16	HADS, 8

Quittner, 2016 [26]	Abstract	USA, HIC	56 (NR)	31.7 (18-72)		PHQ9, 5	GAD7, 5
Quon, 2015 [61]	Full publication	USA, HIC	153 (50)	28.6 (8.8)		PHQ9, 10	GAD7, 10
Rached, 2011 [62]	Abstract	Brazil, UMIC	16 (56)	27.9		HADS, NR	HADS, NR
Riekert, 2007 [63]	Full publication	USA, HIC	76 (45)	NR		BDI, 10	-
Rightmer, 2017 [64]	Abstract	USA, HIC	65 (NR)	NR		PHQ9, 5	GAD7, 5
Sandage, 2015 [65]	Abstract	USA, HIC	59 (37)	25		PHQ9, NR	GAD7, NR
Sherman, 2020 [66]	Full publication	USA, HIC	69 (57)	27.51 (9.62)		HADS, 8	-
Smith, 2015 [28]	Abstract	USA, HIC	89 (NR)	NR		PHQ9, 5	-
Talbot, 2012 [67]	Abstract	UK, HIC	326 (48)	30.9 (10.26)		HADS, NR	HADS, NR
Uslu, 2018 [68]	Abstract	Turkey, UMIC	30 (43)	24 (4)		HADS, 8	HADS, 10
Walker, 2015 [69]	Abstract	USA, HIC	74 (47)	NR		PDSF, NR	PASF, NR
Weldon, 2019 [70]	Abstract	UK, HIC	101 (NR)	NR		PHQ9, NR	GAD7, NR
Westell, 2014 [71]	Abstract	Canada, HIC	58 (NR)	NR		CESD, 16	GAD7, NR
Wolfe, 2016 [72]	Abstract	USA, HIC	60 (NR)	NR		PHQ9, NR	GAD7, NR
Wood, 2020 [73]	Full publication	Australia, HIC	60 (41)	31 (9)		HADS, 8‡	HADS, 8‡
Yohannes, 2012 [74]	Full publication	UK, HIC	121 (54)	30 (18-70)		HADS, 8‡	HADS, 8‡
**Caregivers (30 studies)**							
Akca, 2016 [75]	Abstract	Turkey, UMIC	40 (0)	NR		BDI, 10	STAI, NR
Barker, 2016 [76]	Full publication	USA, HIC	83 (NR)	NR		CESD, 16	-
Beinke, 2016 [77]	Full publication	Australia, HIC	51 (0)	48.83 (4.34)		DASS, 10	DASS, 10
Betz, 2019 [78]	Full publication	USA, HIC	23 (22)	NR		CESD, 16	-
Bhat, 2018 [79]	Full publication	India, LMIC	35 (66)	Mothers 27.8 (4.7) Fathers 30.9 (5.4)		CESD, 16	-
Branch-Smith, 2018 [80]	Abstract	Australia, HIC	47 (NR)	NR		DASS, NR	DASS, NR
Casana-Granell, 2018 [81]	Full publication	Spain, HIC	13 (NR)	NR		HADS, 8‡	HADS, 8‡
Cronly, Horgan, 2019 [82]	Full publication	Ireland, HIC	203 (25)	29.33 (6.6)		HADS, 8; CESD, 16	HADS, 8
Driscoll, 2010 [83]	Full publication	USA, HIC	87 (13)	36.61 (8.4)		CESD, 16	-
Duff, 2018 [6]	Abstract	UK, HIC	143 (NR)	NR		PHQ8, NR	GAD7, NR
Glasscoe, 2007 [84]	Full publication	UK, HIC	80 (59)#	NR		BDI, 13	-
Goetz, 2016 [85]	Abstract	USA, HIC	41 (NR)	NR		PHQ9, NR	-
Goodfellow, 2015 [86]	Full publication	UK, HIC	90 (NR)	NR		CESD, 16	-
Gravelle, 2018 [7]	Abstract	Canada, HIC	20 (30)	NR		PHQ9, NR	GAD7, NR
Graziano, 2017 [8]	Abstract	Italy, HIC	168 (38)	Mothers 40.1 (22-58); Fathers 41.1 (24-53)		PHQ9, 5	GAD7, 5

Graziano, 2020 [9]	Full publication	Italy, HIC	186 (37)	Mothers 41 (7.5) Fathers 41 (7.1)		PHQ9, 5	GAD7, 5
Grossoehme, 2015 [87]	Full publication	USA, HIC	142 (28)	NR		CESD, 16	-
Lambrecht, 2011 [55]	Abstract	Belgium, HIC	348 (31)	NR		HADS, 8	HADS, 8
Lee, 2017 [17]	Abstract	USA, HIC	196 (depression); 192 (anxiety) (NR)	NR		PHQ9, NR	GAD7, NR
Modi, 2011 [20]	Full publication	USA, HIC	40 (10)	43.18 (5.5)		HADS, 8‡	HADS, 8‡
Naranjo, 2017 [88]	Abstract	USA, HIC	102 (NR)	NR		PHQ9, 10	GAD7, 10
Quittner, 2014 [25]	Full publication	Multiple	4102 (24)	NR		HADS, 8; CESD§, 16	HADS, 8
Quittner, 2016 [26]	Abstract	USA, HIC	9 (NR)	NR		PHQ9, 5	GAD7, 5
Sheehan, 2012 [89]	Full publication	Australia, HIC	102 (54)	37.5 (26.6 - 61.6)		DASS, 10	DASS, 8
Szentpetery, 2018 [90]	Abstract	USA, HIC	54 (NR)	37.5 (9.1)		PHQ8, 5	GAD7, 5
Tluczek, 2014 [91]	Full publication	USA, HIC	33 (6)	48.1 (4.7) and 49.2 (7.9)		CESD, 16	-
Unal, 2020 [92]	Full publication	Turkey, UMIC	36 (0)	35.1 (1.2)		BDI, 10‡	-
Verkleij, 2018 [30]	Full publication	Netherlands, HIC	79 (42)	Mothers 40 (6.6); Fathers 42 (7.5)		PHQ9, 5	GAD7, 5
Walsh, 2018 [93]	Abstract	USA, HIC	29 (NR)	NR		PHQ9, 5	GAD7, 5
Yilmaz, 2008 [94]	Full publication	Turkey, UMIC	21 (0)	NR		HADS, 8‡	HADS, 8‡

Abbreviations; BAI: Beck Anxiety Inventory; BDI: Beck Depression Inventory; CESD: Center for Epidemiologic Studies Depression Scale; CDI: Children's Depression Inventory; DASS: Depression Anxiety Stress Scale; GAD7: Generalized Anxiety Disorder 7-item measure; HADS: The Hospital Anxiety and Depression Scale; K-SADS: Kiddie Schedule for Affective Disorders and Schizophrenia; K-SADS-PL: Kiddie Schedule for Affective Disorders and Schizophrenia-Present and Lifetime version; MDI: Major Depression Inventory; MFQ: Mood and Feelings Questionnaire; NR: not reported, unclear or not reported specifically for the CF subset used in this meta-analysis; PASF: PROMIS Anxiety Short Form. PDSF: PROMIS Depression Short Form; PHQ: Patient Health Questionnaire; QIDS: Quick Inventory of Depressive Symptomatology; RCADS: Revised Children's Anxiety and Depression Survey; SCARED: Screen for Child Anxiety Related Disorders; SCAS: The Spence Children's Anxiety Scale; STAI: State-Trait Anxiety Inventory; Unclear refers to studies where participant age is not exclusive to either 5-11 or 12-18 years of age.

†Data analysed from pre-transplantation participant sample. ‡ Data obtained from personal correspondence with author. §Data was not collected on both tools by all participants. ¶Cut score not comparable to other studies in this analysis. #Data analysed from 9-month sample. ††Multiple anxiety diagnoses reported, unable to differentiate between acute and chronic states, therefore not used in meta-analysis.

Risk of bias critical appraisal assessment

For sample size adequacy, a minimum number of three or seven participants (for studies involving participants <18 or ≥18 years of age, respectively) was set by reviewers prior to appraisal. Numbers were determined using a minimum sample size calculation based on expected prevalence values, conservatively taken from the findings of the largest study to date in this subject matter. [95,25] Unless an article scored less than 50% of the total score possible, it was deemed not at high risk of bias and was included for systematic review and meta-analysis. Note that for each question deemed not applicable for a study, the denominator for the overall score was lessened by 1, thereby not penalising the study for a lower score achieved.

Table S5. Risk of bias assessment for analytical cross-sectional studies

For sample size adequacy, a minimum number of three or seven participants (for studies involving participants <18 or ≥18 years of age, respectively) was set by reviewers prior to appraisal. Numbers were determined using a minimum sample size calculation based on expected prevalence values, conservatively taken from the findings of the largest study to date in this subject matter. [95,25] Unless an article scored less than 50% of the total score possible, it was deemed not at high risk of bias and was included for systematic review and meta-analysis.

Citation	Q1	Q2	Q3	Q4	Q5	Q6	Q7	Q8	Total / 8
Barker, 2016.	Y	Y	Y	Y	N	N	Y	Y	6
Beinke, 2017.	U	Y	Y	Y	Y	U	Y	Y	6
Bhat, 2018.	Y	N	Y	Y	Y	N	Y	Y	6
Branch-Smith, 2018.	U	N	Y	Y	N	N/A	Y	Y	4
Burge, 2015.	Y	Y	Y	Y	U	U	U	Y	4
Cronly, Duff, 2019.	Y	Y	Y	Y	Y	U	Y	Y	7
Delelis, 2008.	Y	Y	Y	Y	Y	Y	Y	Y	8
Goodfellow, 2015.	Y	Y	Y	Y	Y	Y	Y	Y	8
Grossoehme, 2015.	N	U	Y	Y	Y	N	U	Y	4
Havermans, 2008.	N	N	U	Y	Y	Y	Y	U	4
Hayee, 2019.	Y	U	Y	Y	N	U	Y	Y	5
Henderson, 2020.	U	Y	Y	Y	N	N	Y	Y	5
Hilliard, 2015.	Y	Y	Y	Y	U	U	Y	Y	6
Iturralde, 2017.	Y	U	Y	Y	Y	N	Y	Y	6
Liu, 2020.	Y	Y	Y	Y	U	U	Y	Y	6
Orava, 2018.	Y	Y	Y	Y	Y	Y	Y	Y	8
Pakhale, 2015.	Y	Y	Y	Y	N	N	Y	Y	6
Pumariega, 1993.	Y	N	Y	Y	N	N	Y	U	4
Riekert, 2007.	U	Y	Y	Y	N	N	Y	Y	5
Sherman, 2020.	Y	U	Y	Y	U	N/A	Y	Y	5
Smith, 2014.	Y	N	Y	Y	Y	U	Y	Y	6
Tluczek, 2014.	Y	Y	Y	Y	Y	U	Y	Y	7
Verkleij, 2018.	Y	Y	Y	Y	U	U	Y	Y	6
Yilmaz, 2008.	Y	N	Y	Y	U	U	U	Y	4
%	71	58	96	100	46	17	88	92	

1. Were the criteria for inclusion in the sample clearly defined? 2. Were the study subjects and the setting described in detail? 3. Was the exposure measured in a valid and reliable way? 4. Were objective, standard criteria used for measurement of the condition? 5. Were confounding factors identified? 6. Were strategies to deal with confounding factors stated? 7. Were the outcomes measured in a valid and reliable way? 8. Was appropriate statistical analysis used?

Table S6. Risk of bias assessment for prevalence studies

For sample size adequacy, a minimum number of three or seven participants (for studies involving participants <18 or ≥18 years of age, respectively) was set by reviewers prior to appraisal. Numbers were determined using a minimum sample size calculation based on expected prevalence values, conservatively taken from the findings of the largest study to date in this subject matter. [95,25] Unless an article scored less than 50% of the total score possible, it was deemed not at high risk of bias and was included for systematic review and meta-analysis.

Citation	Q1	Q2	Q3	Q4	Q5	Q6	Q7	Q8	Q9	Total / 9
Askew, 2017.	Y	Y	Y	Y	Y	Y	Y	Y	U	8
Burke, 1989.	Y	U	Y	N	U	Y	Y	Y	U	5
Cronly, Horgan, 2019	Y	Y	Y	Y	Y	Y	Y	Y	N	8
Driscoll, 2010.	Y	Y	Y	Y	U	Y	U	Y	U	6
Graziano, 2020.	Y	Y	Y	Y	Y	Y	Y	U	Y	7
Gundogdu, 2019.	Y	Y	Y	Y	Y	Y	Y	Y	Y	9
Hosseinzadeh, 2019.	Y	N	Y	N	Y	Y	U	U	Y	5
Knudsen, 2016.	Y	Y	Y	Y	Y	Y	Y	Y	Y	9
Kopp, 2013.	Y	U	Y	Y	N	Y	Y	Y	U	6
Latchford, 2013.	Y	Y	Y	N	U	Y	Y	U	U	5
Modi, 2011.	Y	N	Y	Y	Y	Y	U	Y	Y	7
Oliver, 2014.	Y	Y	Y	Y	Y	Y	Y	Y	Y	9
Pearson, 1991.	Y	U	Y	U	Y	Y	N	Y	U	5
Quittner, 2014.	Y	Y	Y	Y	Y	Y	N	Y	Y	8
Quon, 2015.	Y	Y	Y	Y	Y	Y	Y	Y	Y	9
Unal, 2020.	Y	Y	Y	U	Y	Y	Y	Y	N	7
Vandeleur, 2018.	Y	U	Y	U	Y	Y	U	Y	U	5
Wray, 2004.	Y	Y	Y	U	Y	Y	Y	Y	Y	8
Yohannes, 2012.	Y	U	Y	Y	Y	Y	Y	Y	Y	8
%	100	63	95	63	79	100	68	84	58	

1. Was the sample frame appropriate to address the target population? 2. Were study participants sampled in an appropriate way? 3. Was the sample size adequate? 4. Were the study subjects and the setting described in detail? 5. Was the data analysis conducted with sufficient coverage of the identified sample? 6. Were valid methods used for the identification of the condition? 7. Was the condition measured in a standard, reliable way for all participants? 8. Was there appropriate statistical analysis? 9. Was the response rate adequate, and if not, was the low response rate managed appropriately?

Table S7. Risk of bias assessment for diagnostic test accuracy studies

For sample size adequacy, a minimum number of three or seven participants (for studies involving participants <18 or ≥18 years of age, respectively) was set by reviewers prior to appraisal. Numbers were determined using a minimum sample size calculation based on expected prevalence values, conservatively taken from the findings of the largest study to date in this subject matter. [95,25] Unless an article scored less than 50% of the total score possible, it was deemed not at high risk of bias and was included for systematic review and meta-analysis.

Citation	Q1	Q2	Q3	Q4	Q5	Q6	Q7	Q8	Q9	Q10	Total / 10
Casana-Granell, 2018.	Y	Y	Y	Y	N/A	N/A	N/A	U	Y	Y	6

1. Was a consecutive or random sample of patients enrolled? 2. Was a case control design avoided? 3. Did the study avoid inappropriate exclusions? 4. Were the index test results interpreted without knowledge of the results of the reference standard? 5. If a threshold was used, was it pre-specified? 6. Is the reference standard likely to correctly classify the target condition? 7. Were the reference standard results interpreted without knowledge of the results of the index test? 8. Was there an appropriate interval between index test and reference standard? 9. Did all patients receive the same reference standard? 10. Were all patients included in the analysis?

Table S8. Risk of bias assessment for cohort studies

For sample size adequacy, a minimum number of three or seven participants (for studies involving participants <18 or ≥18 years of age, respectively) was set by reviewers prior to appraisal. Numbers were determined using a minimum sample size calculation based on expected prevalence values, conservatively taken from the findings of the largest study to date in this subject matter. [95,25] Unless an article scored less than 50% of the total score possible, it was deemed not at high risk of bias and was included for systematic review and meta-analysis.

Citation	Q1	Q2	Q3	Q4	Q5	Q6	Q7	Q8	Q9	Q10	Q11	Total / 11
Glasscoe, 2007.	Y	Y	Y	Y	Y	U	Y	Y	N	N	Y	8
Kopp, 2016.	Y	Y	Y	Y	Y	U	Y	Y	Y	N/A	Y	9
Sheehan, 2012.	N	Y	Y	U	U	U	Y	Y	Y	N	Y	6
%	67	100	100	67	67	0	100	100	67	0	100	

1. Were the two groups similar and recruited from the same population? 2. Were the exposures measured similarly to assign people to both exposed and unexposed groups? 3. Was the exposure measured in a valid and reliable way? 4. Were confounding factors identified? 5. Were strategies to deal with confounding factors stated? 6. Were the groups/participants free of the outcome at the start of the study (or at the moment of exposure)? 7. Were the outcomes measured in a valid and reliable way? 8. Was the follow up time reported and sufficient to be long enough for outcomes to occur? 9. Was follow up complete, and if not, were the reasons to loss to follow up described and explored? 10. Were strategies to address incomplete follow up utilized? 11. Was appropriate statistical analysis used?

Table S9. Risk of bias assessment for randomized controlled trial studies

For sample size adequacy, a minimum number of three or seven participants (for studies involving participants <18 or ≥18 years of age, respectively) was set by reviewers prior to appraisal. Numbers were determined using a minimum sample size calculation based on expected prevalence values, conservatively taken from the findings of the largest study to date in this subject matter. [95,25] Unless an article scored less than 50% of the total score possible, it was deemed not at high risk of bias and was included for systematic review and meta-analysis.

Citation	Q1	Q2	Q3	Q4	Q5	Q6	Q7	Q8	Q9	Q10	Q11	Q12	Q13	Total / 13
Betz, 2019.	Y	N	Y	N	N	N	Y	N	Y	Y	Y	Y	U	7
Wood, 2020.	Y	Y	U	N	Y	U	Y	Y	Y	Y	Y	Y	Y	10
%	100	50	0	0	50	0	100	50	100	100	100	100	50	

1. Was true randomization used for assignment of participants to treatment groups? 2. Was allocation to treatment groups concealed? 3. Were treatment groups similar at the baseline? 4. Were participants blind to treatment assignment? 5. Were those delivering treatment blind to treatment assignment? 6. Were outcomes assessors blind to treatment assignment? 7. Were treatment groups treated identically other than the intervention of interest? 8. Was follow up complete and if not, were differences between groups in terms of their follow up adequately described and analysed? 9. Were participants analysed in the groups to which they were randomized? 10. Were outcomes measured in the same way for treatment groups? 11. Were outcomes measured in a reliable way? 12. Was appropriate statistical analysis used? 13. Was the trial design appropriate, and any deviations from the standard RCT design (individual randomization, parallel groups) accounted for in the conduct and analysis of the trial?

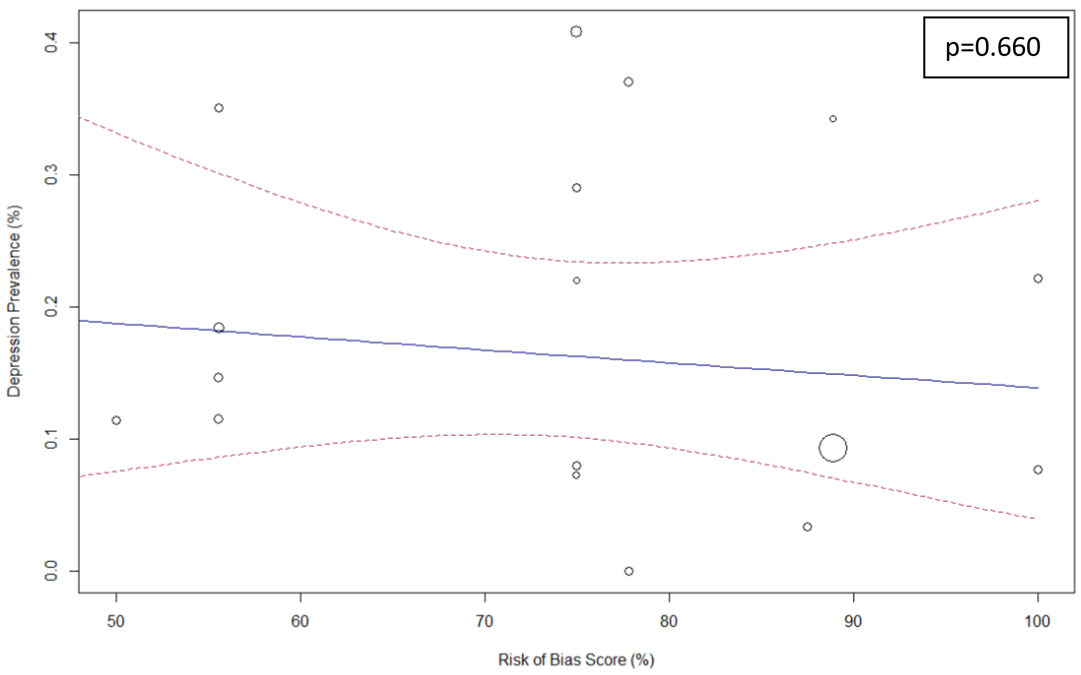

Figure S1. Meta-regression of risk of bias and depression prevalence in children and adolescents with cystic fibrosis

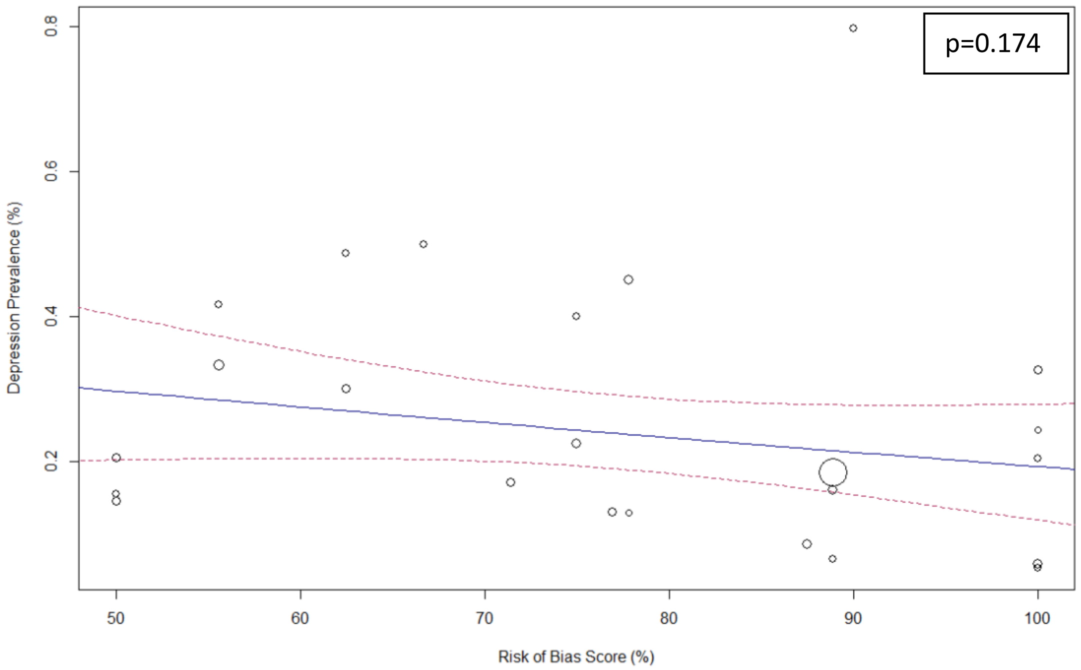

Figure S2. Meta-regression of risk of bias and depression prevalence in adults with cystic fibrosis

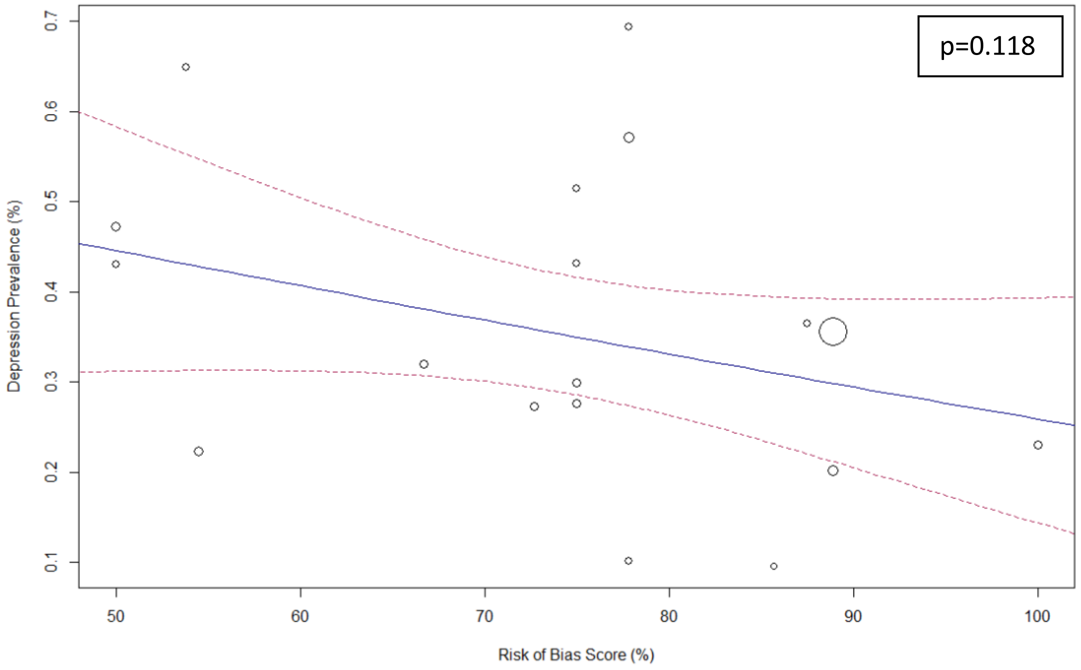

Figure S3. Meta-regression of risk of bias and depression prevalence in caregivers of people with cystic fibrosis

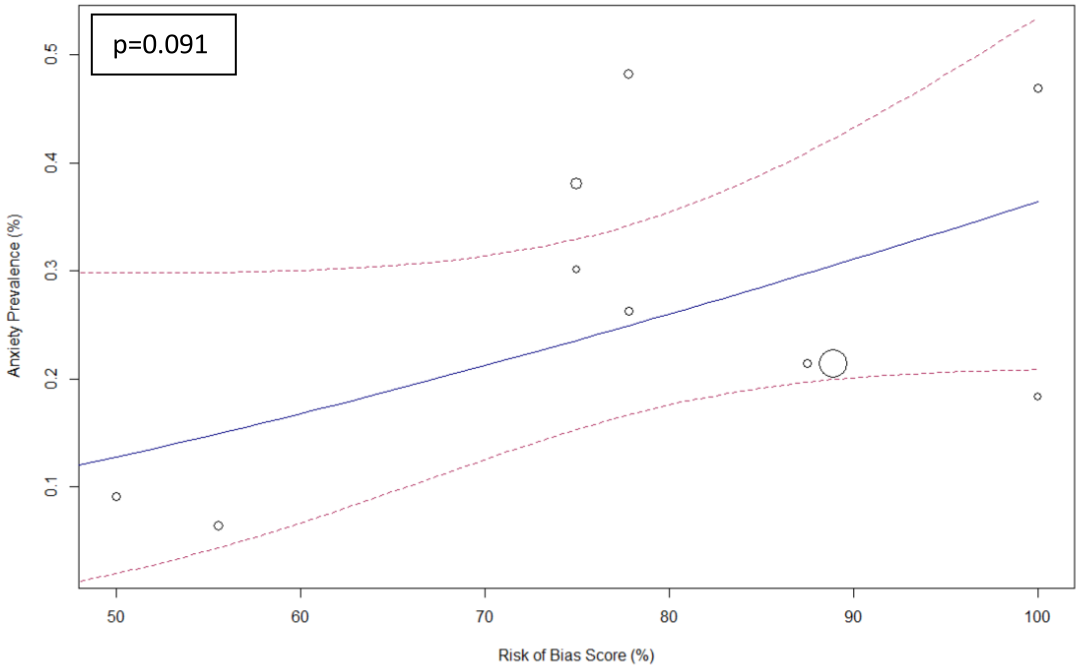

Figure S4. Meta-regression of risk of bias and anxiety prevalence in children and adolescents with cystic fibrosis

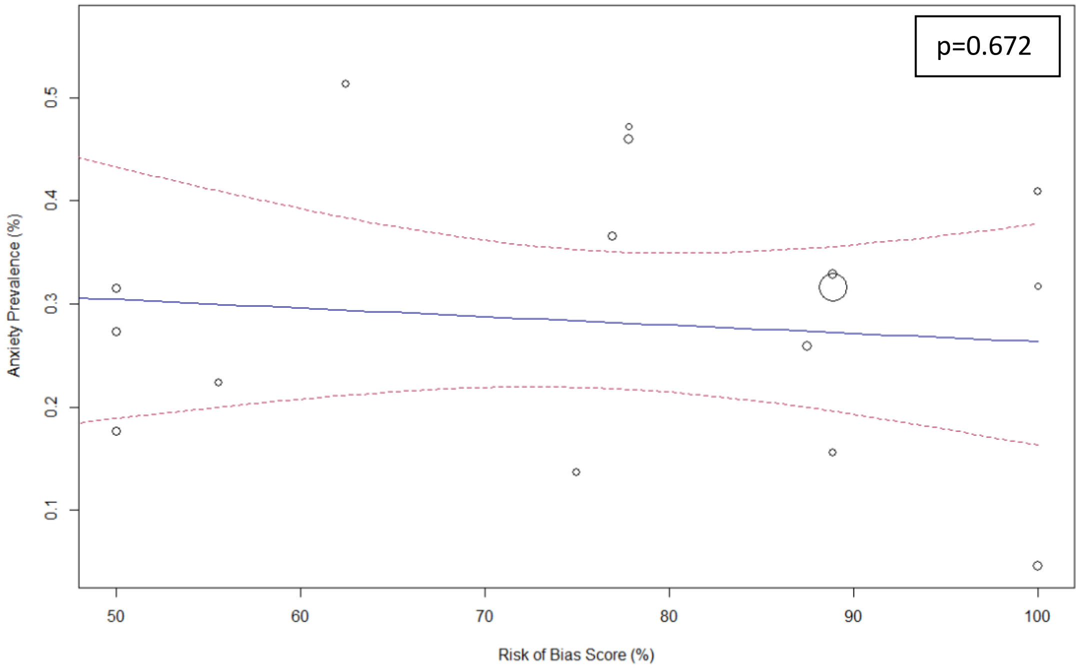

Figure S5. Meta-regression of risk of bias and anxiety prevalence in adults with cystic fibrosis

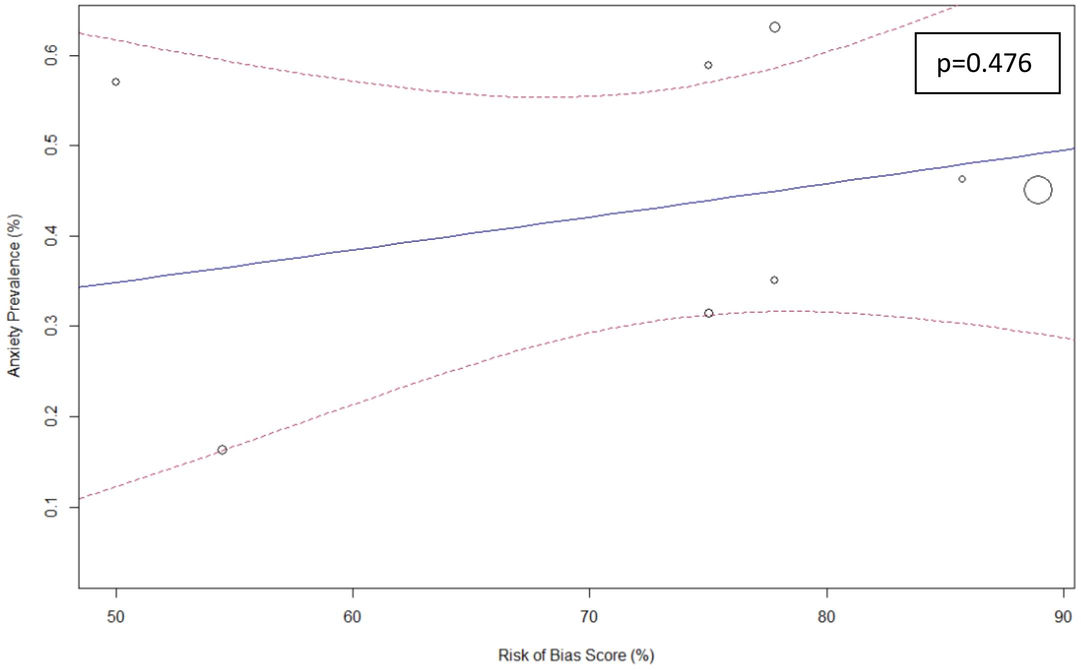

Figure S6. Meta-regression of risk of bias and anxiety prevalence in caregivers of people with cystic fibrosis

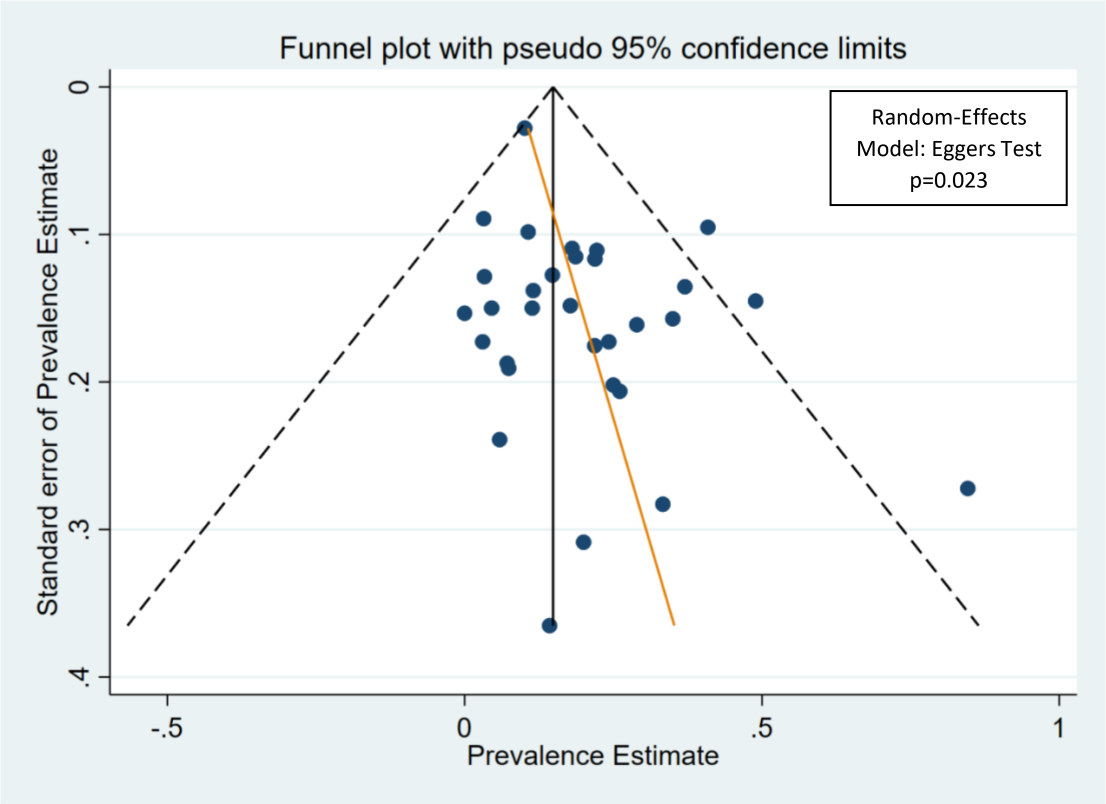

Figure S7. Depression prevalence in children and adolescents with cystic fibrosis

Prevalence estimate represented where 0.5=50%, 1=100%

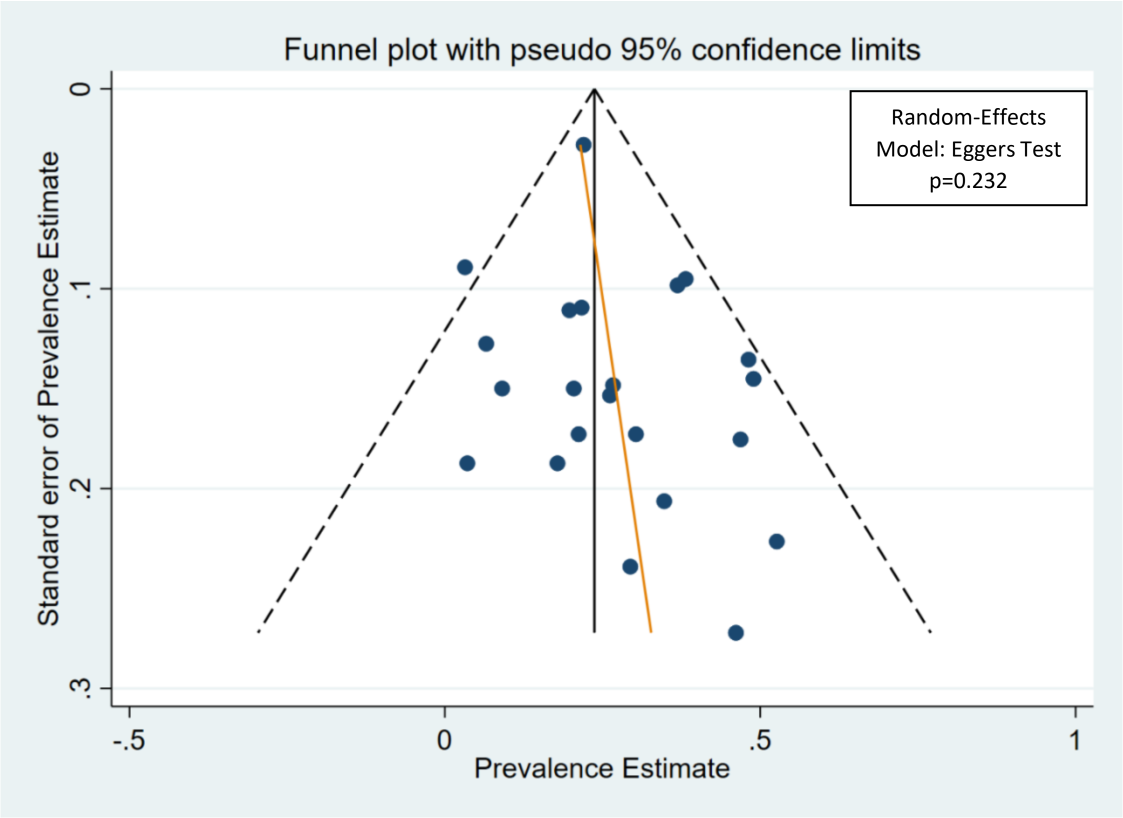

Figure S8. Anxiety prevalence in children and adolescents with cystic fibrosis

Prevalence estimate represented where 0.5=50%, 1=100%

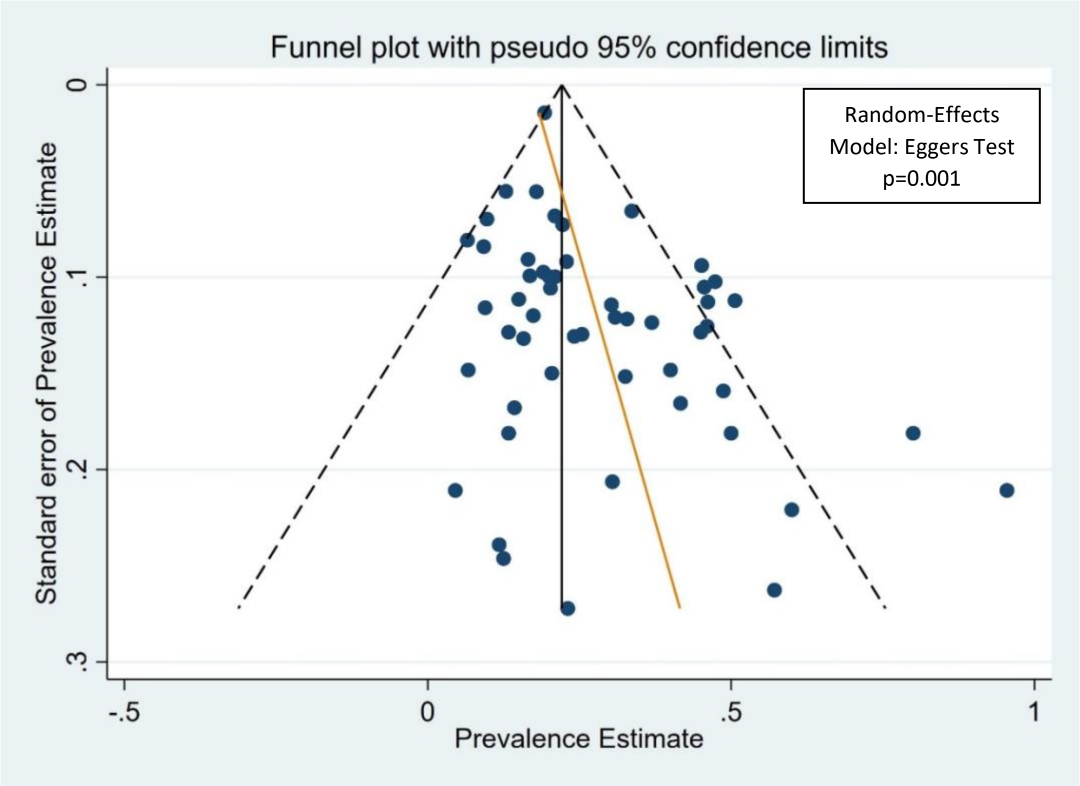

Figure S9. Depression prevalence in adults with cystic fibrosis

Prevalence estimate represented where 0.5=50%, 1=100%

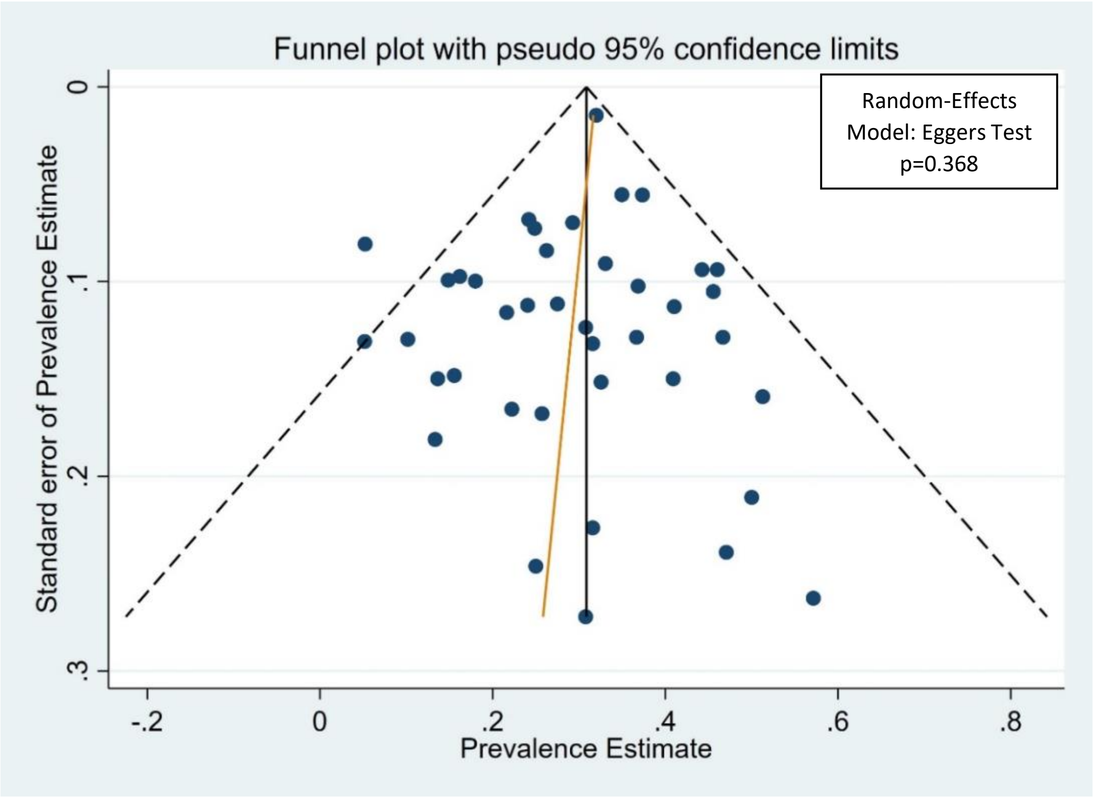

Figure S10. Anxiety prevalence in adults with cystic fibrosis

Prevalence estimate represented where 0.5=50%, 1=100%

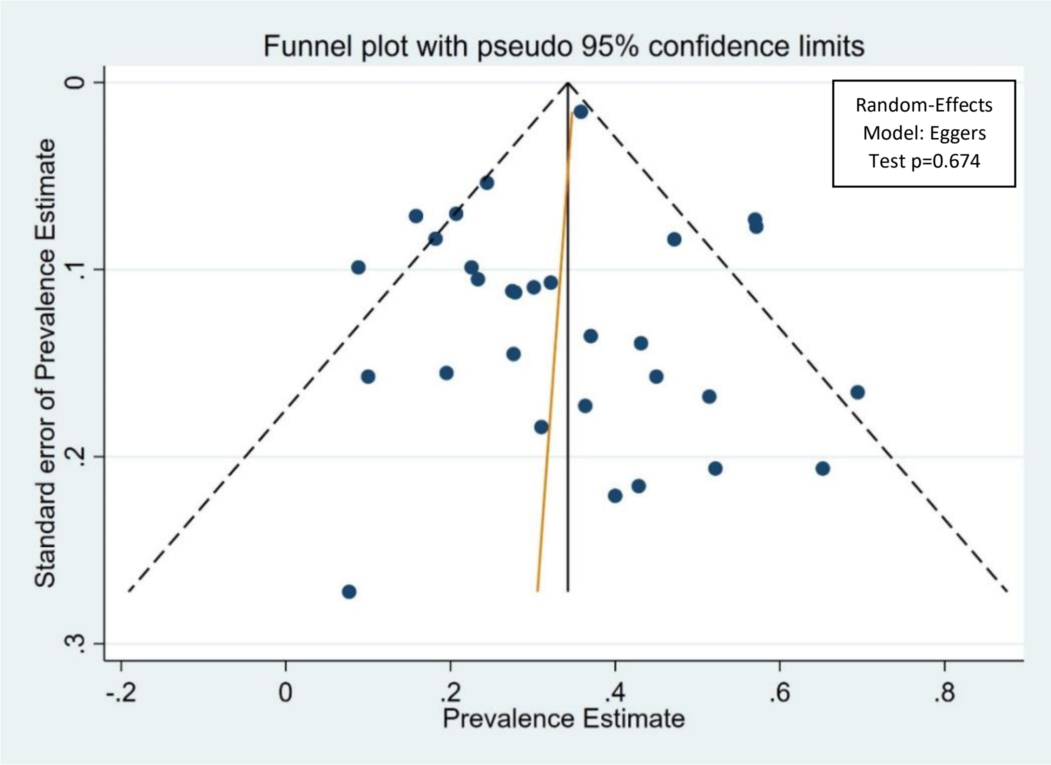

Figure S11. Depression prevalence in caregivers of people with cystic fibrosis

Prevalence estimate represented where 0.5=50%, 1=100%

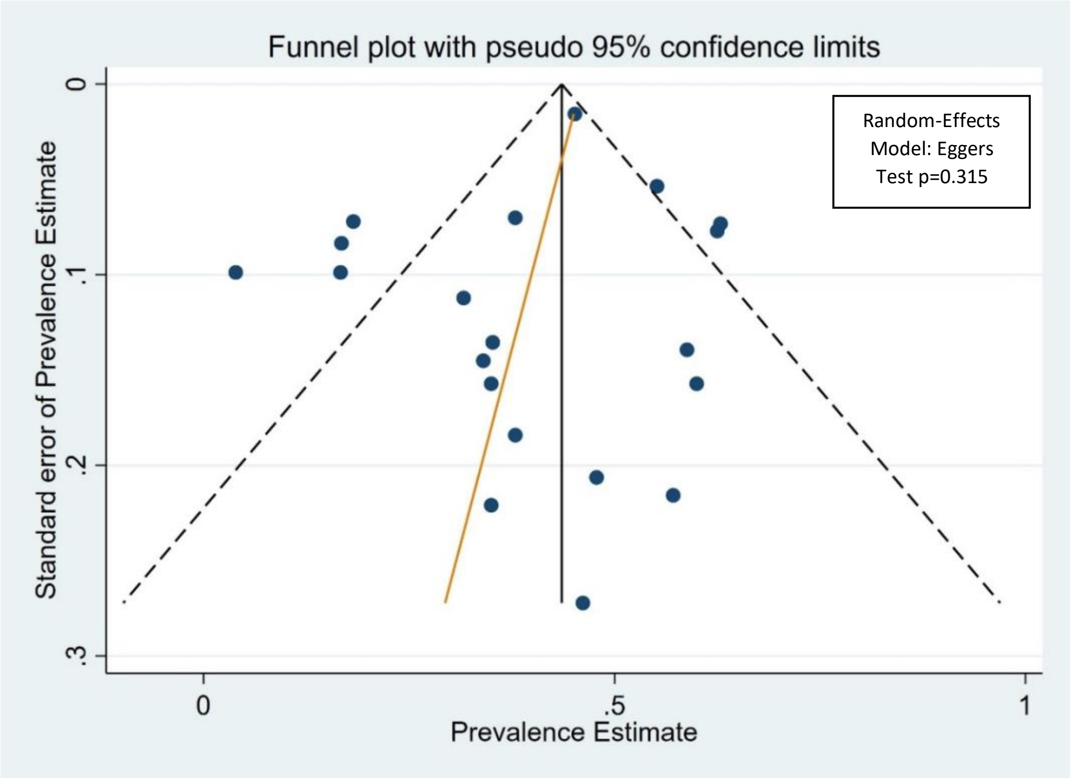

Figure S12. Anxiety prevalence in caregivers of people with cystic fibrosis

Prevalence estimate represented where 0.5=50%, 1=100%

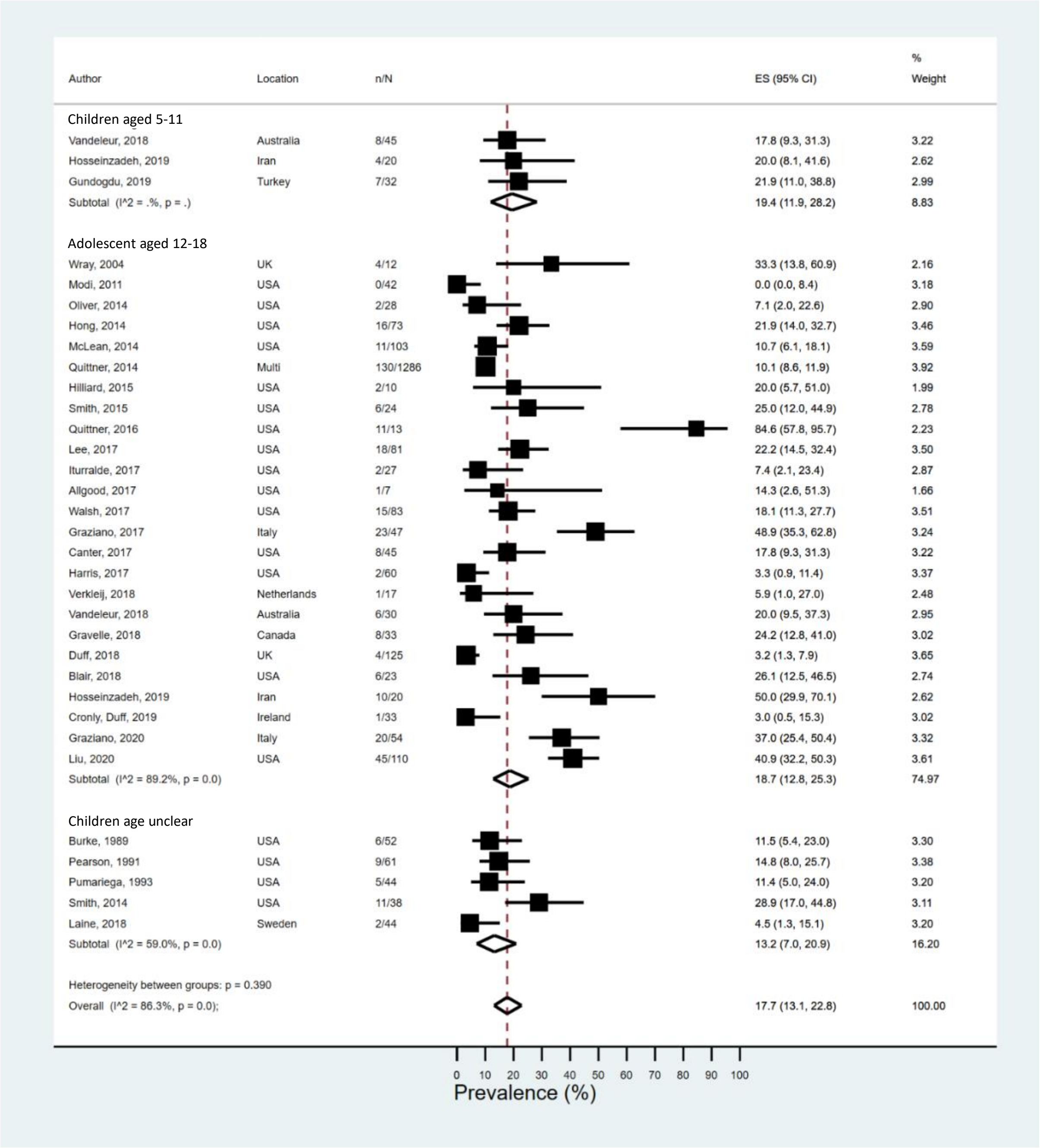

Figure S13. Forest plot of pooled prevalence of depression in children and adolescents with cystic fibrosis by age group

Abbreviations: CI: Confidence Interval; ES: Effect Size. Children age unclear refers to studies where participant age is not exclusive to either 5-11 or 12-18 years of age.

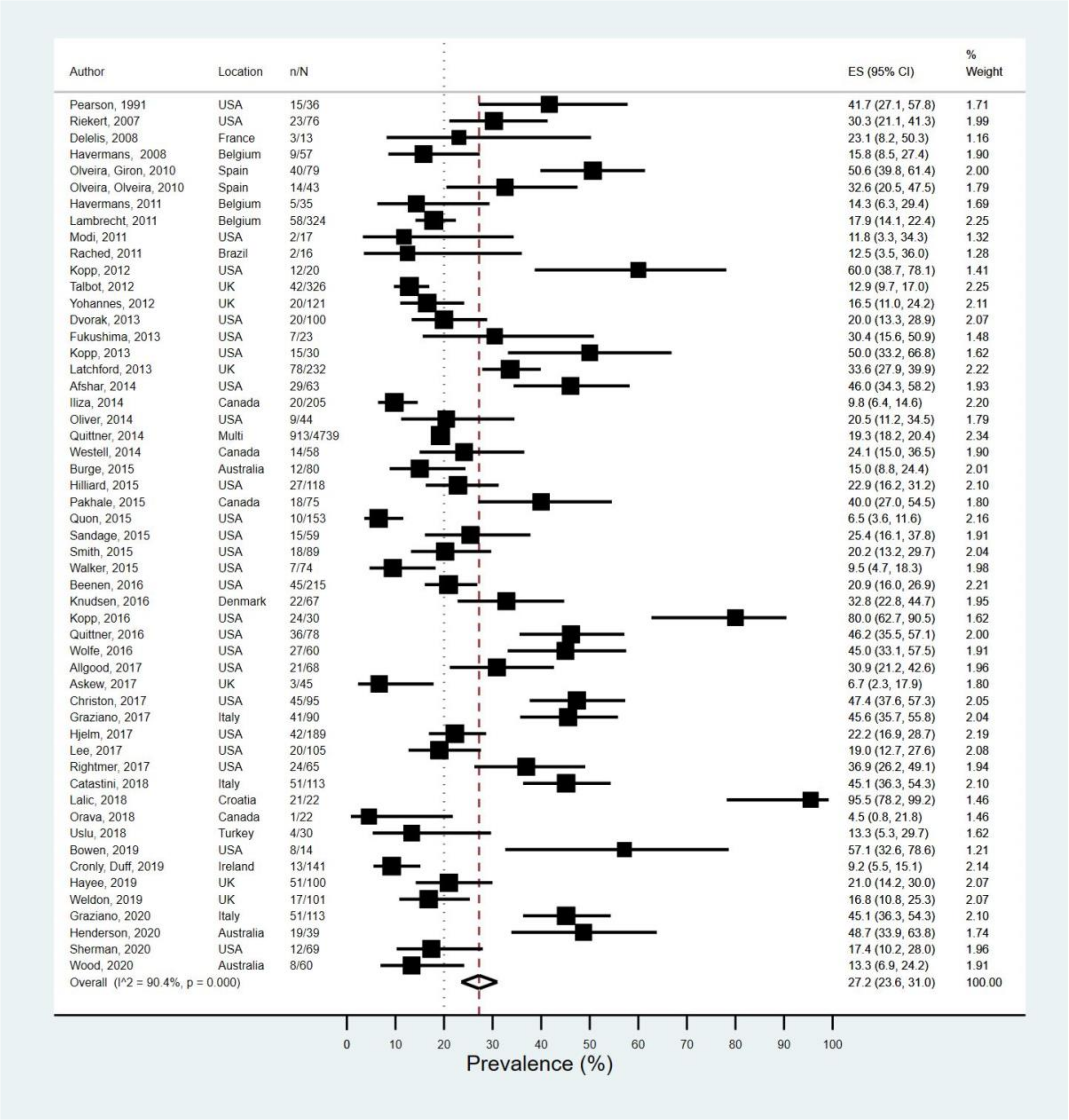

Figure S14. Forest plot of pooled prevalence of depression in adults with cystic fibrosis

Abbreviations: CI: Confidence Interval; ES: Effect Size.

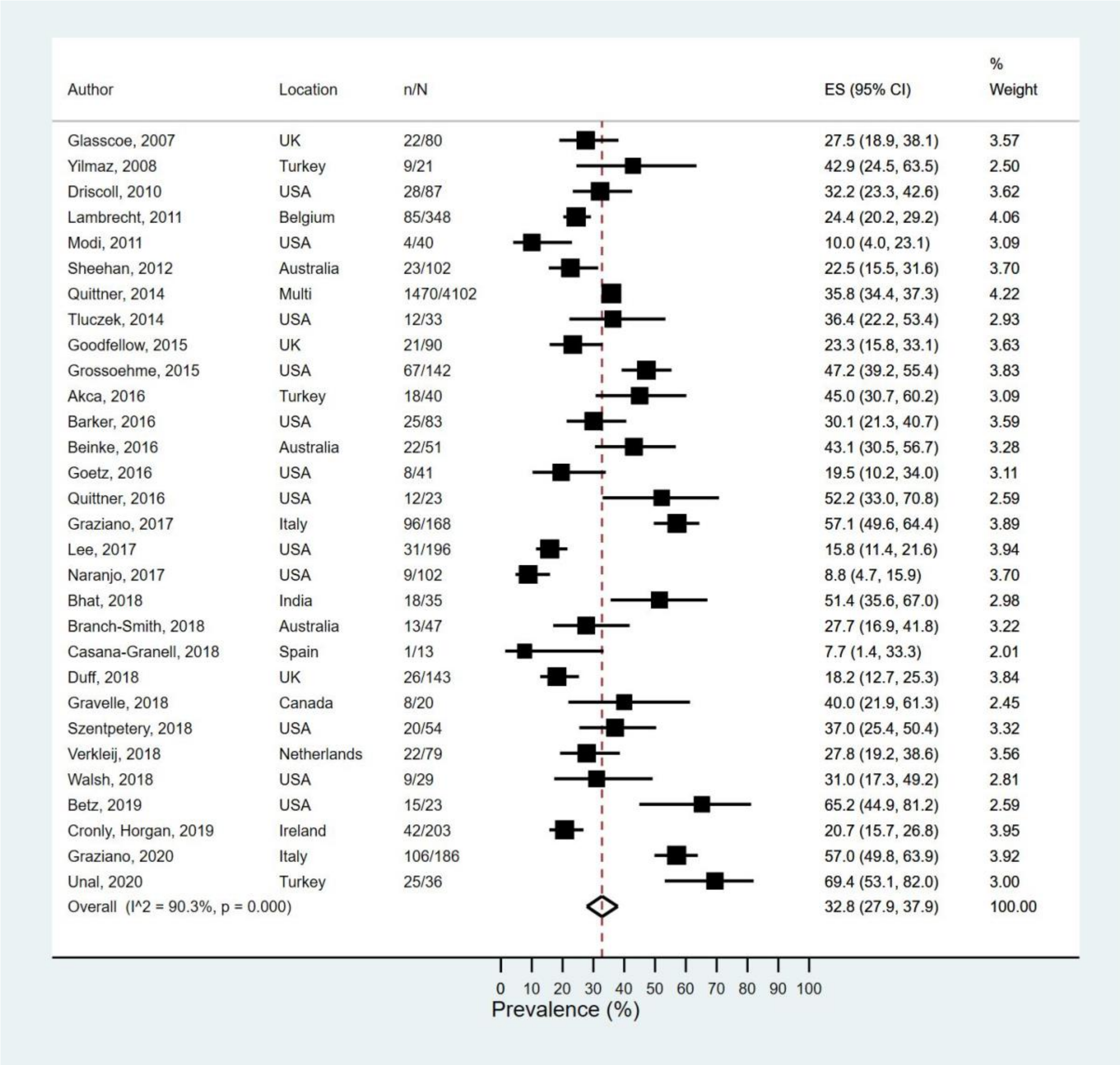

Figure S15. Forest plot of pooled prevalence of depression in caregivers of people with cystic fibrosis

Abbreviations: CI: Confidence Interval; ES: Effect Size.

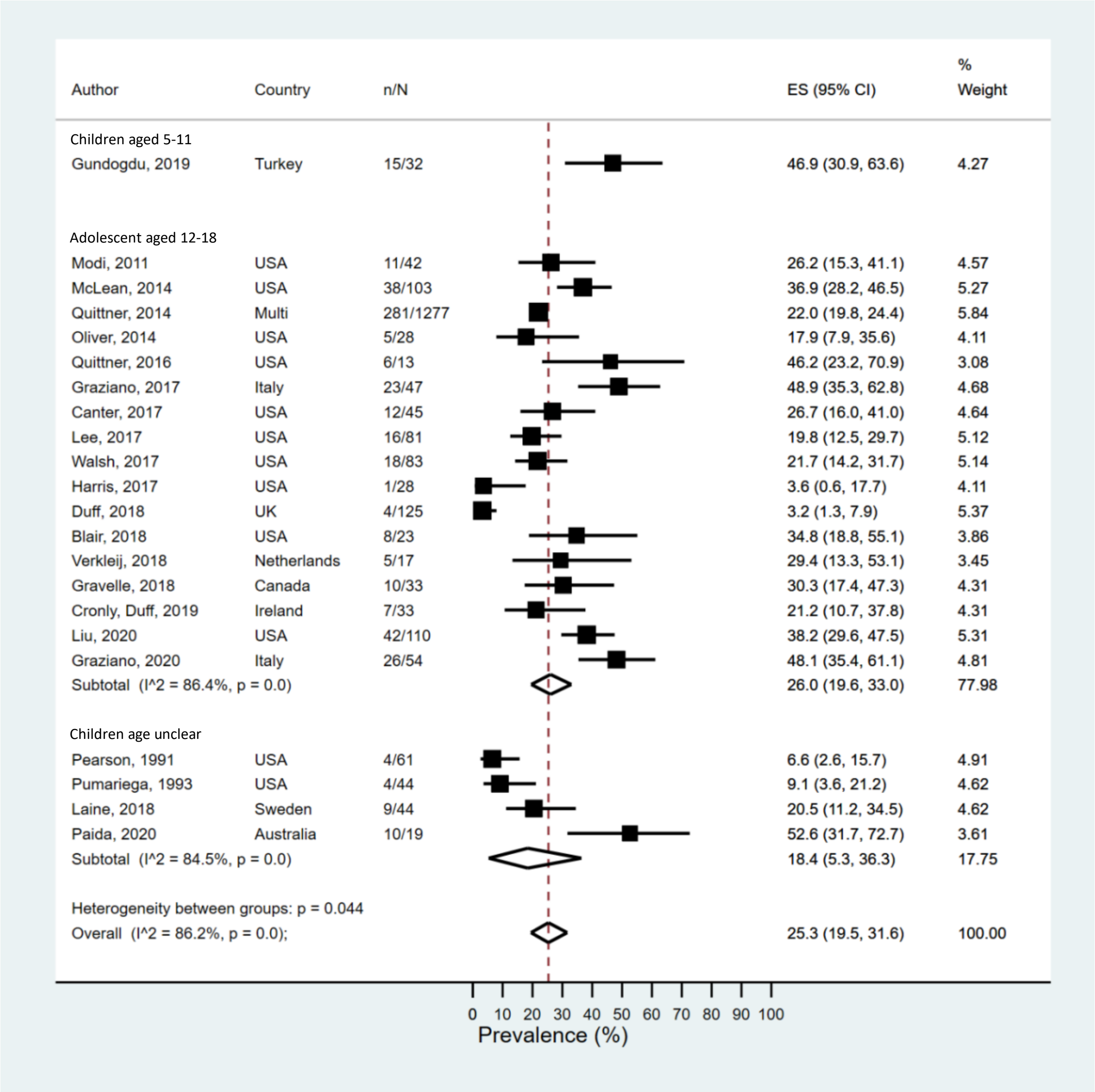

Figure S16. Forest plot of pooled prevalence of anxiety in children and adolescents with cystic fibrosis by age group

Abbreviations: CI: Confidence Interval; ES: Effect Size. Children age unclear refers to studies where participant age is not exclusive to either 5-11 or 12-18 years of age.

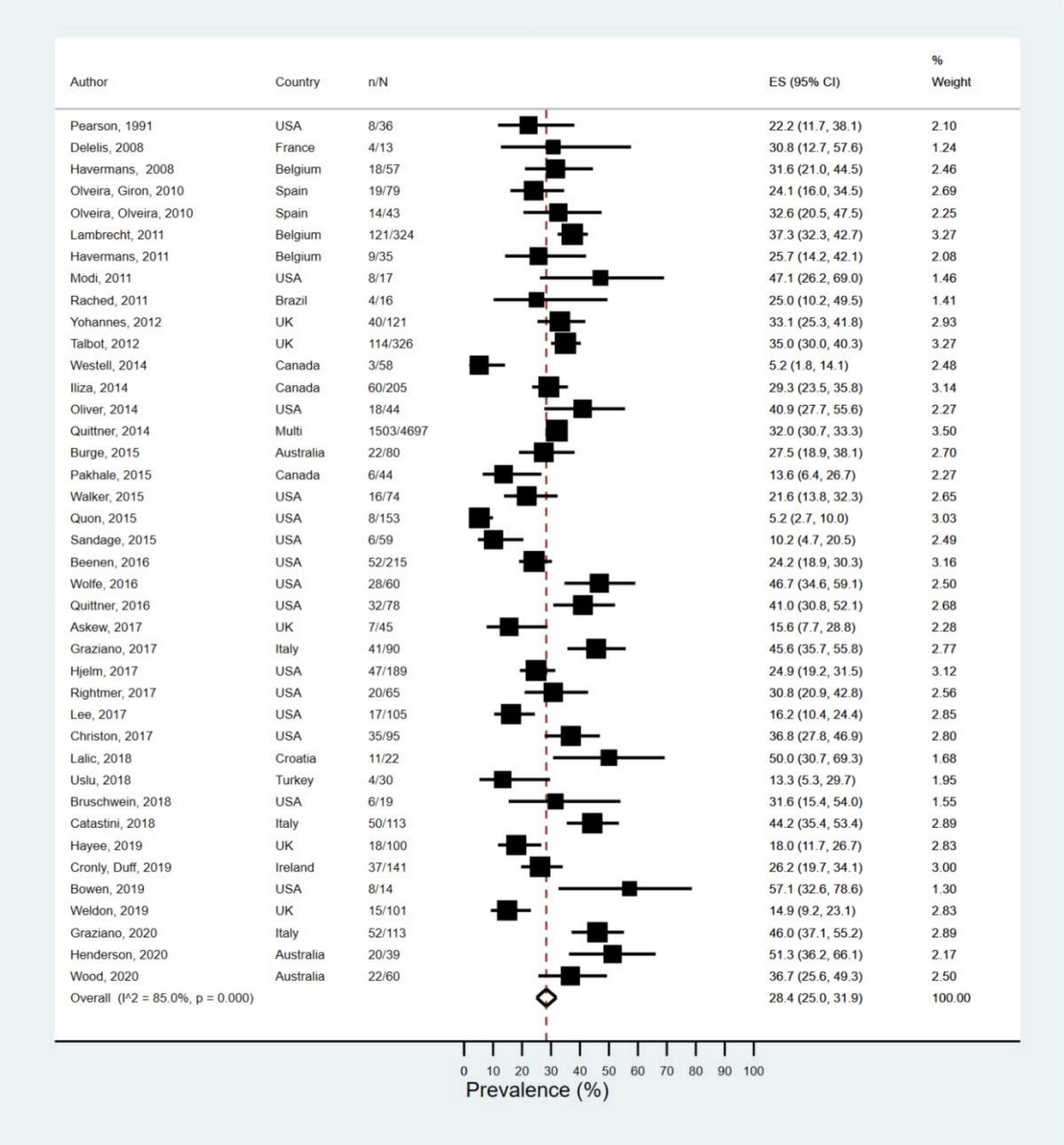

Figure S17. Forest plot of pooled prevalence of anxiety in adults with cystic fibrosis

Abbreviations: CI: Confidence Interval; ES: Effect Size.

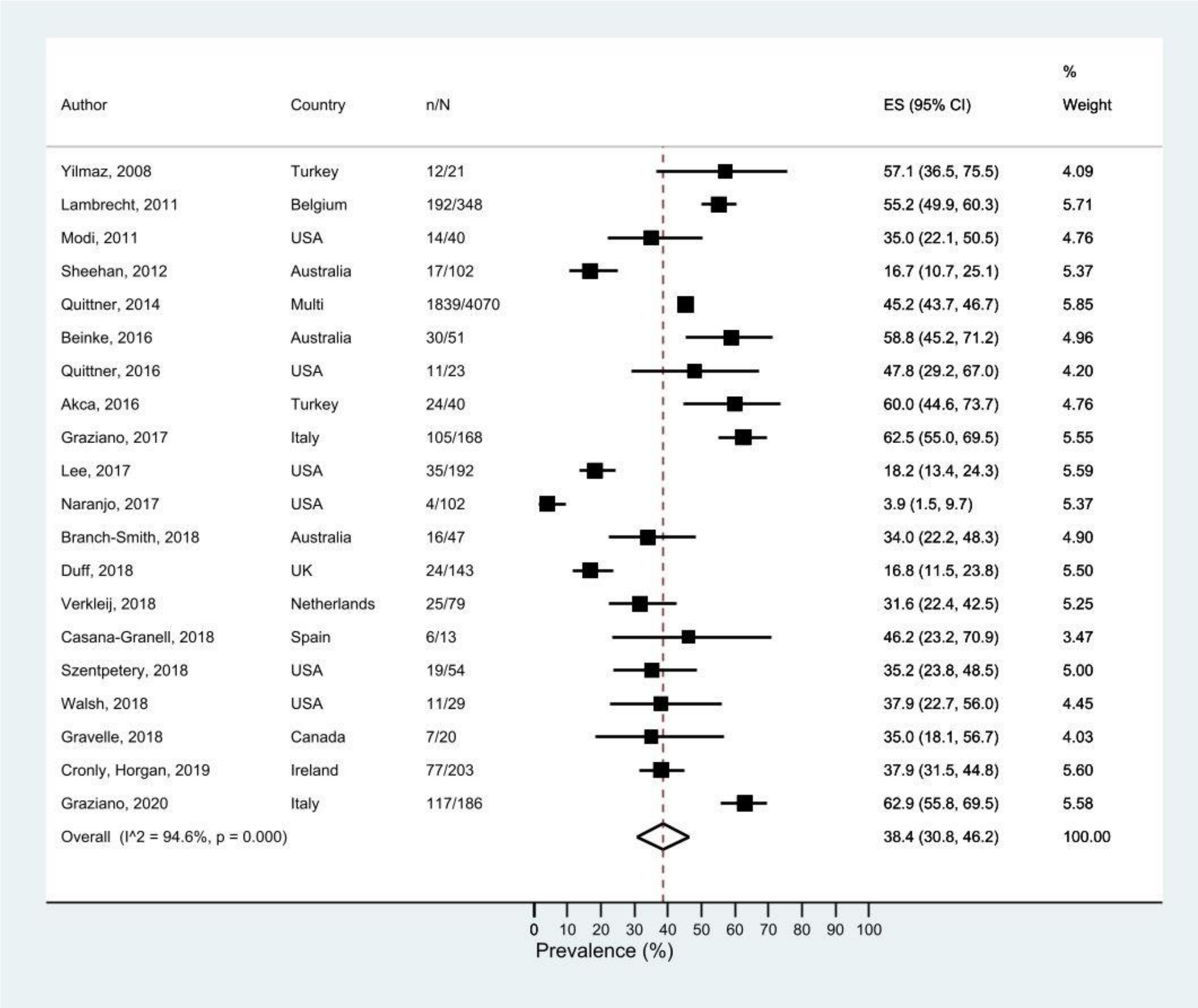

Figure S18. Forest plot of pooled prevalence of anxiety in caregivers of people with cystic fibrosis

CI: Confidence Interval; ES: Effect Size

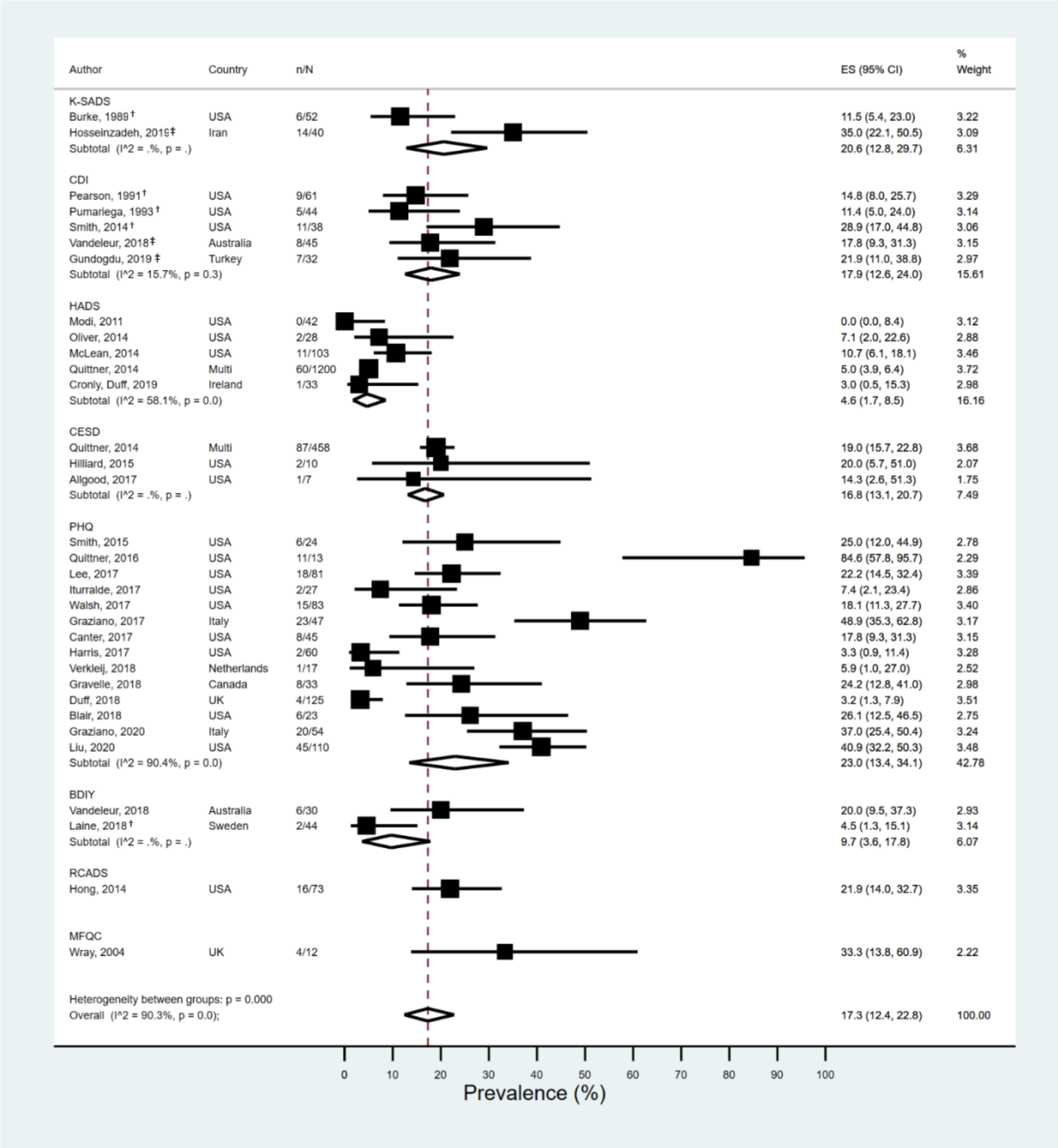

Figure S19. Forest plot of depression prevalence in children and adolescents with cystic fibrosis by psychometric tool

Abbreviations; BDIY: Beck Depression Inventory for Youth; CESD: Center for Epidemiologic Studies Depression Scale; CDI: Children's Depression Inventory; CI: Confidence Interval; ES: Effect Size; HADS: Hospital Anxiety and Depression Scale; K-SADS: Kiddie Schedule for Affective Disorders and Schizophrenia; MFQC: Mood and Feelings Questionnaire for Children; PHQ: Patient Health Questionnaire; RCADS: Revised Children's Anxiety and Depression Survey.

† Data from children with an unclear age range (where participant age is not exclusive to either 5-11 or 12-18 years of age). ‡Data from children aged 5-11 years of age.

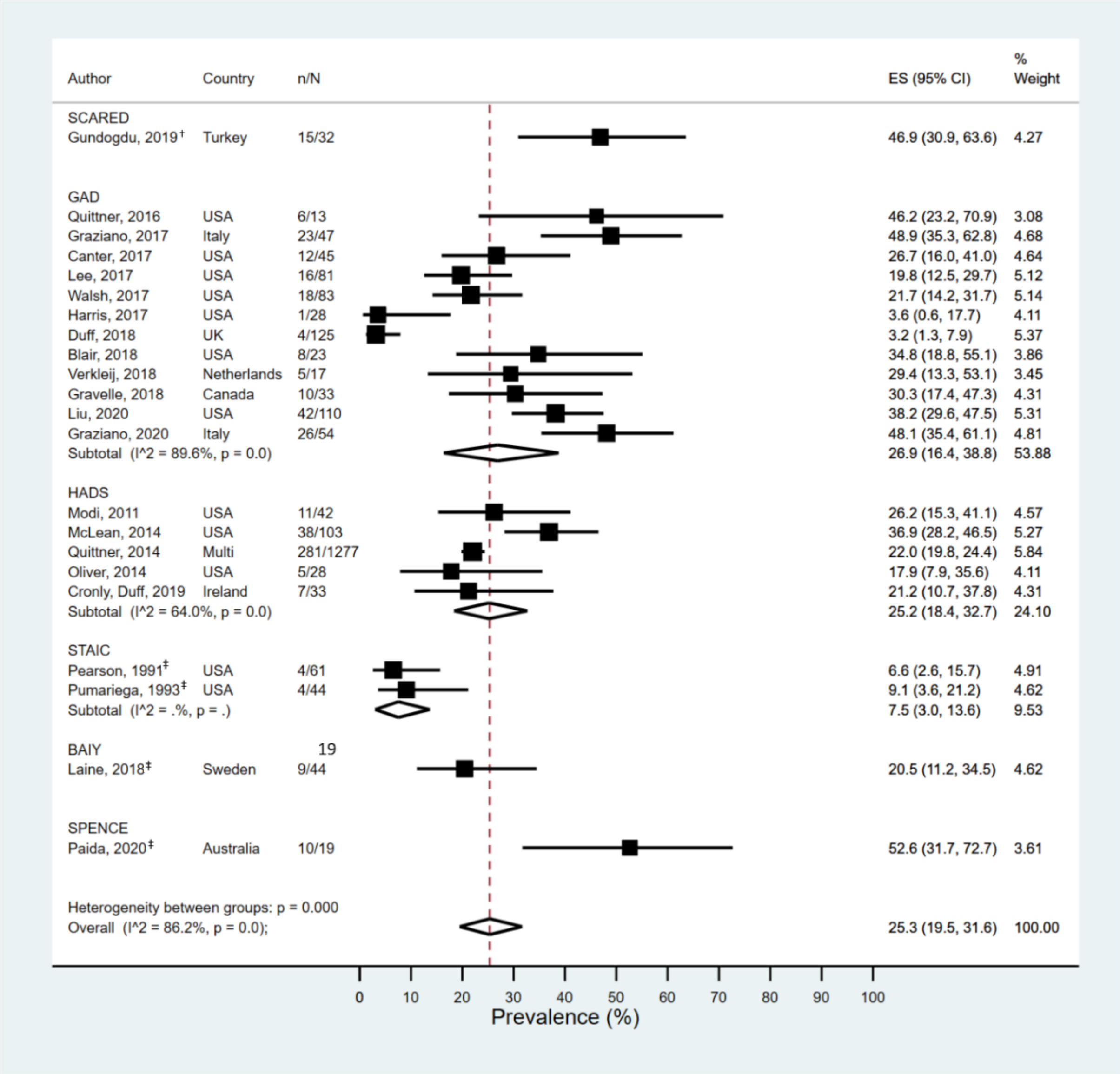

Figure S20. Forest plot of anxiety in children and adolescents with cystic fibrosis by psychometric tool

Abbreviations: BAIY: Beck Anxiety Inventory for Youth; CI: Confidence Interval; ES: Effect Size; ES: Effect Size; GAD: Generalized Anxiety Disorder 7-item measure; HADS: Hospital Anxiety and Depression Scale; SCARED: Screen for Child Anxiety Related Disorders; SPENCE: Spence Children's Anxiety Scale; STAIC: State-Trait Anxiety Inventory for Children.

†Data from children aged 5-11 years of age. ‡ Data from children with an unclear age range (where participant age is not exclusive to either 5-11 or 12-18 years of age).

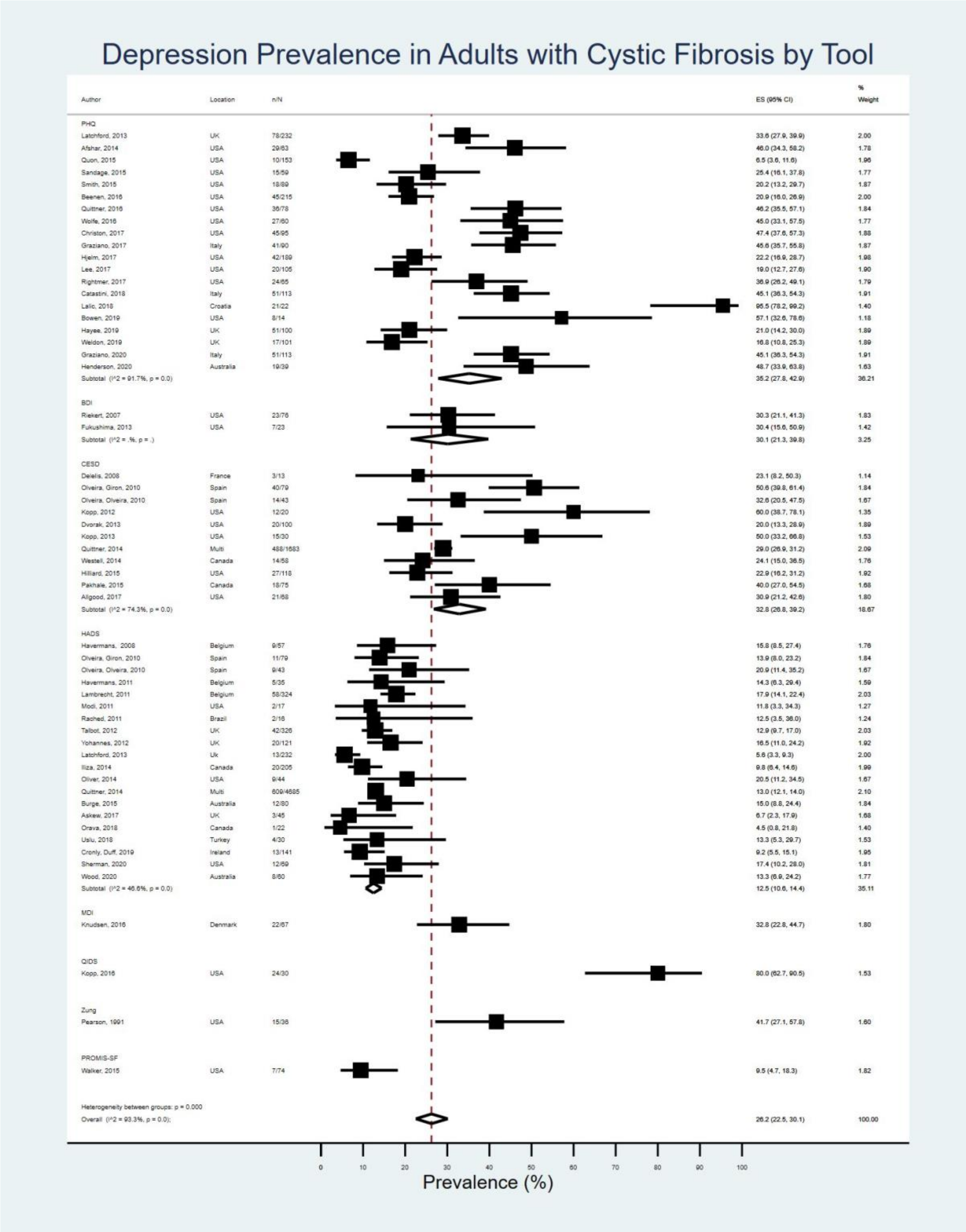

Figure S21. Forest plot of depression prevalence in adults with cystic fibrosis by psychometric tool

Abbreviations; BDI: Beck Depression Inventory; CESD: Center for Epidemiologic Studies Depression Scale; CI: Confidence Interval; ES: Effect Size; HADS: Hospital Anxiety and Depression Scale; MDI: Major Depression Inventory; PROMIS: PROMIS Depression Short Form; PHQ: Patient Health Questionnaire; QIDS: Quick Inventory of Depressive Symptomatology; Zung: Zung Self-Rating Depression Scale.

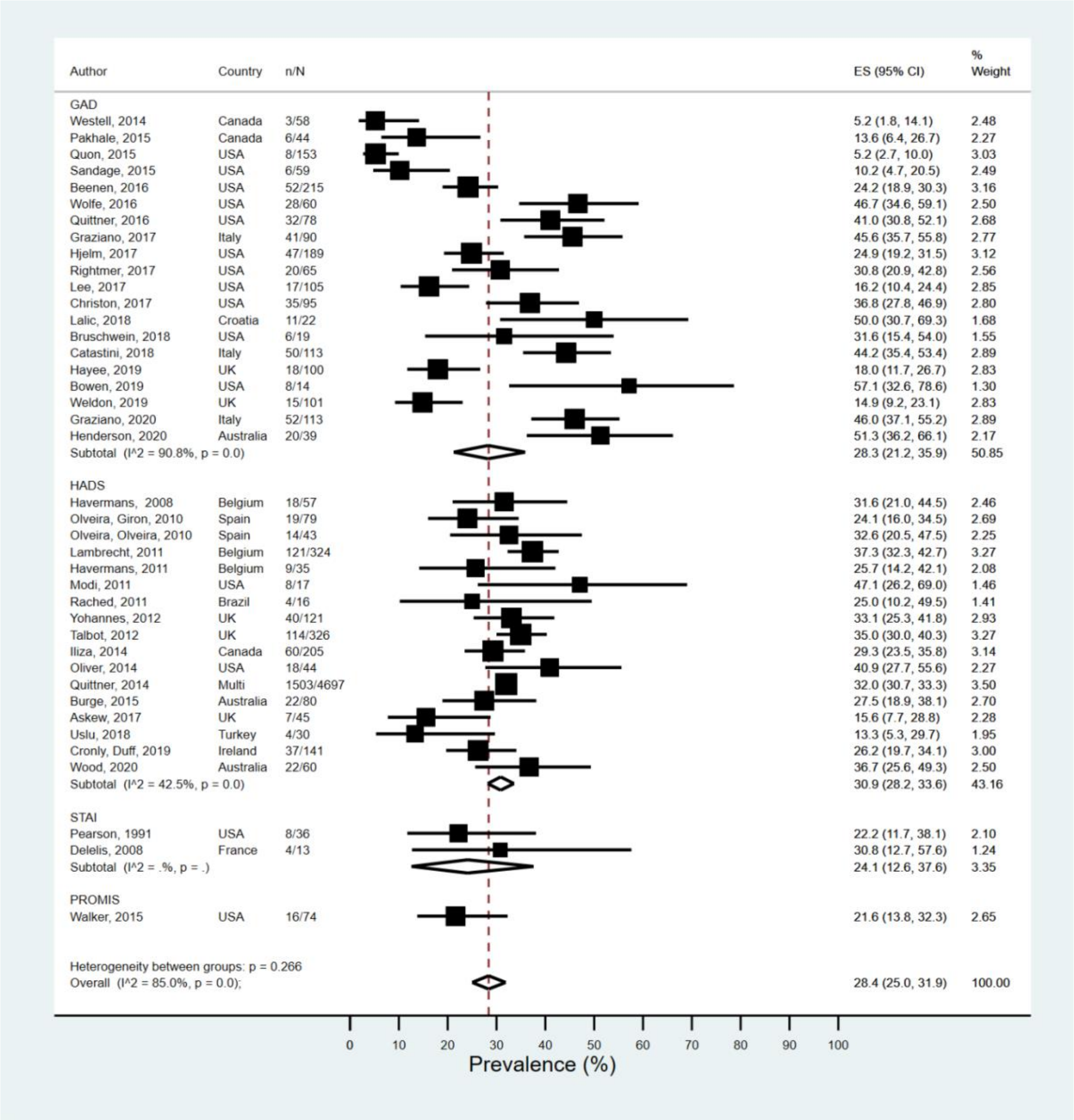

Figure S22. Forest plot of anxiety prevalence in adults with cystic fibrosis by psychometric tool

Abbreviations: CI: Confidence Interval; ES: Effect Size; GAD: Generalized Anxiety Disorder 7-item measure; HADS: Hospital Anxiety and Depression Scale; PROMIS-SF: PROMIS Anxiety Short Form; STAI: State-Trait Anxiety Inventory.

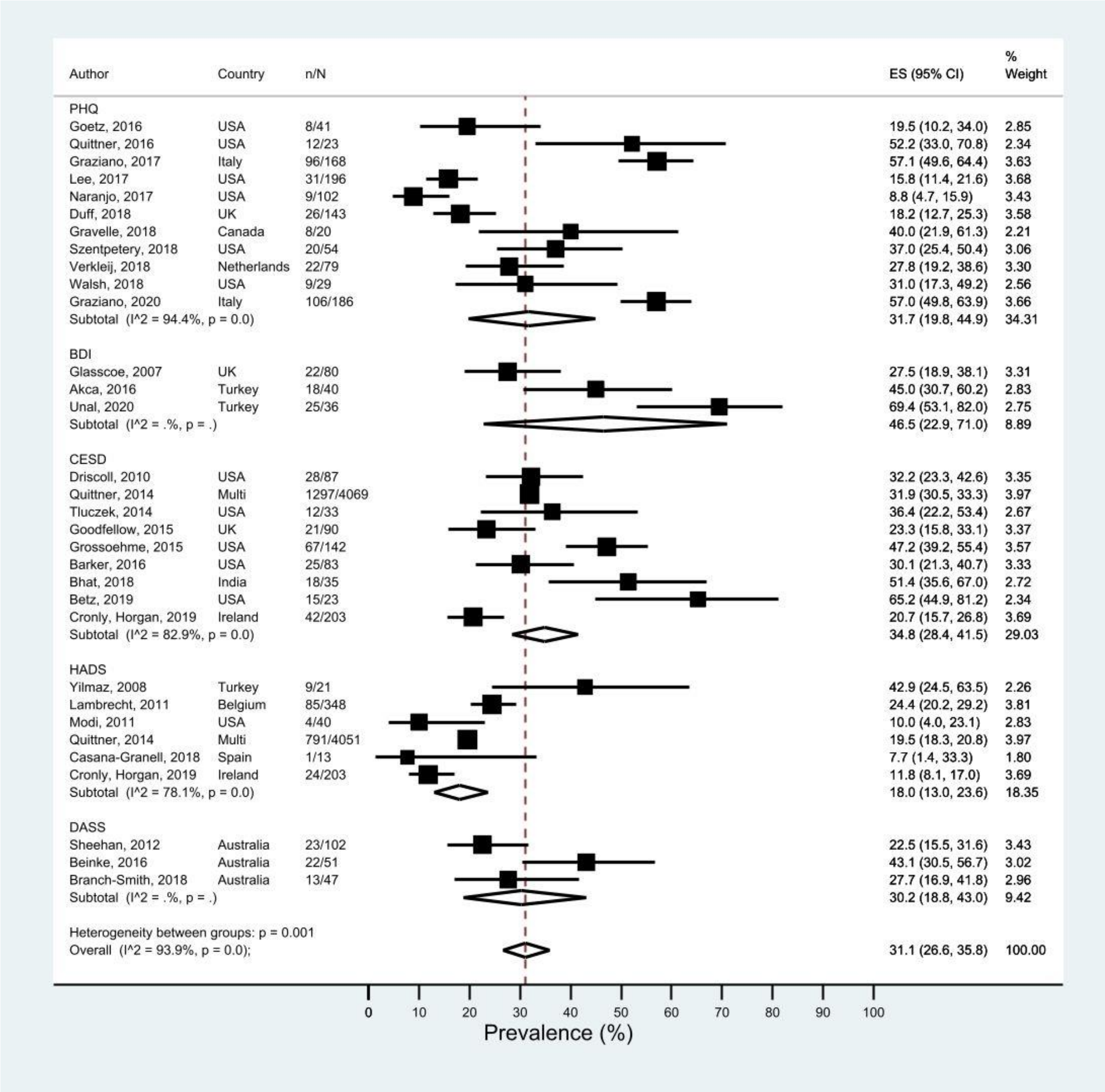

Figure S23. Forest plot of depression prevalence in caregivers of people with cystic fibrosis by psychometric tool

Abbreviations; BDI: Beck Depression Inventory; CESD: Center for Epidemiologic Studies Depression Scale; CI: Confidence Interval; DASS: Depression Anxiety Stress Scale; ES: Effect Size; HADS: Hospital Anxiety and Depression Scale; PHQ: Patient Health Questionnaire.

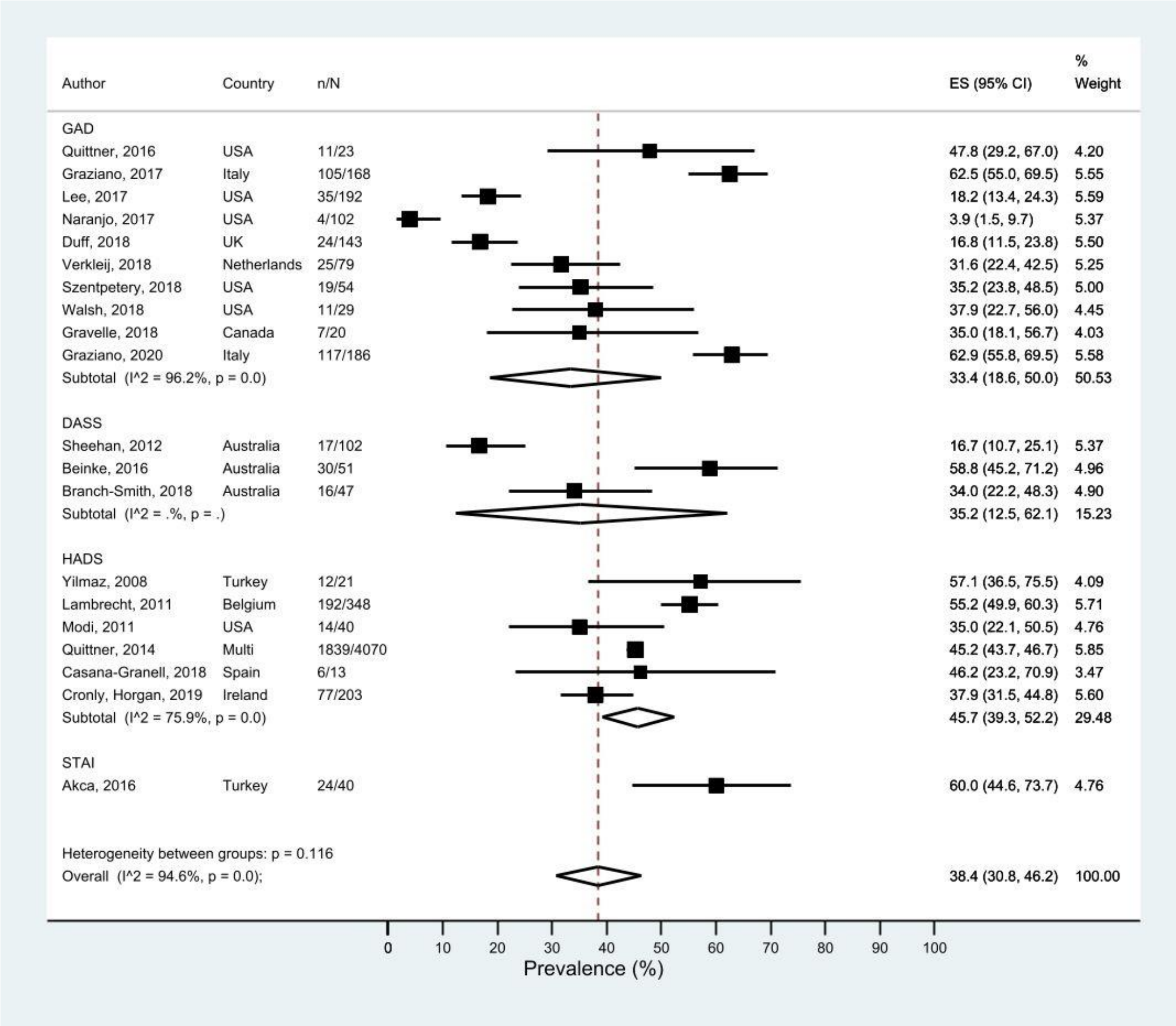

Figure S24. Forest plot of anxiety in caregivers of people with cystic fibrosis by psychometric tool

Abbreviations: CI: Confidence Interval; DASS: Depression Anxiety Stress Scale; ES: Effect Size; GAD: Generalized Anxiety Disorder 7-item measure; HADS: Hospital Anxiety and Depression Scale; STAI: State-Trait Anxiety Inventory.

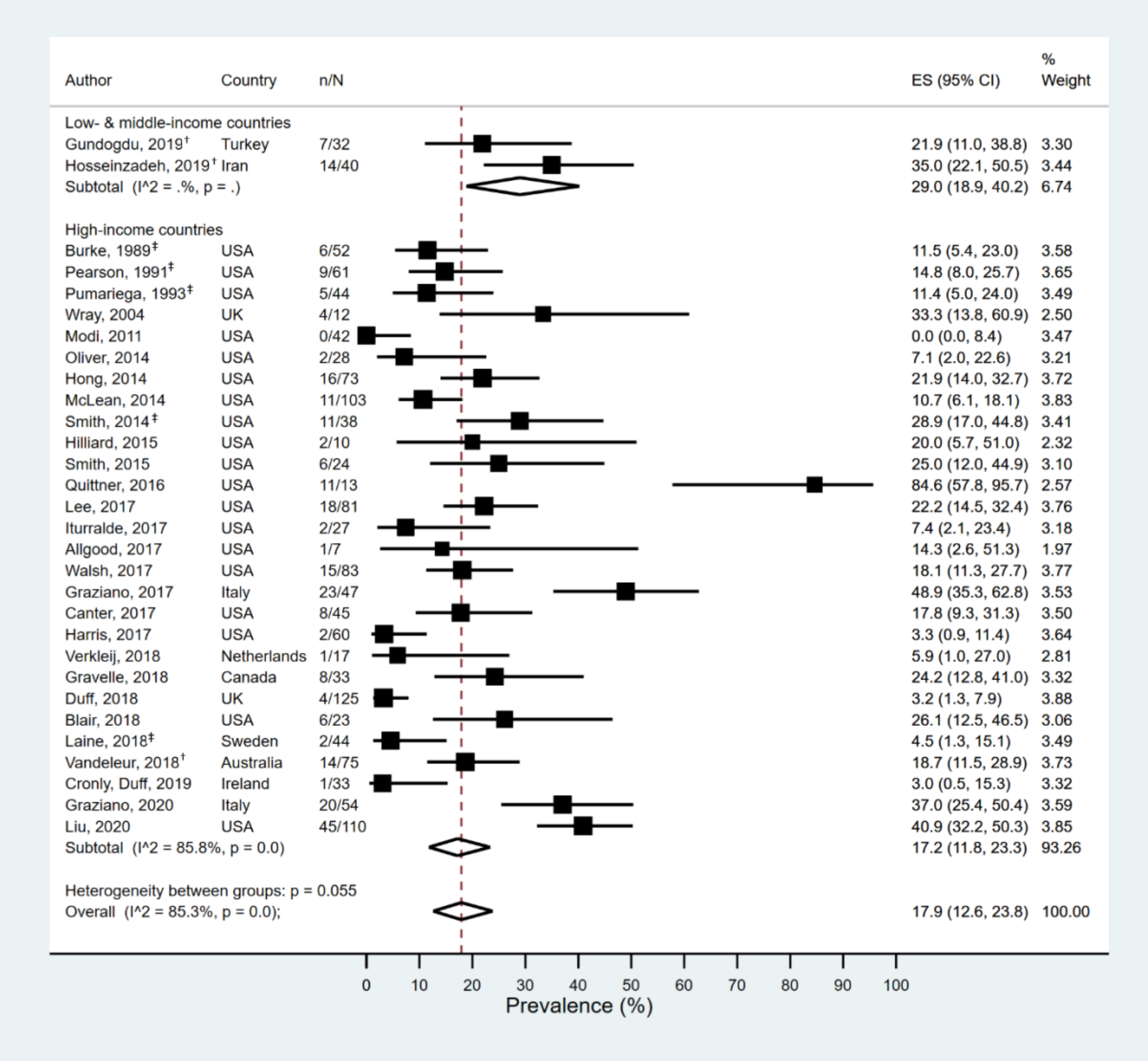

Figure S25. Forest plot of depression in children and adolescents with cystic fibrosis by study location

Abbreviations: CI: Confidence Interval; ES: Effect Size.

†Data from children aged 5-11 years of age. ‡ Data from participants with an unclear age range (where participant age is not exclusive to either 5-11 or 12-18 years of age).

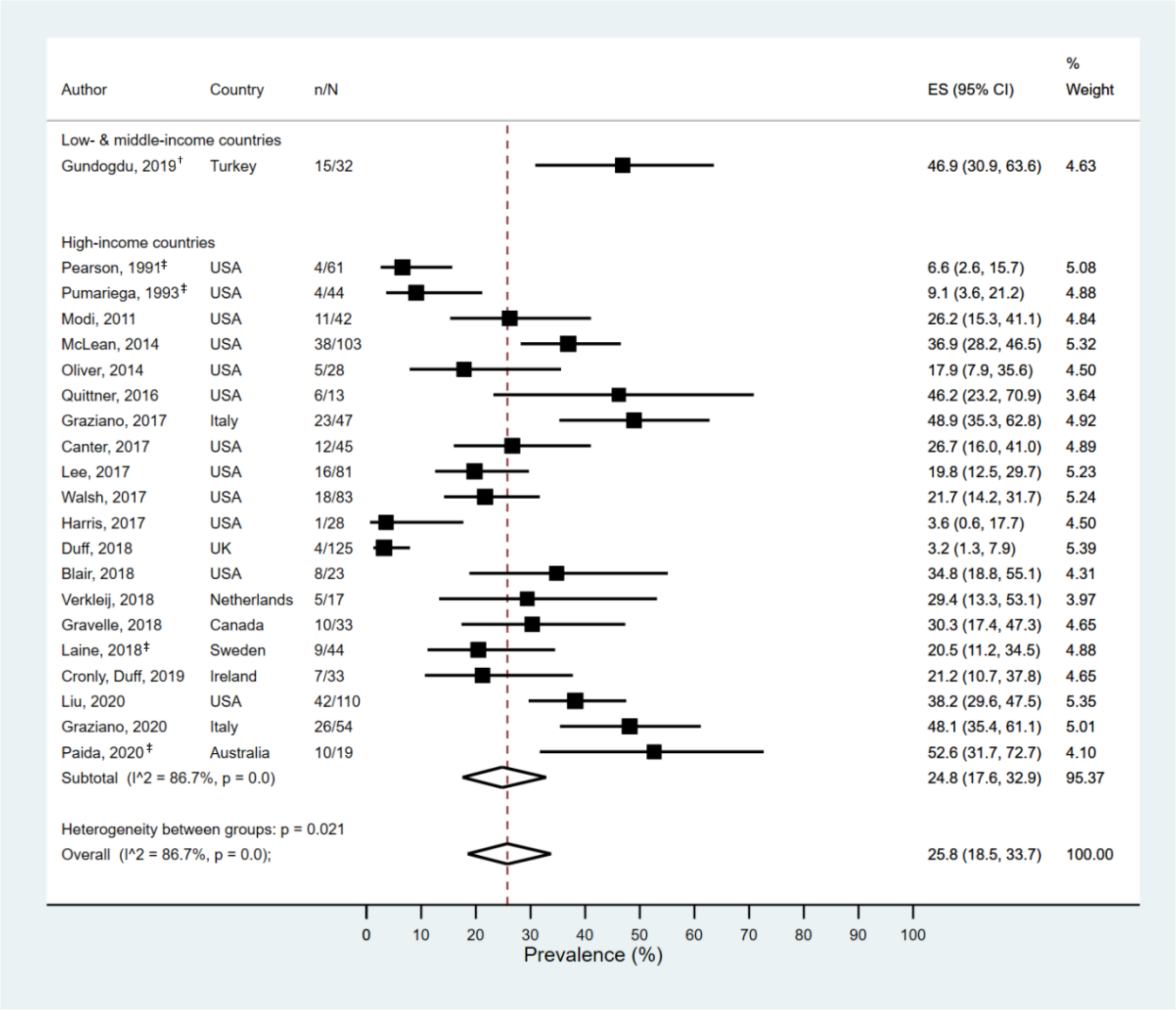

Figure S26. Forest plot of anxiety in children and adolescents with cystic fibrosis by study location

Abbreviations: CI: Confidence Interval; ES: Effect Size.
†Data from children aged 5-11 years of age. ‡ Data from participants with an unclear age range (where participant age is not exclusive to either 5-11 or 12-18 years of age).

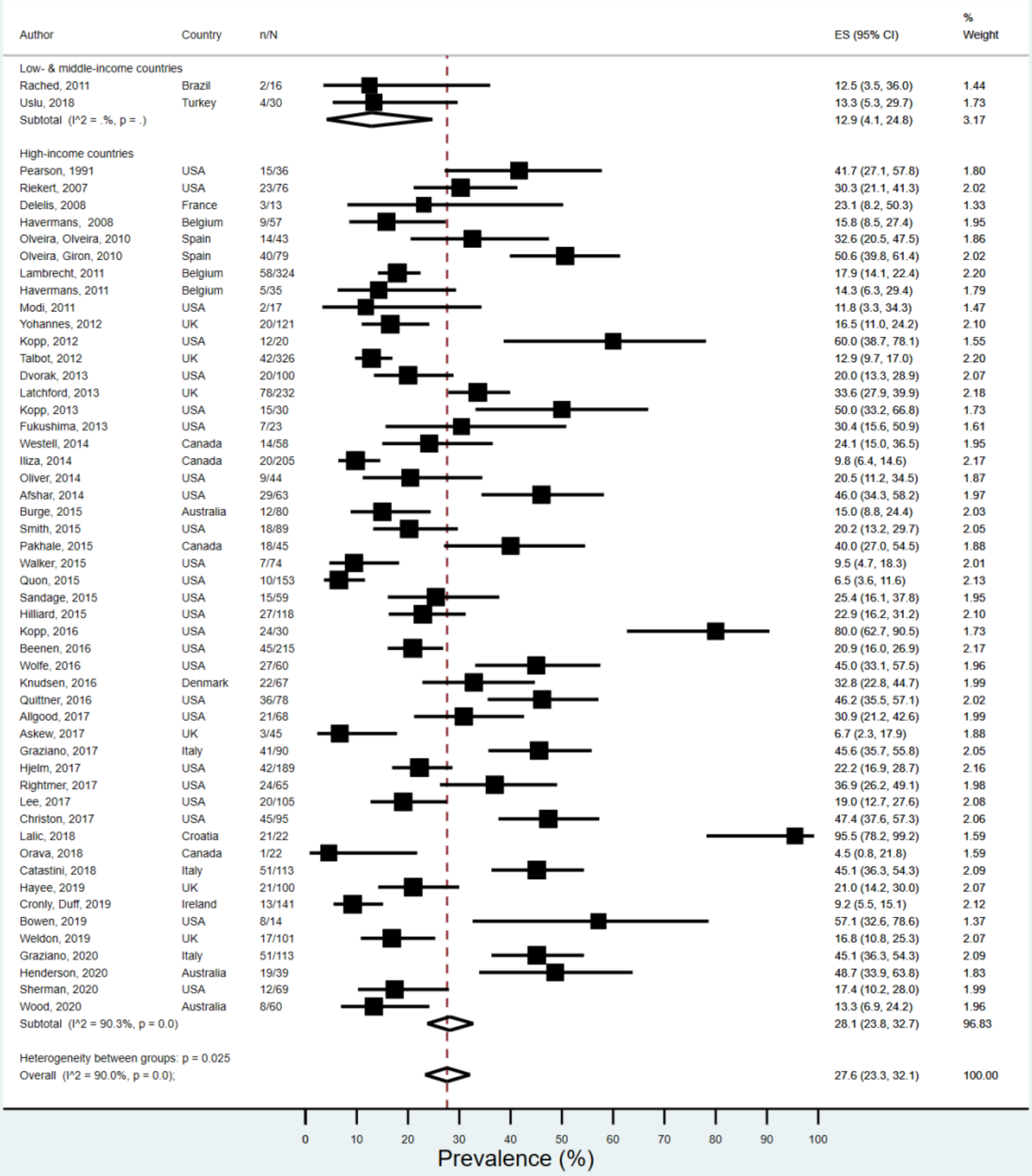

Figure S27. Forest plot of depression in adults with cystic fibrosis by study location

Abbreviations: CI: Confidence Interval; ES: Effect Size.

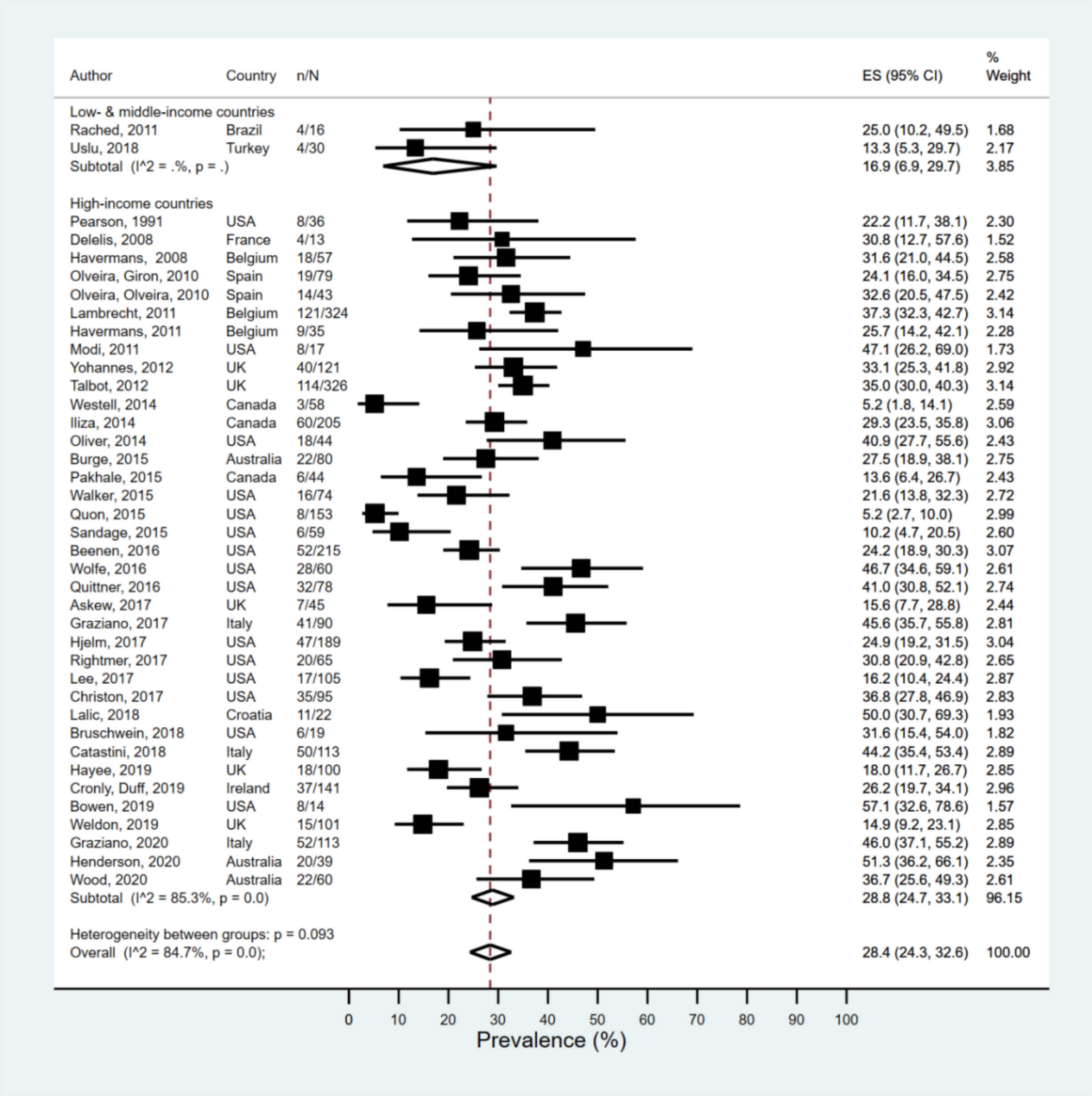

Figure S28. Forest plot of anxiety in adults with cystic fibrosis by study location

Abbreviations: CI: Confidence Interval; ES: Effect Size.

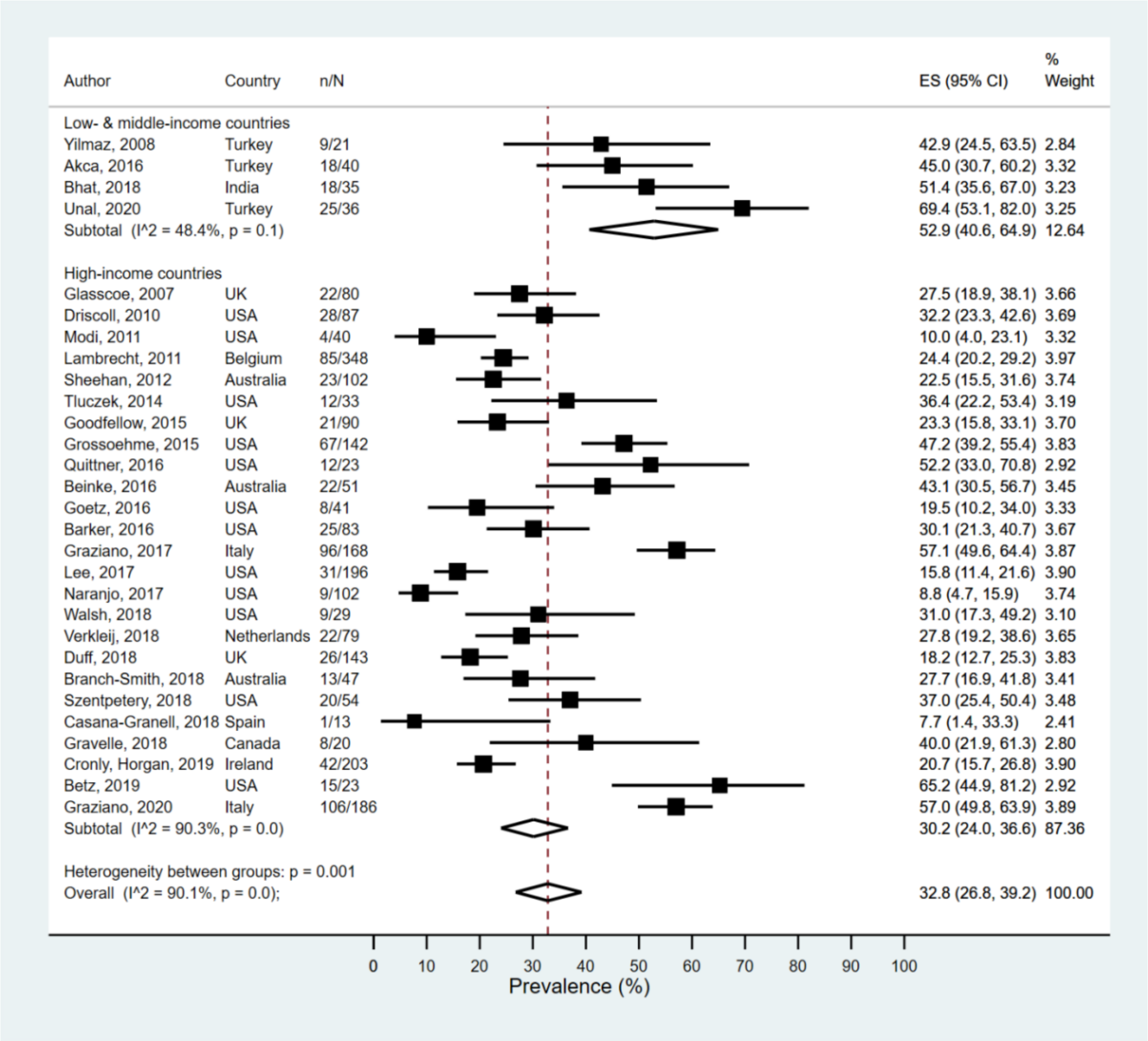

Figure S29. Forest plot of depression in caregivers of people with cystic fibrosis by study location

Abbreviations: CI: Confidence Interval; ES: Effect Size.

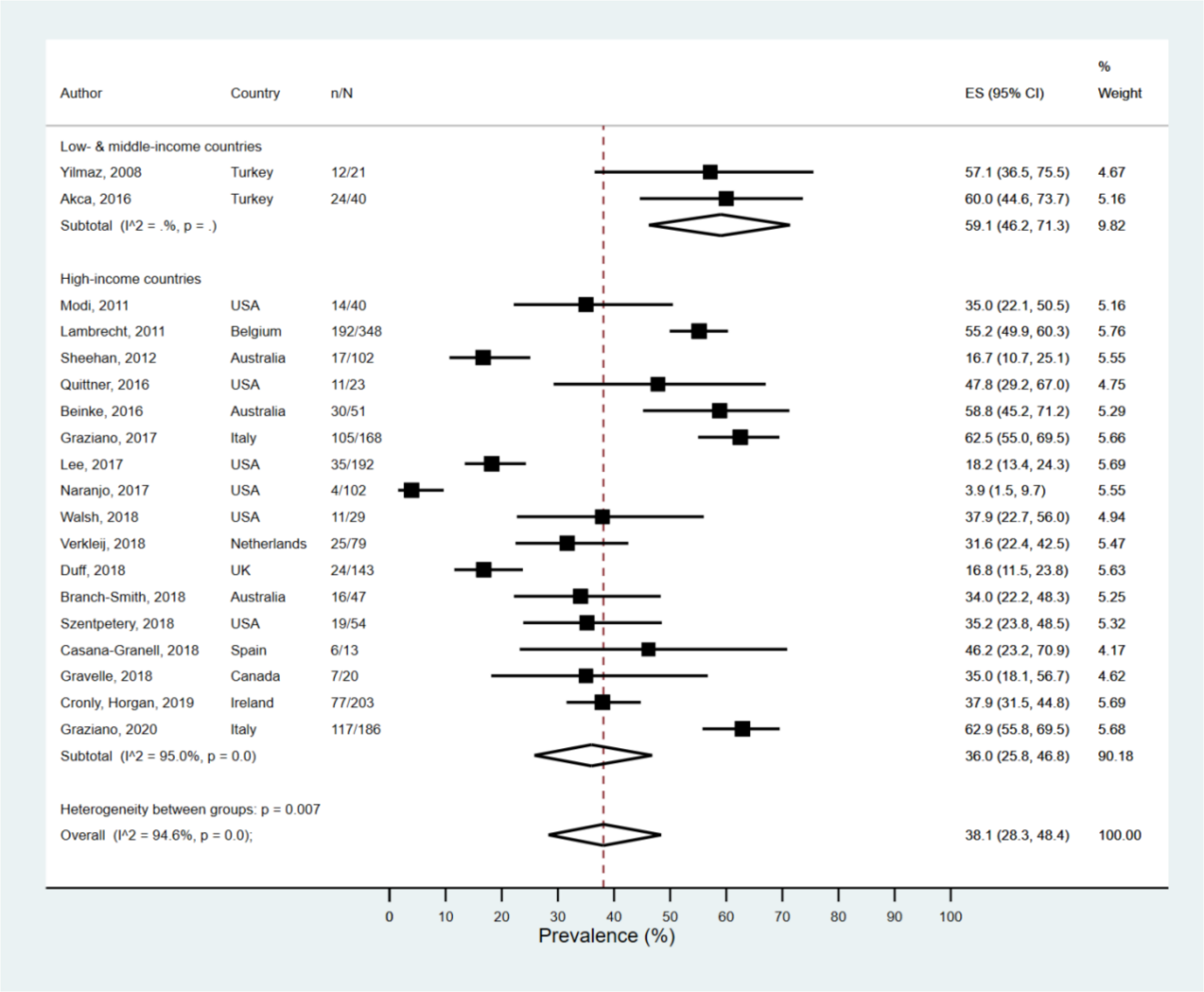

Figure S30. Forest plot of anxiety in caregivers of people with cystic fibrosis by study location

Abbreviations: CI: Confidence Interval; ES: Effect Size.

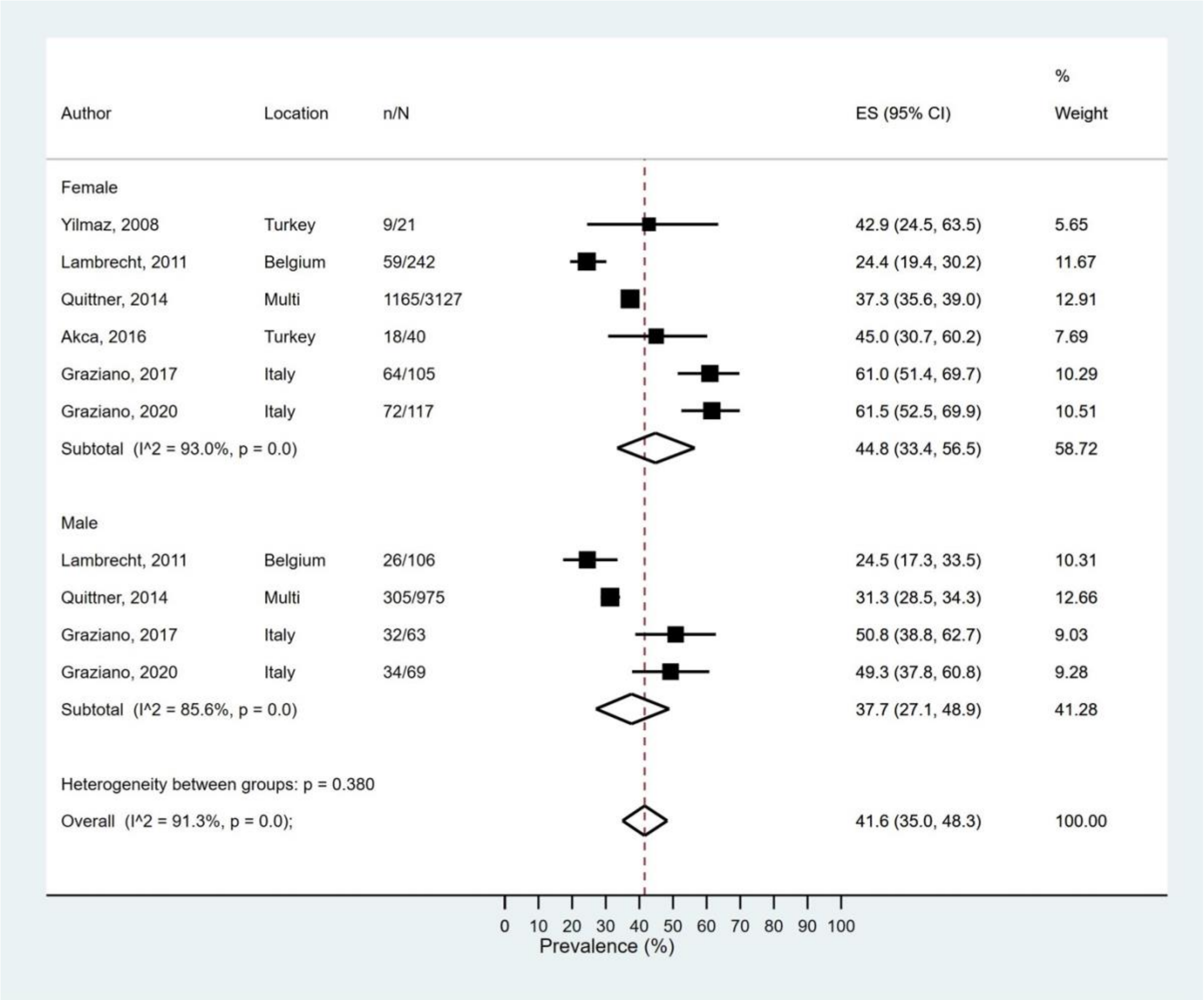

Figure S31. Forest plot of depression in caregivers of people with cystic fibrosis by caregiver sex

Abbreviations: CI: Confidence Interval; ES: Effect Size.

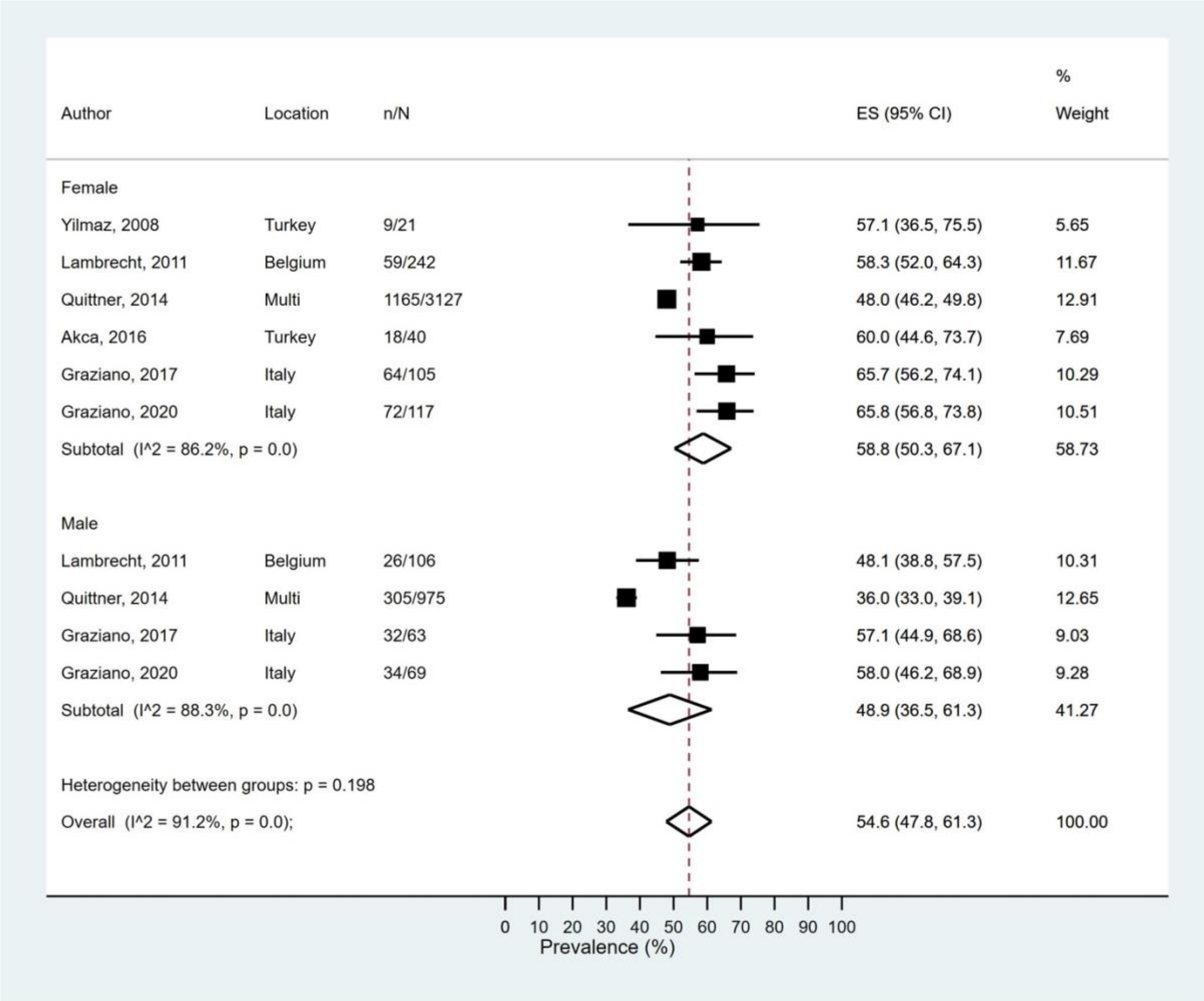

Figure S32. Forest plot of anxiety in caregivers of people with cystic fibrosis by caregiver sex

Abbreviations: CI: Confidence Interval; ES: Effect Size.

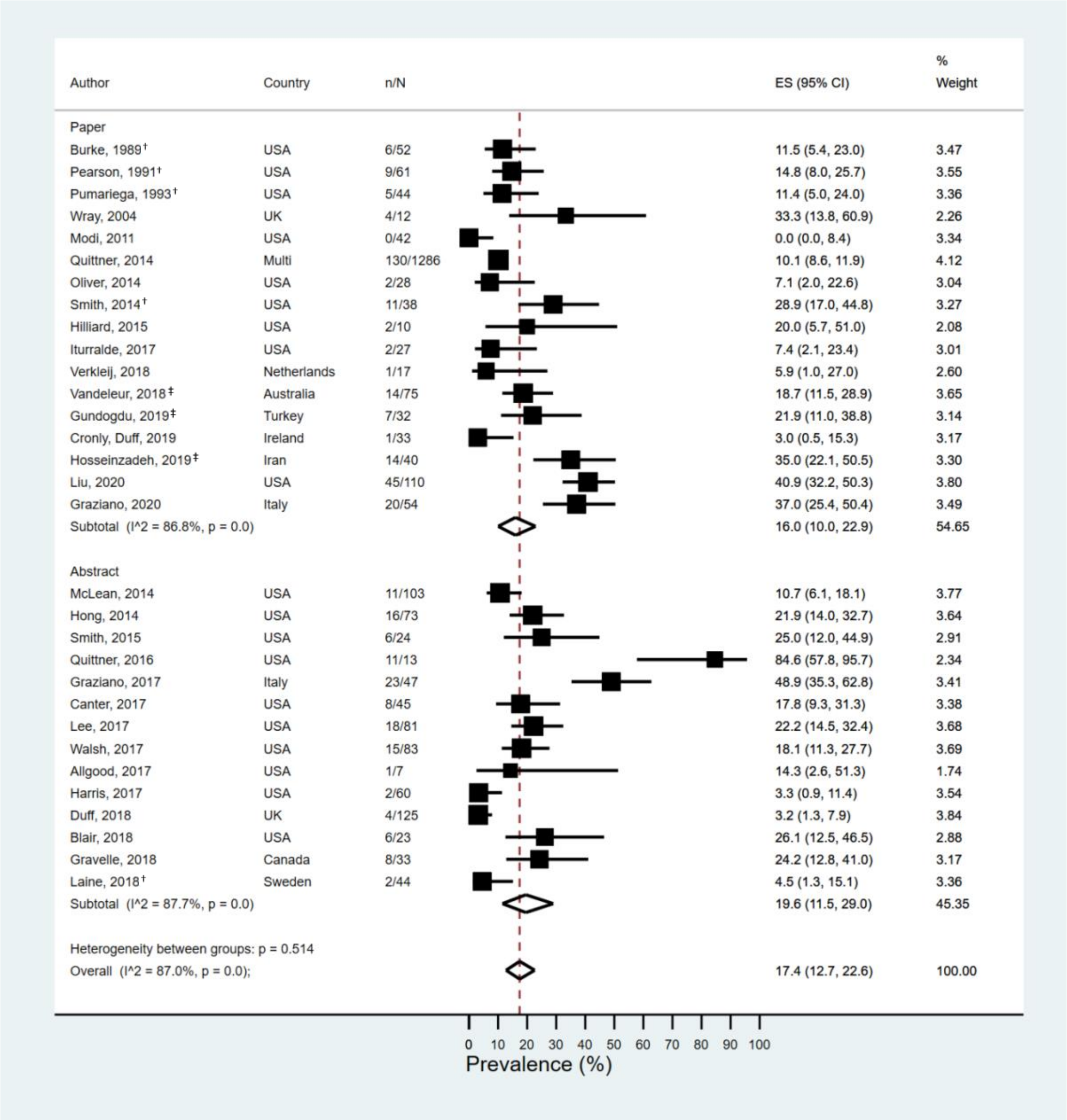

Figure S33. Forest plot of depression in children and adolescents with cystic fibrosis by publication type

Abbreviations: CI: Confidence Interval; ES: Effect Size; Paper: full-text publication. †Data from participants with an unclear age range (where participant age is not exclusive to either 5-11 or 12-18 years of age). ‡Data from children aged 5-11 years of age.

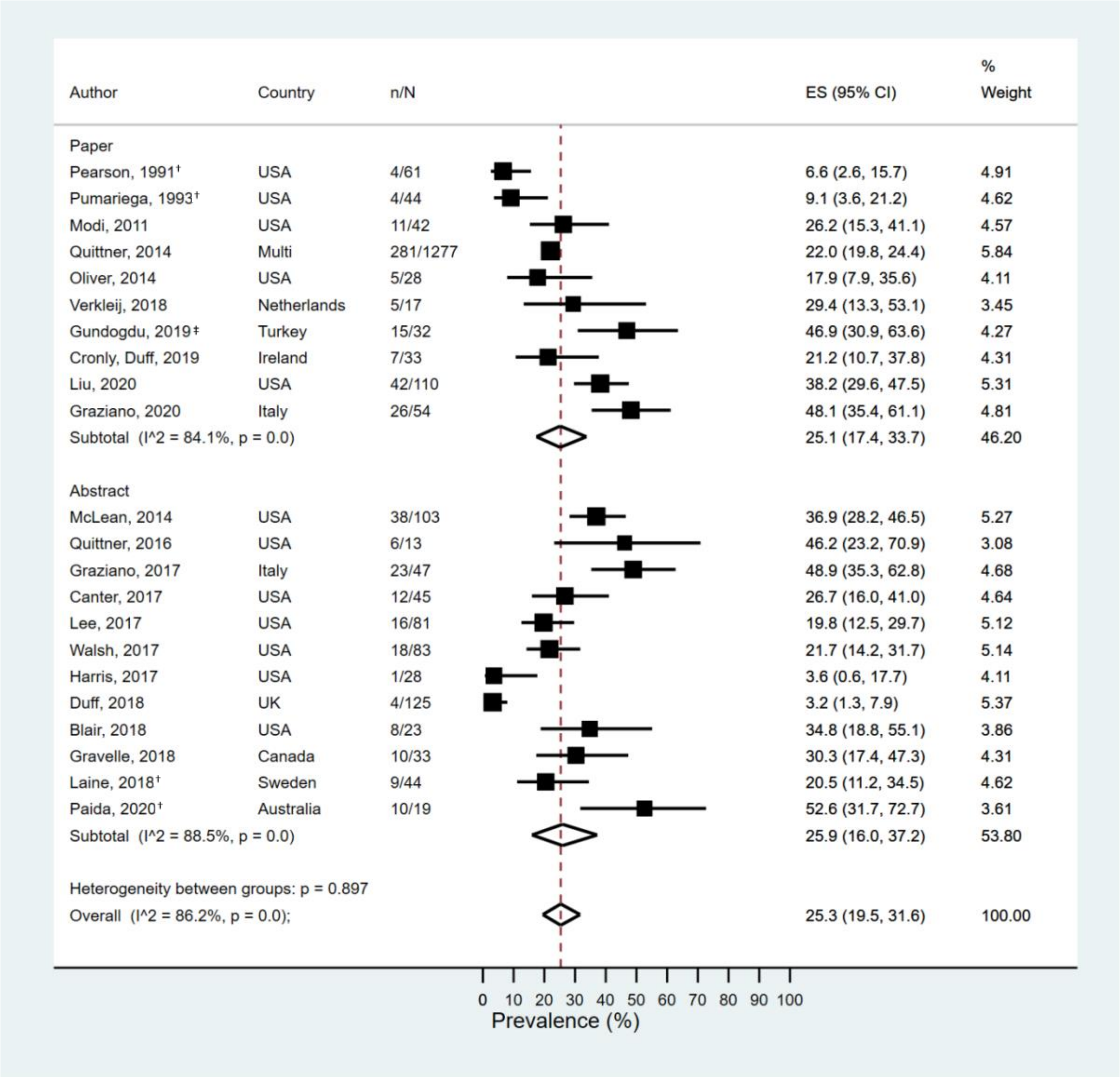

Figure S34. Forest plot of anxiety in children and adolescents with cystic fibrosis by publication type

Abbreviations: CI: Confidence Interval; ES: Effect Size; Paper: full-text publication. †Data from participants with an unclear age range (where participant age is not exclusive to either 5-11 or 12-18 years of age). ‡Data from children aged 5-11 years of age.

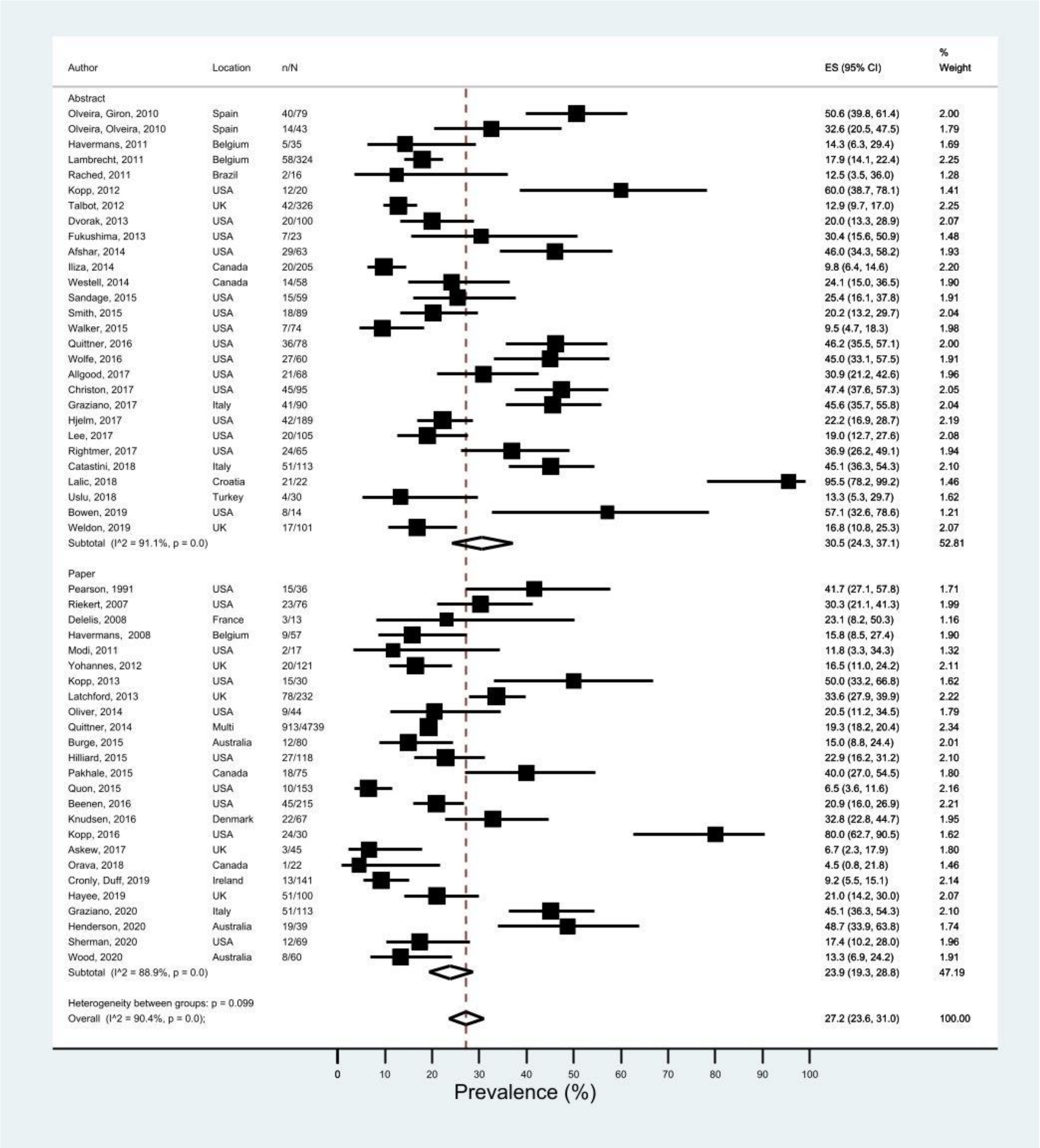

Figure S35. Forest plot of depression in adults with cystic fibrosis by publication type

Abbreviations: CI: Confidence Interval; ES: Effect Size; Paper: full-text publication.

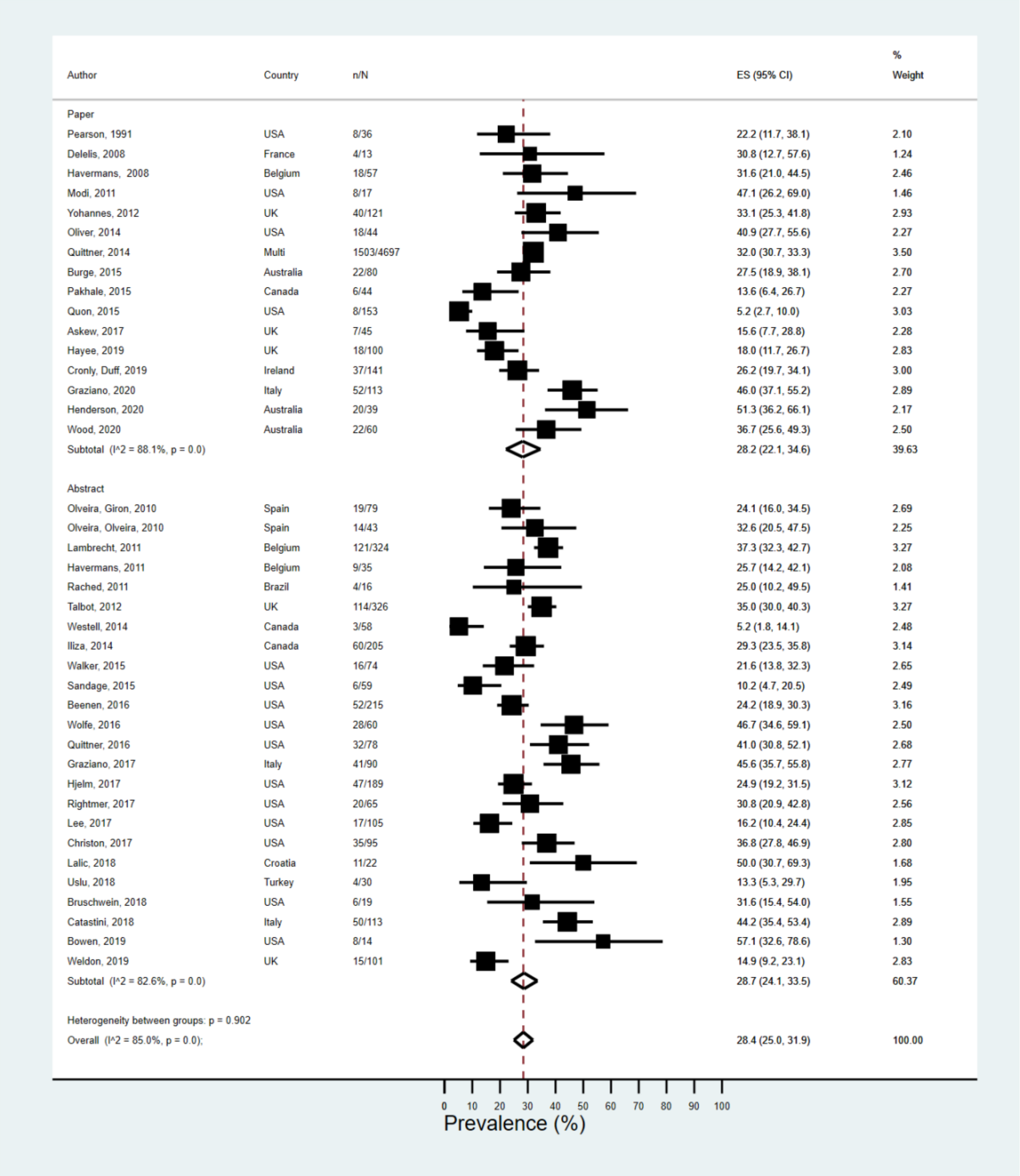

Figure S36. Forest plot of anxiety in adults with cystic fibrosis by publication type

Abbreviations: CI: Confidence Interval; ES: Effect Size; Paper: full-text publication.

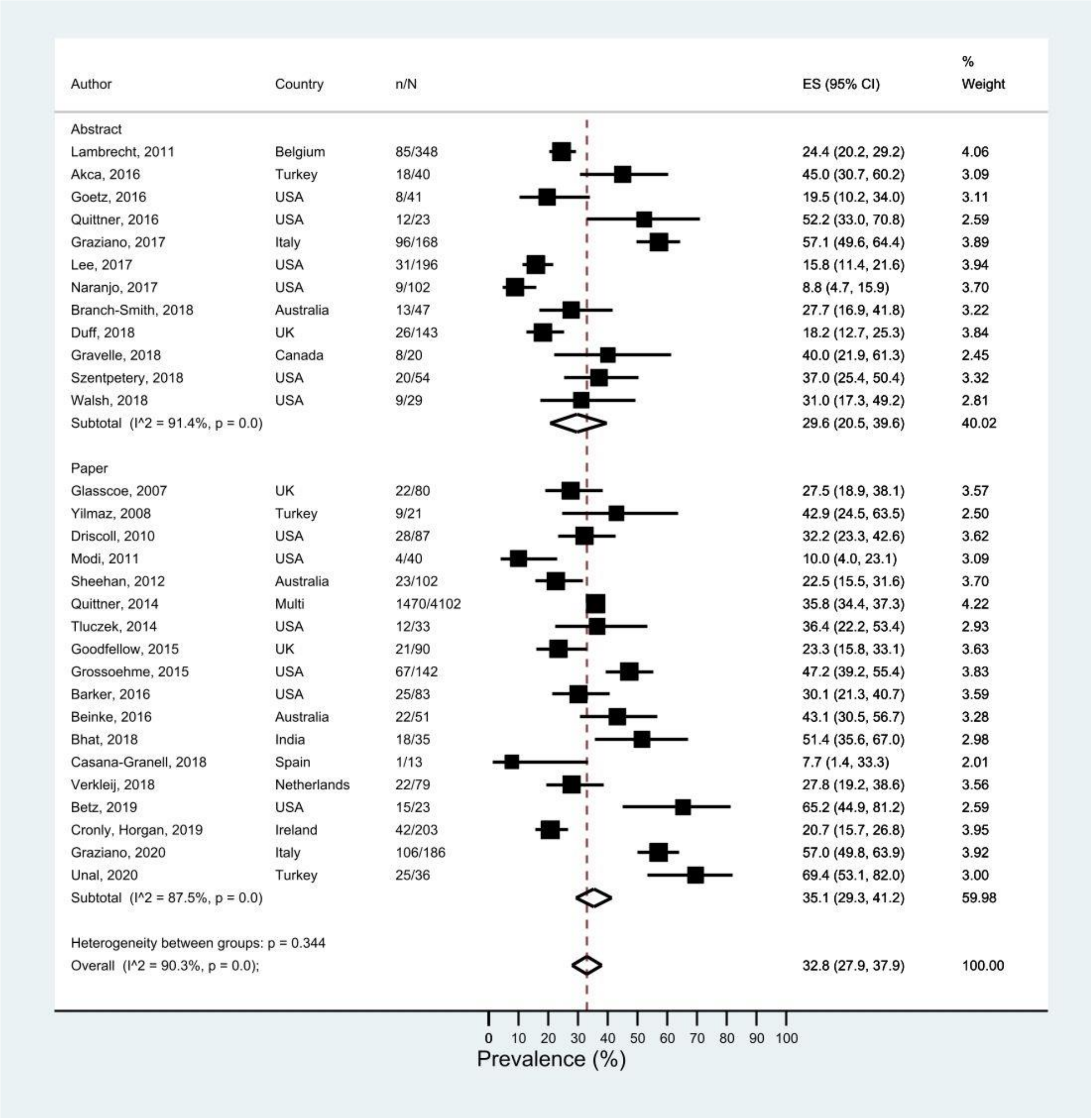

Figure S37. Forest plot of depression in caregivers of people with cystic fibrosis by publication type

Abbreviations: CI: Confidence Interval; ES: Effect Size; Paper: full-text publication.

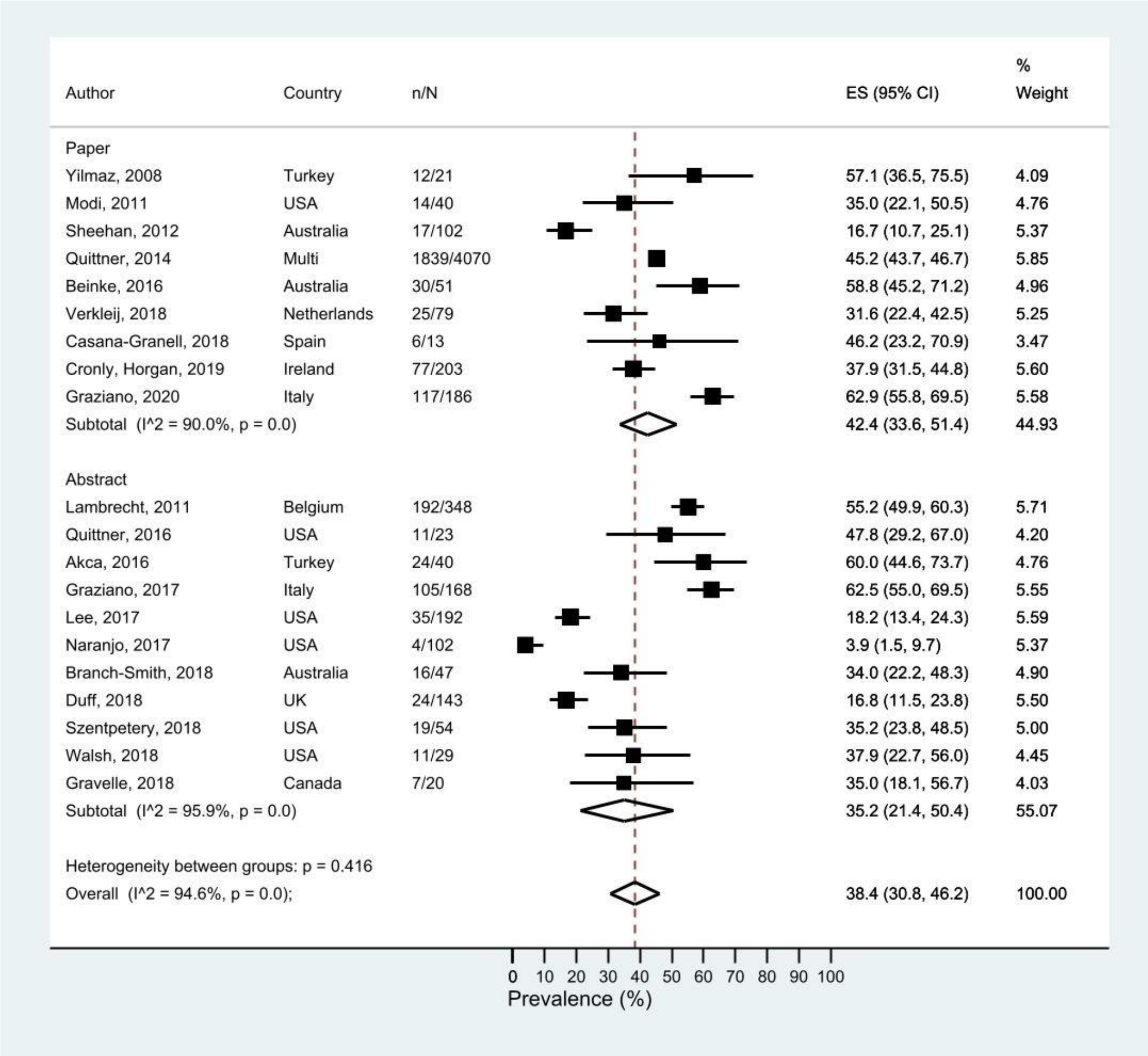

Figure S38. Forest plot of anxiety in caregivers of people with cystic fibrosis by publication type

Abbreviations: CI: Confidence Interval; ES: Effect Size; Paper: full-text publication.

Table S10. PRISMA Checklist

Section and Topic	Item #	Checklist item	Location where item is reported
**TITLE**			
Title	1	Identify the report as a systematic review.	Title
**ABSTRACT**			
Abstract	2	See the PRISMA 2020 for Abstracts checklist.	Abstract
**INTRODUCTION**			
Rationale	3	Describe the rationale for the review in the context of existing knowledge.	Introduction/1-3
Objectives	4	Provide an explicit statement of the objective(s) or question(s) the review addresses.	Introduction/3
**METHODS**			
Eligibility criteria	5	Specify the inclusion and exclusion criteria for the review and how studies were grouped for the syntheses.	Methods/3
Information sources	6	Specify all databases, registers, websites, organisations, reference lists and other sources searched or consulted to identify studies. Specify the date when each source was last searched or consulted.	Methods/2
Search strategy	7	Present the full search strategies for all databases, registers and websites, including any filters and limits used.	Supplementary information
Selection process	8	Specify the methods used to decide whether a study met the inclusion criteria of the review, including how many reviewers screened each record and each report retrieved, whether they worked independently, and if applicable, details of automation tools used in the process.	Methods/3-4
Data collection process	9	Specify the methods used to collect data from reports, including how many reviewers collected data from each report, whether they worked independently, any processes for obtaining or confirming data from study investigators, and if applicable, details of automation tools used in the process.	Methods/5-7
Data items	10a	List and define all outcomes for which data were sought. Specify whether all results that were compatible with each outcome domain in each study were sought (e.g. for all measures, time points, analyses), and if not, the methods used to decide which results to collect.	Methods/5-7
	10b	List and define all other variables for which data were sought (e.g. participant and intervention characteristics, funding sources). Describe any assumptions made about any missing or unclear information.	Methods/5-7
Study risk of bias assessment	11	Specify the methods used to assess risk of bias in the included studies, including details of the tool(s) used, how many reviewers assessed each study and whether they worked independently, and if applicable, details of automation tools used in the process.	Methods/6
Effect measures	12	Specify for each outcome the effect measure(s) (e.g. risk ratio, mean difference) used in the synthesis or presentation of results.	Methods/7
Synthesis methods	13a	Describe the processes used to decide which studies were eligible for each synthesis (e.g. tabulating the study intervention characteristics and comparing against the planned groups for each synthesis (item #5)).	Methods/7
	13b	Describe any methods required to prepare the data for presentation or synthesis, such as handling of missing summary statistics, or data	Methods/7

Section and Topic	Item #	Checklist item	Location where item is reported
		conversions.	
	13c	Describe any methods used to tabulate or visually display results of individual studies and syntheses.	Methods/7
	13d	Describe any methods used to synthesize results and provide a rationale for the choice(s). If meta-analysis was performed, describe the model(s), method(s) to identify the presence and extent of statistical heterogeneity, and software package(s) used.	Methods/7
	13e	Describe any methods used to explore possible causes of heterogeneity among study results (e.g. subgroup analysis, meta-regression).	Methods/7
	13f	Describe any sensitivity analyses conducted to assess robustness of the synthesized results.	Methods/7
Reporting bias assessment	14	Describe any methods used to assess risk of bias due to missing results in a synthesis (arising from reporting biases).	Methods/7
Certainty assessment	15	Describe any methods used to assess certainty (or confidence) in the body of evidence for an outcome.	Methods/7
**RESULTS**			
Study selection	16a	Describe the results of the search and selection process, from the number of records identified in the search to the number of studies included in the review, ideally using a flow diagram.	Results/1; Figure 1; Supplementary Information
	16b	Cite studies that might appear to meet the inclusion criteria, but which were excluded, and explain why they were excluded.	Figure 1
Study characteristics	17	Cite each included study and present its characteristics.	Table S1
Risk of bias in studies	18	Present assessments of risk of bias for each included study.	Table S5-S9; Figure S1-S6
Results of individual studies	19	For all outcomes, present, for each study: (a) summary statistics for each group (where appropriate) and (b) an effect estimate and its precision (e.g. confidence/credible interval), ideally using structured tables or plots.	Figure S13-S38
Results of syntheses	20a	For each synthesis, briefly summarise the characteristics and risk of bias among contributing studies.	Results/1-15
	20b	Present results of all statistical syntheses conducted. If meta-analysis was done, present for each the summary estimate and its precision (e.g. confidence/credible interval) and measures of statistical heterogeneity. If comparing groups, describe the direction of the effect.	Results/4-13; Figure 2-5; Figure S13-S38
	20c	Present results of all investigations of possible causes of heterogeneity among study results.	Results/3-13; Figure S19-S38
	20d	Present results of all sensitivity analyses conducted to assess the robustness of the synthesized results.	Results/6
Reporting biases	21	Present assessments of risk of bias due to missing results (arising from reporting biases) for each synthesis assessed.	Results/3; Table S5- S9;

Section and Topic	Item #	Checklist item	Location where item is reported
			Figure S1-S12
Certainty of evidence	22	Present assessments of certainty (or confidence) in the body of evidence for each outcome assessed.	Results; Figures S13-S38
**DISCUSSION**			
Discussion	23a	Provide a general interpretation of the results in the context of other evidence.	Discussion/1-5
	23b	Discuss any limitations of the evidence included in the review.	Discussion/6-7
	23c	Discuss any limitations of the review processes used.	Discussion/7
	23d	Discuss implications of the results for practice, policy, and future research.	Discussion1-7; Conclusion
**OTHER INFORMATION**			
Registration and protocol	24a	Provide registration information for the review, including register name and registration number, or state that the review was not registered.	Methods/1
	24b	Indicate where the review protocol can be accessed, or state that a protocol was not prepared.	Methods/1
	24c	Describe and explain any amendments to information provided at registration or in the protocol.	Not applicable
Support	25	Describe sources of financial or non-financial support for the review, and the role of the funders or sponsors in the review.	Funding
Competing interests	26	Declare any competing interests of review authors.	Conflict of interest
Availability of data, code and other materials	27	Report which of the following are publicly available and where they can be found: template data collection forms; data extracted from included studies; data used for all analyses; analytic code; any other materials used in the review.	Available on request from author

From: Page MJ, McKenzie JE, Bossuyt PM, Boutron I, Hoffmann TC, Mulrow CD, et al. The PRISMA 2020 statement: an updated guideline for reporting systematic reviews. BMJ 2021;372:n71. doi: 10.1136/bmj.n71

For more information, visit: http://www.prisma-statement.org/

References

1. Allgood SJ, Merlo C, Goss CH, Lechtzin N (2017) The association between pain and mortality in individuals with cystic fibrosis. Pediatr Pulmonol 52 (Supplement 47):405. doi:http://dx.doi.org/10.1002/ppul.23840
2. Blair SD, Hunt WR, Middour-Oxler B (2018) A retrospective cohort study exploring anxiety and depression in individuals with cystic fibrosis undergoing pediatric to adult care transition. Pediatric Pulmonology 53 (Supplement 2):432. doi:http://dx.doi.org/10.1002/ppul.24152
3. Burke P, Meyer V, Kocoshis S, Orenstein DM, Chandra R, Nord DJ, Sauer J, Cohen E (1989) Depression and anxiety in pediatric inflammatory bowel disease and cystic fibrosis. J Am Acad Child Adolesc Psychiatry 28 (6):948-951
4. Canter K, Keller J (2017) Implementation of mental health screening and first year results in the cf center at nemours/A.I. Dupont hospital for children. Pediatric Pulmonology 52 (Supplement 47):496. doi:http://dx.doi.org/10.1002/ppul.23840
5. Cronly JA, Duff AJ, Riekert KA, Fitzgerald AP, Perry IJ, Lehane EA, Horgan A, Howe BA, Chroinin MN, Savage E (2019) Health-related quality of life in adolescents and adults with cystic fibrosis: physical and mental health predictors. Respiratory Care, vol 64. doi:http://dx.doi.org/10.4187/respcare.06356
6. Duff AJ, Latchford G, Fisher C, Vuister T (2018) Assessing strengths and difficulties as part of annual mental health screening. J Cyst Fibros 17:S51. doi:10.1016/S1569-1993(18)30278-9
7. Gravelle A, Kolb T, Jenkins S, McMahon V, Chilvers M (2018) Instituting mental health screening in a Canadian paediatric cystic fibrosis clinic. J Cyst Fibros 17 (Supplement 3):S52. doi:http://dx.doi.org/10.1016/S1569-1993(18)30281-9
8. Graziano S, Majo F, Spano B, Gentile S, Lucidi V, Tabarini P (2017) Mental health and health outcomes in patients with cystic fibrosis and parent caregivers. Pediatr Pulmonol 52 (Supplement 47):474-475. doi:http://dx.doi.org/10.1002/ppul.23840
9. Graziano S, Spanò B, Majo F, Righelli D, Vincenzina L, Quittner A, Tabarini P (2020) Rates of depression and anxiety in Italian patients with cystic fibrosis and parent caregivers: Implementation of the Mental Health Guidelines. Respir Med 172:106147. doi:https://doi.org/10.1016/j.rmed.2020.106147
10. Gundogdu U, Fis NP, Eralp EE, Karadag BT (2019) Major depression and psychiatric comorbidity in Turkish children and adolescents with cystic fibrosis. Pediatr Pulmonol 54 (12):1927-1935. doi:https://dx.doi.org/10.1002/ppul.24492
11. Harris M, Lois B, Giusti R, Mavaro C, Delgado D, Sklenar D, Liaw R (2017) NYU langone pediatric cystic fibrosis depression & anxiety screening initiative: Ongoing assessment and risk identification. Pediatric Pulmonology 52 (Supplement 47):488-489. doi:http://dx.doi.org/10.1002/ppul.23840
12. Hilliard ME, Eakin MN, Borrelli B, Green A, Riekert KA (2015) Medication beliefs mediate between depressive symptoms and medication adherence in cystic fibrosis. Health Psychol 34 (5):496-504. doi:https://dx.doi.org/10.1037/hea0000136
13. Hong G, Allgood S, Riekert KA, Hankinson JC, Rivera T, Mogayzel PJ, Lechtzin N (2014) The association between pain and depression in adolescents with cystic fibrosis. American Journal of Respiratory and Critical Care Medicine Conference: American Thoracic Society International Conference, ATS 189 (MeetingAbstracts)
14. Hosseinzadeh SS, Rafeey M, Vahedi L, Noorazar SG (2019) The frequency of psychiatric disorders in cystic fibrosis patients aged 5-18 years in northwest of Iran. Crescent Journal of Medical and Biological Sciences 6 (1):56-60
15. Iturralde E, Adams RN, Barley RC, Bensen R, Christofferson M, Hanes SJ, Maahs DM, Milla C, Naranjo D, Shah AC, Tanenbaum ML, Veeravalli S, Park K, Hood KK (2017) Implementation of depression screening and global health assessment in pediatric subspecialty clinics. J Adolesc Health 61 (5):591-598
16. Laine C, Bergenmar Ivarsson E, Larsson P (2018) Outcome mental screening among Swedish adolescents with CF. Journal of Cystic Fibrosis 17:S52. doi:https://doi.org/10.1016/S1569-1993(18)30282-0
17. Lee A, Somervell E, Palmrose W, Allada G, Powers MR (2017) Mental health screening implementation in pediatric and adult clinic setting. Pediatr Pulmonol 52 (Supplement 47):490. doi:http://dx.doi.org/10.1002/ppul.23840
18. Liu FF, Lew A, Andes E, McNamara S, Cassidy J, Whitmore S, Plunkett R, Ong T (2020) Implementation strategies for depression and anxiety screening in a pediatric cystic fibrosis center: A quality improvement project. Pediatric pulmonology. doi:10.1002/ppul.24951
19. McLean KA, Madan A, Monzon A, Quittner AL (2014) Trajectories of depression and anxiety in adolescents with CF. Pediatric Pulmonology 49:442. doi:http://dx.doi.org/10.1002/ppul.23108

20. Modi AC, Driscoll KA, Montag-Leifling K, Acton JD (2011) Screening for symptoms of depression and anxiety in adolescents and young adults with cystic fibrosis. Pediatr Pulmonol 46 (2):153-159. doi:https://dx.doi.org/10.1002/ppul.21334
21. Oliver KN, Free ML, Bok C, McCoy KS, Lemanek KL, Emery CF (2014) Stigma and optimism in adolescents and young adults with cystic fibrosis. J Cyst Fibros 13 (6):737-744. doi:https://dx.doi.org/10.1016/j.jcf.2014.04.005
22. Paida K, McKay IR, Coffey MJ, Kasparian N, Katz T, Ooi CY (2020) Evaluation of anxiety levels in a paediatric cystic fibrosis population. Journal of Cystic Fibrosis 19:S160-S161. doi:https://doi.org/10.1016/S1569-1993(20)30702-5
23. Pearson DA, Pumariega AJ, Seilheimer DK (1991) The development of psychiatric symptomatology in patients with cystic fibrosis. J Am Acad Child Adolesc Psychiatry 30 (2):290-297. doi:http://dx.doi.org/10.1097/00004583-199103000-00019
24. Pumariega AJ, Pearson DA, Seilheimer DK (1993) Family and childhood adjustment in cystic fibrosis. Journal of Child and Family Studies 2 (2):109-118
25. Quittner AL, Goldbeck L, Abbott J, Duff A, Lambrecht P, Solé A, Tibosch MM, Bergsten Brucefors A, Yüksel H, Catastini P, Blackwell L, Barker D (2014) Prevalence of depression and anxiety in patients with cystic fibrosis and parent caregivers: results of The International Depression Epidemiological Study across nine countries. Thorax 69 (12):1090. doi:10.1136/thoraxjnl-2014-205983
26. Quittner AL, Saez-Flores E, Nicolais CJ, Pedreira PB, Colin A, Salathe M (2016) Screening depression and anxiety in patients with cystic fibrosis and parent caregivers: Preliminary results from a pilot program at pediatric and adult CF centers. J Cyst Fibros 15 (Supplement 1):S113-S114. doi:http://dx.doi.org/10.1016/S1569-1993(16)30484-2
27. Smith BA, Cogswell A, Garcia G (2014) Vitamin D and depressive symptoms in children with cystic fibrosis. Psychosomatics 55 (1):76-81. doi:https://dx.doi.org/10.1016/j.psym.2013.01.012
28. Smith BA, Roach C, Cogswell A (2015) Does depression screening and stepped care treatment improve mental health outcomes in a CF center? Pediatric Pulmonology 50:423. doi:http://dx.doi.org/10.1002/ppul.23297
29. Vandeleur M, Walter LM, Armstrong DS, Robinson P, Nixon GM, Horne RSC (2018) Quality of life and mood in children with cystic fibrosis: Associations with sleep quality. Journal of Cystic Fibrosis 17 (6):811-820. doi:https://dx.doi.org/10.1016/j.jcf.2017.11.021
30. Verkleij M, de Winter D, Hurley MA, Abbott J (2018) Implementing the International Committee on Mental Health in Cystic Fibrosis (ICMH) guidelines: Screening accuracy and referral-treatment pathways. J Cyst Fibros 17 (6):821-827. doi:http://dx.doi.org/10.1016/j.jcf.2018.02.005
31. Walsh K, Hunt S, Bass SK, Ren CL (2017) Screening and treatment of anxiety and depression in a pediatric cystic fibrosis clinic. Pediatric Pulmonology 52 (Supplement 47):485. doi:http://dx.doi.org/10.1002/ppul.23840
32. Wray J, Radley-Smith R (2004) Depression in pediatric patients before and 1 year after heart or heart-lung transplantation. J Heart Lung Transplant 23 (9):1103-1110
33. Afshar K, Patel P, Fukushima L, Haile M, Rao AP (2014) Severity of PHQ-9 score and rate of pulmonary exacerbations in adults with cystic fibrosis. Pediatric Pulmonology 49:447. doi:http://dx.doi.org/10.1002/ppul.23108
34. Askew K, Bamford J, Hudson N, Moratelli J, Miller R, Anderson A (2017) Current characteristics, challenges and coping strategies of young people with cystic fibrosis as they transition to adulthood. Clin Med 17 (2):121-125. doi:10.7861/clinmedicine.17-2-121
35. Beenen M, Holzworth L, Dunitz JM (2016) Adult mental health screening implementation. Pediatric Pulmonology 51 (Supplement 45):467-468. doi:http://dx.doi.org/10.1002/ppul.23576
36. Bowen M, King C, Duong Q, Russell C, Connors G, Kopelen R, Palczynski K, Brown W (2019) Outcomes and impact of a CF-specific pulmonary rehabilitation program. Pediatric Pulmonology 54 (Supplement 2):381-382. doi:http://dx.doi.org/10.1002/ppul.22495
37. Bruschwein H, Soper M, Albon D (2018) Anxiety screening during medical admission for cystic fibrosis exacerbation. Pediatric Pulmonology 53 (Supplement 2):415-416. doi:http://dx.doi.org/10.1002/ppul.24152
38. Burge AT, Holland AE, Sherburn M, Wilson J, Cox NS, Rasekaba TM, McAleer R, Morton JM, Button BM (2015) Prevalence and impact of urinary incontinence in men with cystic fibrosis. Physiotherapy 101 (2):166-170. doi:https://dx.doi.org/10.1016/j.physio.2014.11.001
39. Catastini P, De Masi S, Braggion C (2018) How and to what extent we can know our patient's emotional state? Italian Journal of Pediatrics Conference: 23rd Italian congress of Cystic Fibrosis and the 13th National Congress of Cystic Fibrosis Italian Society Italy 44 (Supplement 1). doi:http://dx.doi.org/10.1186/s13052-017-0430-4

40. Christon LM, Brown K, Bishop SM, Balliet W, Borckardt J, Taylor BJ, Gray S, Flume P (2017) CFSeniors: Mental health screening in older adult patients with cystic fibrosis. Pediatric Pulmonology 52 (Supplement 47):495. doi:http://dx.doi.org/10.1002/ppul.23840
41. Delelis G, Christophe V, Leroy S, Vanneste J, Wallaert B (2008) The effects of cystic fibrosis on couples: marital satisfaction, emotions, and coping strategies. Scand J Psychol 49 (6):583-589. doi:https://dx.doi.org/10.1111/j.1467-9450.2008.00683.x
42. Dvorak M, Gesley K, Mohabir PK (2013) Cystic fibrosis and depression: Routine standardized screening leads to early referral for therapy and a reduction in the 1-year prevalence. Pediatric Pulmonology 48:422-423. doi:http://dx.doi.org/10.1002/ppul.22898
43. Fukushima L, Patel P, Afshar K, Evangelista LG, Rao A, Hammond T (2013) Fatigue in adult CF patients: May be associated with sleep habits. Pediatric Pulmonology 48:438. doi:http://dx.doi.org/10.1002/ppul.22898
44. Havermans T, Colpaert K, Dupont LJ (2008) Quality of life in patients with Cystic Fibrosis: association with anxiety and depression. J Cyst Fibros 7 (6):581-584. doi:10.1016/j.jcf.2008.05.010
45. Havermans T, Lambrecht P, Dupont L (2011) Changes in anxiety and depression in relation to deteriorating disease. Pediatric Pulmonology 46:408-409. doi:http://dx.doi.org/10.1002/ppul.21583
46. Hayee B, Watson KL, Campbell S, Simpson A, Farrell E, Hutchings P, Macedo P, Perrin F, Whelan K, Elston C (2019) A high prevalence of chronic gastrointestinal symptoms in adults with cystic fibrosis is detected using tools already validated in other GI disorders. United European Gastroenterol J 7 (7):881-888. doi:10.1177/2050640619841545
47. Henderson D, Moore V, MacMorran K, Castellini J, Hay K, Keegan V, Reid D, Curtin D, Tay G (2020) A Cohort Study of Sleep Quality in Adult Patients with Acute Pulmonary Exacerbations of Cystic Fibrosis. Internal Medicine Journal n/a (n/a). doi:10.1111/imj.15082
48. Hjelm M, Orkis A, Duncan J, Jackson C, Seymour D, Heintz JS (2017) Mental health screening as part of routine adult cystic fibrosis visits: A quality improvement project. Pediatric Pulmonology 52 (Supplement 47):441. doi:http://dx.doi.org/10.1002/ppul.23840
49. Iliza AC, Berthiaume Y, Jeanneret A, Lavoie-Pilote A, Choiniere M (2014) Characteristics and impact of chronic pain in adults with cystic fibrosis. Pediatric Pulmonology 49:442. doi:http://dx.doi.org/10.1002/ppul.23108
50. Knudsen KB, Pressler T, Mortensen LH, Jarden M, Skov M, Quittner AL, Katzenstein T, Boisen KA (2016) Associations between adherence, depressive symptoms and health-related quality of life in young adults with cystic fibrosis. Journal of Cystic Fibrosis 15 (Supplement 1):S9
51. Kopp BT, Baron N, Splaingard M (2012) Lighting up depression in cystic fibrosis. Pediatric Pulmonology 47:431. doi:http://dx.doi.org/10.1002/ppul.22682
52. Kopp BT, Hayes D, Jr., Ratkiewicz M, Baron N, Splaingard M (2013) Light exposure and depression in hospitalized adult patients with cystic fibrosis. J Affect Disord 150 (2):585-589. doi:https://dx.doi.org/10.1016/j.jad.2013.02.014
53. Kopp BT, Hayes D, Jr., Ghera P, Patel A, Kirkby S, Kowatch RA, Splaingard M (2016) Pilot trial of light therapy for depression in hospitalized patients with cystic fibrosis. J Affect Disord 189:164-168. doi:https://dx.doi.org/10.1016/j.jad.2015.08.056
54. Lalic I, Dugac AV, Zovko T, Sajnic A, John V, Mustac A, Tepavac M, Drinkovic DT, Samarzija M (2018) Does lung transplantation affect on the improvement of psychological status in patients with adult cystic fibrosis? European Respiratory Journal Conference: European Respiratory Society International Congress, ERS 52 (Supplement 62). doi:http://dx.doi.org/10.1183/13993003.congress2018.PA1505
55. Lambrecht P (2011) Anxiety and depression in Belgian patients with cystic fibrosis (CF) and their parents: A major national epidemiological study. J Cyst Fibros 10:S91. doi:http://dx.doi.org/10.1016/S1569-1993%2811%2960371-8
56. Latchford G, Duff AJ (2013) Screening for depression in a single CF centre. J Cyst Fibros 12 (6):794-796. doi:10.1016/j.jcf.2013.04.002
57. Olveira G, Giron RM, Olveira C, Escobedo MC, Espildora F, Juan DS, Gaspar I (2010) Depression and anxiety in patients with cystic fibrosis in Spain. Journal of Cystic Fibrosis 9:S96. doi:http://dx.doi.org/10.1016/S1569-1993%2810%2960371-2
58. Olveira G, Olveira C, Espildora F, Antonio D, Padilla A, Gaspar I, De La Cruz JL (2010) Depression and anxiety in cystic fibrosis: Relation with quality of life. Journal of Cystic Fibrosis 9:S97. doi:http://dx.doi.org/10.1016/S1569-1993%2810%2960375-X
59. Orava C, Fitzgerald J, Figliomeni S, Lam D, Naccarato A, Szego E, Yoshida K, Fox P, Sykes J, Wu K (2018) Relationship between Physical Activity and Fatigue in Adults with Cystic Fibrosis. Physiotherapy Canada 70 (1):42-48. doi:10.3138/ptc.2016-75

60. Pakhale S, Baron J, Armstrong M, Tasca G, Gaudet E, Aaron S, Cameron W, Balfour L (2015) A Cross-Sectional Study of the Psychological Needs of Adults Living with Cystic Fibrosis. PLoS One 10 (6):e0127944. doi:10.1371/journal.pone.0127944
61. Quon BS, Bentham WD, Unutzer J, Chan YF, Goss CH, Aitken ML (2015) Prevalence of symptoms of depression and anxiety in adults with cystic fibrosis based on the PHQ-9 and GAD-7 screening questionnaires. Psychosomatics 56 (4):345-353. doi:10.1016/j.psym.2014.05.017
62. Rached S, Athanazio R, Angelini L, Prieto J, Cukier A, Carvalho-Pinto R, Stelmach R (2011) Anxiety, depression, and health related quality of life in a bronchiectasis population-a comparison between cystic fibrosis and non-cystic fibrosis adult patients. Chest Conference: CHEST 140 (4 MEETING ABSTRACT). doi:http://dx.doi.org/10.1378/chest.1119089
63. Riekert KA, Bartlett SJ, Boyle MP, Krishnan JA, Rand CS (2007) The association between depression, lung function, and health-related quality of life among adults with cystic fibrosis. Chest 132 (1):231-237. doi:10.1378/chest.06-2474
64. Rightmer N, Mulenos AM, Iezzi S, Stewart J, Britto C, Talwalkar J, Lee H, Koff J, Fucito L (2017) Implementation of cystic fibrosis mental health guidelines at an adult cystic fibrosis program. Pediatric Pulmonology 52 (Supplement 47):501. doi:http://dx.doi.org/10.1002/ppul.23840
65. Sandage D, Roboff J, Petty C, Sawicki GS, Berger R, DiTullio N, Cardoni L, Frances O, Sharma N, Uluer AZ (2015) Evaluation of transition readiness and mental health among patients with CF admitted to a young adult unit. Pediatric Pulmonology 50:442. doi:http://dx.doi.org/10.1002/ppul.23297
66. Sherman AC, Simonton-Atchley S, O'Brien CE, Campbell D, Reddy RM, Guinee B, Wagner LD, Anderson PJ (2020) Longitudinal associations between gratitude and depression 1 year later among adult cystic fibrosis patients. J Behav Med 43 (4):596-604. doi:10.1007/s10865-019-00071-y
67. Talbot S, Pryce S, Bilton D, Madge S (2012) Factors that increase depression in adults with cystic fibrosis. Journal of Cystic Fibrosis 11:S137. doi:http://dx.doi.org/10.1016/S1569-1993%2812%2960484-6
68. Uslu NZ, Yildizeli SO, Kocakaya D, Eryuksel E, Ceyhan B (2018) Coping in adult cystic fibrosis patients: Association with anxiety and depression. European Respiratory Journal Conference: European Respiratory Society International Congress, ERS 52 (Supplement 62). doi:http://dx.doi.org/10.1183/13993003.congress-2018.PA1336
69. Walker P, Berdella M, Fresenius A, Balzano J, Chen J, Langfelder-Schwind E, Wilder K, Chu A, Plachta A, Glajchen M, Bookbinder M, Portenoy R, Dhingra L (2015) Implementation of a web-based monthly screening tool to ascertain burden of illness and improve access to services for patients with cystic fibrosis. Pediatric Pulmonology 50:440. doi:http://dx.doi.org/10.1002/ppul.23297
70. Weldon P, Cove J, Maguire J (2019) Psychological resilience in adults with cystic fibrosis. Journal of Cystic Fibrosis 18 (Supplement 1):S27-S28. doi:http://dx.doi.org/10.1016/S1569-1993%2819%2930204-8
71. Westell S, Dang K, Thomas A, Tullis E (2014) Improving the quality of cystic fibrosis mental health through the introduction of validated screening tools. Pediatric Pulmonology 49:394-395. doi:http://dx.doi.org/10.1002/ppul.23108
72. Wolfe W, O'Hayer CV, Taylor D, Stephen MJ (2016) Acceptance and commitment therapy with cystic fibrosis: A telehealth pilot study. Pediatric Pulmonology 51 (Supplement 45):466. doi:http://dx.doi.org/10.1002/ppul.23576
73. Wood J, Jenkins S, Putrino D, Mulrennan S, Morey S, Cecins N, Bear N, Hill K (2020) A smartphone application for reporting symptoms in adults with cystic fibrosis improves the detection of exacerbations: Results of a randomised controlled trial. J Cyst Fibros 19 (2):271-276. doi:10.1016/j.jcf.2019.09.002
74. Yohannes AM, Willgoss TG, Fatoye FA, Dip MD, Webb K (2012) Relationship between anxiety, depression, and quality of life in adult patients with cystic fibrosis. Respir Care 57 (4):550-556. doi:10.4187/respcare.01328
75. Akca OF, Fakultesi MT, Uzun N, Pekcan S, Akkus E, Gulec K (2016) Caregiver burden and related factors in mothers of children and adolescents with cystic fibrosis. Journal of the American Academy of Child and Adolescent Psychiatry 55 (10 Supplement 1):S208. doi:http://dx.doi.org/10.1016/j.jaac.2016.09.333
76. Barker DH, Quittner AL (2016) Parental Depression and Pancreatic Enzymes Adherence in Children With Cystic Fibrosis. Pediatrics 137 (2):24-24. doi:10.1542/peds.2015-2296
77. Beinke K, O'Callaghan F, Morrissey S (2017) Illness Perceptions of Cystic Fibrosis: A Comparison of Young Adults with CF and Same-Aged Peers. Behavioral Medicine 43 (1):40-46
78. Betz J, Szczesniak R, Lewis K, Pestian T, Bennethum A, McBride J, Grossoehme D (2019) Feasibility and Acceptability of a Telephone-Based Chaplaincy Intervention to Decrease Parental Spiritual Struggle. Journal of Religion and Health 58 (6):2065-2085. doi:10.1007/s10943-019-00921-8

79. Bhat JI, Wani WA, Charoo BA, Ali SW, Ahmad QI, Ahangar AA (2018) Prevalence of Depression among Caregivers of Indian Children with Cystic Fibrosis. Indian J Pediatr 85 (11):974-977. doi:https://dx.doi.org/10.1007/s12098-018-2695-z
80. Branch-Smith C, Barrett A, Balding E, Grover Z, Yikilmaz G, Bourke C, Depiazzi J, Sander N, Foster J, Zepf F, et al. (2018) Treating unresolved grief in parents of children with cystic fibrosis. Journal of cystic fibrosis 17:S53-
81. Casana-Granell S, Lacomba-Trejo L, Valero-Moreno S, Prado-Gasco V, Montoya-Castilla I, Perez-Marin M (2018) A brief version of the Pediatric Inventory for Parents (PIP) in Spanish population: Stress of main family carers of chronic paediatric patients. PLoS ONE [Electronic Resource] 13 (7):e0201390. doi:https://dx.doi.org/10.1371/journal.pone.0201390
82. Cronly J, Horgan AM, Lehane E, Howe B, Duff AJ, Riekert KA, Perry IJ, Fitzgerald AP, Chroinin MN, Savage E (2019) Anxiety and Depression in Parent Caregivers of Children with Cystic Fibrosis. Journal of Child and Family Studies 28 (5):1304-1312
83. Driscoll KA, Johnson SB, Barker D, Quittner AL, Deeb LC, Geller DE, Gondor M, Silverstein JH (2010) Risk factors associated with depressive symptoms in caregivers of children with type 1 diabetes or cystic fibrosis. J Pediatr Psychol 35 (8):814-822. doi:https://dx.doi.org/10.1093/jpepsy/jsp138
84. Glasscoe C, Lancaster GA, Smyth RL, Hill J (2007) Parental depression following the early diagnosis of cystic fibrosis: a matched, prospective study. J Pediatr 150 (2):185-191
85. Goetz DM, Rand K, Roach CM, Cogswell A, Smith BA (2016) Screening for depression in caregivers of childrens with cystic fibrosis. Pediatric Pulmonology 51 (Supplement 45):456. doi:http://dx.doi.org/10.1002/ppul.23576
86. Goodfellow NA, Hawwa AF, Reid AJ, Horne R, Shields MD, McElnay JC (2015) Adherence to treatment in children and adolescents with cystic fibrosis: a cross-sectional, multi-method study investigating the influence of beliefs about treatment and parental depressive symptoms. BMC polm 15:43. doi:https://dx.doi.org/10.1186/s12890-015-0038-7
87. Grossoehme DH, Szczesniak RD, Britton LL, Siracusa CM, Quittner AL, Chini BA, Dimitriou SM, Seid M (2015) Adherence Determinants in Cystic Fibrosis: Cluster Analysis of Parental Psychosocial, Religious, and/or Spiritual Factors. Annals of the American Thoracic Society 12 (6):838-846. doi:https://dx.doi.org/10.1513/AnnalsATS.201408-379OC
88. Naranjo D, Barley RC, Veeravalli S, Hernandez C, Adams R, Milla C (2017) Mental health screening feasibility and outcomes in a large pediatric CF center. Pediatr Pulmonol 52 (Supplement 47):499-500. doi:http://dx.doi.org/10.1002/ppul.23840
89. Sheehan J, Massie J, Hay M, Jaffe A, Glazner J, Armstrong D, Hiscock H (2012) The natural history and predictors of persistent problem behaviours in cystic fibrosis: a multicentre, prospective study. Arch Dis Child 97 (7):625-631
90. Szentpetery SE, Foil K, Christon LM (2018) Relationship of caregiver depression and anxiety to pediatric cystic fibrosis health outcomes. Pediatric Pulmonology 53 (Supplement 2):432-433. doi:http://dx.doi.org/10.1002/ppul.24152
91. Tluczek A, Laxova A, Grieve A, Heun A, Brown RL, Rock MJ, Gershan WM, Farrell PM (2014) Long-term follow-up of cystic fibrosis newborn screening: psychosocial functioning of adolescents and young adults. Journal of Cystic Fibrosis 13 (2):227-234. doi:https://dx.doi.org/10.1016/j.jcf.2013.10.001
92. Unal Yuksekgonul A, Aslan AT, Sismanlar Eyuboglu T, Soysal S, Budakoglu, II (2020) Evaluation of the psychological status of mothers of children with cystic fibrosis and the relationship between children's clinical status. J Paediatr Child Health. doi:10.1111/jpc.14983
93. Walsh K, Hunt S, Ren C Mental health screening in parents of infants diagnosed with CF throughout newborn screening. In: PEDIATRIC PULMONOLOGY, 2018. WILEY 111 RIVER ST, HOBOKEN 07030-5774, NJ USA, pp 430-431
94. Yilmaz O, Sogut A, Gulle S, Can D, Ertan P, Yuksel H (2008) Sleep quality and depression-anxiety in mothers of children with two chronic respiratory diseases: asthma and cystic fibrosis. Journal of Cystic Fibrosis 7 (6):495-500. doi:https://dx.doi.org/10.1016/j.jcf.2008.05.002
95. Naing L, Winn T, Rusli B (2006) Practical issues in calculating the sample size for prevalence studies. Archives of Orofacial Sciences 1:9-14
